# A long noncoding RNA distributed in both nucleus and cytoplasm operates in the PYCARD-regulated apoptosis by coordinating the epigenetic and translational regulation

**DOI:** 10.1371/journal.pgen.1008144

**Published:** 2019-05-14

**Authors:** Hui Miao, Linlin Wang, Haomiao Zhan, Jiangshan Dai, Yanbo Chang, Fan Wu, Tao Liu, Zhongyu Liu, Chunfang Gao, Ling Li, Xu Song

**Affiliations:** 1 Center for Functional Genomics and Bioinformatics, Key Laboratory of Bio-Resource and Eco-Environment of Ministry of Education, College of Life Sciences, Sichuan University, Chengdu, Sichuan, China; 2 College of Basic Science and Forensic Medicine, Sichuan University, Chengdu, Sichuan, China; 3 989 Hospital, Luoyang, Henan, China; 4 State Key Laboratory of Biotherapy, West China Hospital, Sichuan University, Chengdu, Sichuan, China; UNITED STATES

## Abstract

Long noncoding RNAs (lncRNAs) participate in various biological processes such as apoptosis. The function of lncRNAs is closely correlated with their localization within the cell. While regulatory potential of many lncRNAs has been revealed at specific subcellular location, the biological significance of discrete distribution of an lncRNA in different cellular compartments remains largely unexplored. Here, we identified an lncRNA antisense to the pro-apoptotic gene *PYCARD*, named PYCARD-AS1, which exhibits a dual nuclear and cytoplasmic distribution and is required for the *PYCARD* silencing in breast cancer cells. The *PYCARD*-regulated apoptosis is controlled by PYCARD-AS1; moreover, PYCARD-AS1 regulates apoptosis in a *PYCARD*-dependent manner, indicating that *PYCARD* is a critical downstream target of PYCARD-AS1. Mechanistically, PYCARD-AS1 can localize to the *PYCARD* promoter, where it facilitates DNA methylation and H3K9me2 modification by recruiting the chromatin-suppressor proteins DNMT1 and G9a. Moreover, PYCARD-AS1 and PYCARD mRNA can interact with each other via their 5′ overlapping region, leading to inhibition of ribosome assembly in the cytoplasm for *PYCARD* translation. This study reveals a mechanism whereby an lncRNA works at different cellular compartments to regulate the pro-apoptotic gene *PYCARD* at both the epigenetic and translational levels, contributing to the *PYCARD*-regulated apoptosis, and also sheds new light on the role of discretely distributed lncRNAs in diverse biological processes.

## Author summary

As the reveal of tens of thousands of long noncoding RNAs (lncRNAs) from mammalian genomes, there is increasing interest to understand how these transcripts function in physiological and pathological processes. The regulatory potential of many lncRNAs has been revealed at specific subcellular location. Nevertheless, many lncRNAs are discretely distributed in different cellular compartments, and the related biological significance remains largely unclear. PYCARD-AS1 is such a “discrete” lncRNA, which exhibits a dual nuclear and cytoplasmic localization. Here, we provide a comprehensive picture of how PYCARD-AS1 works at different cellular compartments to regulate the pro-apoptotic gene *PYCARD* at both the epigenetic and translational levels, thereby contributing to the apoptotic regulation. Our study adds a different layer to the lncRNA-mediated regulation of gene expression, and strongly suggests that the transcripts of a specific lncRNA may have distinct functionalities at different cellular compartments where they are located.

## Introduction

The great majority of mammalian genomes are pervasively transcribed, giving rise to tens of thousands of noncoding transcripts, especially long noncoding RNAs (lncRNAs) [1]. LncRNAs participate in a large range of biological processes such as cell differentiation, apoptosis and proliferation, and most of them function by participating in the regulation of gene expression [2, 3]. Unlike mRNAs, which must be localized to the cytoplasm for protein synthesis, lncRNAs exhibit diverse subcellular distribution patterns, ranging from predominant nuclear foci to almost exclusively cytoplasmic localization, and exert distinct regulatory effects at their particular site of action [4, 5]. Thus, the subcellular localization of lncRNAs is critical for their biological function.

A number of studies have implicated the regulatory potential of lncRNAs at specific subcellular location. Determined by the site of action where they are located, lncRNAs may work *in cis* on neighboring genes or *in trans* to regulate distantly located genes or molecular targets in the nucleus or cytoplasm [5, 6]. Also, our related studies showed that several nuclear lncRNAs drive tumorigenesis by sequestering the activity of PSF protein in repression of proto-oncogene transcription [7, 8], and revealed that the OCC-1 transcripts localized in cytoplasm inhibit cell cycle transition by modulating the stability of HuR protein [9]. Current studies on lncRNAs have greatly advanced our knowledge of their physiological and pathological roles. Nevertheless, a substantial proportion of lncRNAs are revealed to exhibit a discrete subcellular distribution [10], and the biological significance of discrete distribution of an lncRNA in different cellular compartments remains largely unexplored.

The pro-apoptotic gene *PYCARD* encodes a signaling factor that consists of an N-terminal PYRIN-PAAD-DAPIN domain (PYD) and a C-terminal caspase-recruitment domain (CARD) and operates in the intrinsic and extrinsic cell death pathways [11, 12]. *PYCARD* was originally identified to undergo the DNA methyltransferase-1 (DNMT1)-mediated epigenetic silencing in a wide range of human tumors [13–17], and subsequent studies showed that its inactivation is also associated with other epigenetic events such as H3K9 dimethylation [18]. In this study, we further report an lncRNA antisense to *PYCARD*, named PYCARD-AS1, which exhibits a dual nuclear and cytoplasmic distribution and is required for the *PYCARD* silencing in breast cancer cells. PYCARAD-AS1 is functionally involved in the *PYCARD*-regulated apoptosis, and regulates apoptosis in a *PYCARD*-dependent manner, indicating that *PYCARD* is a critical function mediator of PYCARD-AS1. Mechanistically, PYCARD-AS1 not only acts *in cis* to facilitate the recruitment of DNMT1 and histone H3K9 methyltransferase G9a to the *PYCARD* locus, but also inhibits ribosome assembly in the cytoplasm for *PYCARD* translation, leading to coordinated regulation of *PYCARD* expression at the epigenetic and translational levels. Our findings highlight the notion that the transcripts of a specific lncRNA may operate in biological processes by exerting distinct regulatory effects at different cellular compartments.

## Results

### PYCARD-AS1 is an lncRNA distributed in both nucleus and cytoplasm

**Fig 1:**
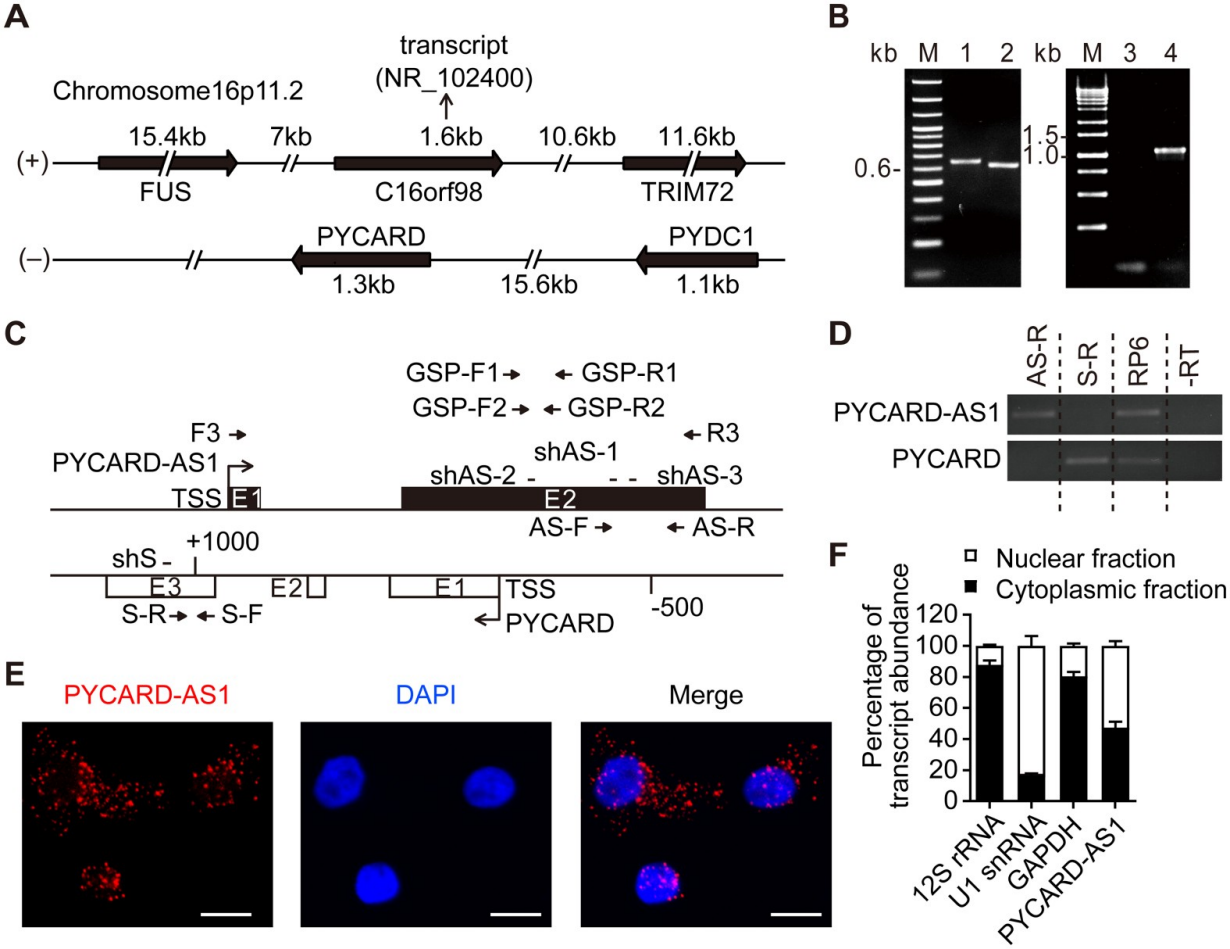
PYCARD-AS1 is an lncRNA distributed in both nucleus and cytoplasm. **(A)** Schematic illustration of genomic loci of *C16orf98*, *PYCARD* and three neighboring genes on human chromosome 16p11.2. *C16orf98*, *FUS* and *TRIM72* are encoded by the (+) DNA strand, while *PYCARD* and *PYDC1* are encoded by the (-) DNA strand. **(B)** The length of PYCARD-AS1 determined by 5′ and 3′ RACE assays. Lane 1, 3′ RACE product; lane 2, 5′ RACE product; lane 3, RT-PCR reaction omitting reverse transcriptase as a negative control; lane 4, full-length PYCARD-AS1. **(C)** Exon composition of the PYCARD-AS1 and PYCARD transcripts. Rectangles represent exons and arrows mark TSSs. Short lines show location of the PYCARD-AS1- and PYCARD-specific shRNAs (shAS and shS, respectively). Arrows also indicate the primers used for RACE (GSP-F1 and GSP-F2 for 3′ RACE, GSP-R1 and GSP-R2 for 5′ RACE) and RT-PCR (F3 and R3) in (B), and the primers used for the subsequent qRT-PCR detecting the abundance of PYCARD-AS1 (AS-F and AS-R) and PYCARD (S-F and S-R) transcripts. AS-R and S-R were also used to initiate the RT reaction in directional RT-PCR in (D). **(D)** Directional RT-PCR experiments detecting the PYCARD-AS1 and PYCARD transcripts. RNA extracted from SKBR3 cells was reverse-transcribed using gene-specific reverse primers (AS-R and S-R), and the resulting cDNA was subjected to a PCR amplification that involves a primer pair corresponding to the non-overlapping regions of PYCARD-AS1 and PYCARD. RT reaction initiated by random hexamers (RP6) was set up as a positive control, and RT reaction omitting reverse transcriptase (-RT) was included as a negative control. **(E)** RNA FISH detecting the distribution of PYCARD-AS1 in SKBR3 cells (scale bars, 10 μm). **(F)** qRT-PCR assay following nuclear/cytoplasmic fractionation detecting the distribution of the indicated transcripts in SKBR3 cells. The qRT-PCR data, represented as a percentage of the total amount of detected transcripts, are presented as means ± SD from three independent experiments performed in triplicate.

Through a GenBank search, we identified the gene *C16orf98* on the opposite strand of the *PYCARD* gene, which produces a transcript in a head-to-head orientation relative to the PYCARD mRNA (Fig 1A). Based on the sequence of *C16orf98*, the experiments of 5′- and 3′-RACE were initiated with total RNA from SKBR3 cells and resulted in a 1,095-nucleotide (nt) antisense transcript of *PYCARD* (Fig 1B), which is the same as the transcript annotated as PYCARD-AS1. PYCARD-AS1 originates in the second intron 892 nt downstream of the transcription start site (TSS) of *PYACRD*, ends at nt 676 upstream of the *PYCARD* TSS, and consists of two exons (Fig 1C). A “directional” RT-PCR assay, which was set up by using gene-specific reverse primers in RT reaction (AS-R and S-R, shown in Fig 1C), showed that neither *PYCARD-AS1* nor *PYCARD* produces longer RNA species spanning their 3’ non-overlapping regions (Fig 1D). In addition, the RT-PCR detection initiated by different primers (S1A Fig), as well as the 3′-RACE experiment, indicated that PYCARD-AS1 is poly(A)-tailed, belonging to an mRNA-like transcript. We next examined the sensitivity of PYCARD-AS1 transcription to the Pol II inhibitor α-amanitin. Following α-amanitin treatment, the levels of PYCARD-AS1 and ACTB transcripts were reduced, whereas those of pre-tRNA^tyr^ and 45S pre-rRNA showed no change (S1B Fig), indicating that PYCARD-AS1 is transcribed by Pol II.

PYCARD-AS1 is annotated as a noncoding transcript in GenBank. In addition, the Coding Potential Calculator tool [19] predicted that PYCARD-AS1 displays no protein-coding potentiality (S1C Fig). Although UniProt showed a putative protein prediction of 204 amino acids for PYCARD-AS1 (S1D Fig), we found that the putative open reading frame (ORF) of PYCARD-AS1 could not be expressed as an N-terminal enhanced green fluorescent protein (EGFP) fusion protein (S1E and S1F Fig). By RNA FISH assay, PYCARD-AS1 was revealed to exhibit a dual nuclear and cytoplasmic distribution (Fig 1E). Moreover, cellular fractionation assay showed that the distribution of PYCARD-AS1 is clearly distinct from that of the nuclear-localized U1 snRNA, the mitochondrially retained 12S rRNA and the protein-coding GAPDH mRNA (Fig 1F). We further separated the chromatin fraction, and found that approximately 25% of nuclear-localized PYCARD-AS1 transcripts were chromatin-enriched (S1G Fig).

### PYCARD-AS1 is a negative regulator of *PYCARD*

**Fig 2:**
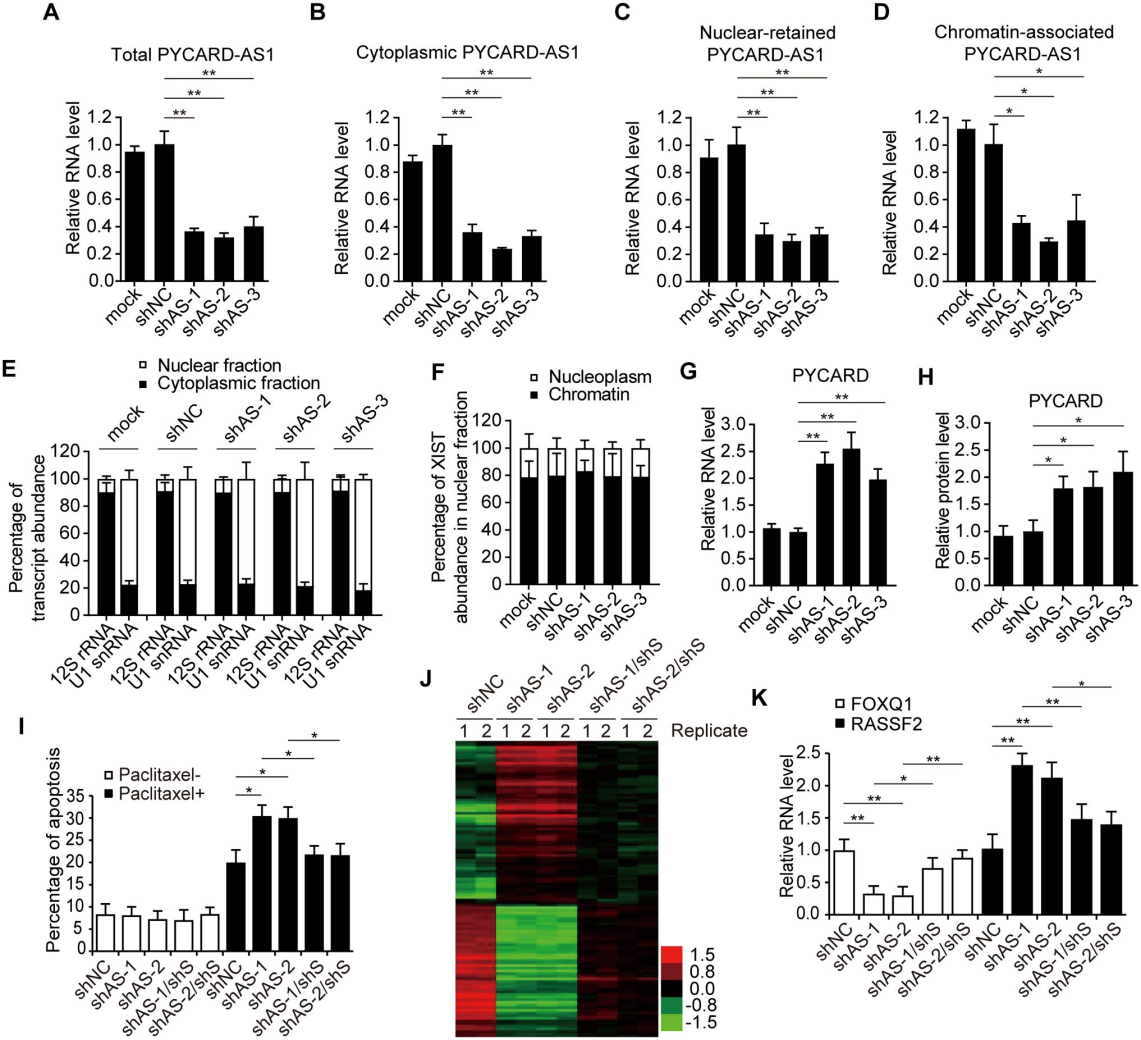
PYCARD-AS1 is a negative regulator of *PYCARD*. **(A–G)** qRT-PCR performed in SKBR3 cells to confirm the downregulation of total (A), cytoplasmic (B), nuclear-retained (C) and chromatin-associated (D) PYCARD-AS1 by three different shRNAs, and to detect the effect of PYCARD-AS1 knockdown on *PYCARD* expression (G). ACTB mRNA, 12S rRNA, U1 snRNA, XIST RNA, and ACTB mRNA were included in (A, B, C, D, and G) as an internal control to normalize the amount of RNA in the samples. Distribution of 12S rRNA, U1 snRNA (E) and XIST RNA (F) was tested to confirm the cellular fractionation in (B–D). Data are presented as means ± SD from three independent experiments performed in triplicate; *p < 0.05; **p < 0.01. **(H)** Immunoblotting detecting the effect of PYCARD-AS1 knockdown on *PYCARD* expression in SKBR3 cells. ACTB was used as an internal control to normalize the amount of total protein in the samples. PYCARD immunoblotting signals from three independent assays (S2B Fig) were subjected to densitometric analysis, and the data are presented as means ± SD; *p < 0.05. **(I)** PYCARD-AS1 affects the sensitivity of SKBR3 cells to paclitaxel-induced apoptosis in a *PYCARD*-dependent manner. The indicated SKBR3 cells were subjected to annexin V/propidium iodide staining followed by flow cytometry analysis, and the rates of apoptosis were analyzed. Data are presented as means ± SD from three independent experiments; *p < 0.05. Like *PYCARD*, PYCARD-AS1 showed no effect on apoptosis in the absence of cytotoxic agent [21]. **(J)** Heat map representation of microarray data from two independent experiments on the gene expression levels in the indicated shRNA-treated SKBR3 cells (*p* < 0.05). **(K)** qRT-PCR of *FOXQ1* and *RASSF2*, two PYCARD-AS1 and PYCARD-controlled genes, in the indicated SKBR3 cells. Data are presented as means ± SD from three independent experiments performed in triplicate; *p < 0.05; **p < 0.01.

Since antisense lncRNAs have been implicated as regulator of their sense counterparts [2, 5], we set out to analyze whether PYCARD-AS1 can regulate *PYCARD* expression. The transcription level of PYCARD-AS1 was first measured in four breast cancer cell lines, namely, SKBR3, MCF7, MDA-MB-231 and T47D. *PYCARD-AS1* transcription was detectable in all cell lines, with SKBR3 expressing it most (S2A Fig). Next, shRNAs were designed to silence PYCARD-AS1 and detect the effect of PYCARD-AS1 knockdown on *PYCARD* expression in SKBR3 cells. The shRNAs were shown to reduce the levels of total and cytoplasmic PYCARD-AS1 and efficiently act on the nuclear-retained and chromatin-associated PYCARD-AS1 (Fig 2A–2F). Concomitantly, we observed that PYCARD-AS1 silencing increases the PYCARD mRNA and protein levels (Fig 2G and 2H and S2B Fig), indicating a negative regulation of *PYCARD* by PYCARD-AS1. In parallel, we knocked down PYCARD mRNA with specific shRNA, and found that PYCARD silencing didn’t change the PYCARD-AS1 level (S2C Fig). In addition, PYCARD-AS1 knockdown was shown to have no effect on the mRNA levels of *FUS*, *TRIM72* and *PYDC1*, three genes neighboring *PYCARD* and *PYCARD-AS1* (Fig 1A and S2D Fig), suggesting that PYCARD-AS1 specifically regulates *PYCARD*. Also, the negative *PYCARD* regulation by PYCARD-AS1 was confirmed in MCF7, MDA-MB-231 and T47D cells (S2E–S2G Fig).

The silencing of *PYCARD* is closely correlated with the defective apoptosis of tumor cells, and its reactivation was revealed to increase the susceptibility of tumor cells to cytotoxic agents [20]. Confirming the regulatory effect of PYCARD-AS1 on *PYCARD* expression prompted assays on the contribution of PYCARD-AS1 to *PYCARD*-regulated apoptosis. We tested whether PYCARD-AS1 regulates the sensitivity of SKBR3 cells to paclitaxel, which is also associated with the expression level of *PYCARD* [21]. Knockdown of PYCARD-AS1 increased *PYCARD* expression (S2H Fig), and the PYCARD-AS1-knockdown SKBR3 cells showed increased sensitivity to paclitaxel treatment compared with the control cells (Fig 2I and S2I Fig). Remarkably, simultaneous PYCARD knockdown, which neutralized the increased *PYCARD* expression (S2H Fig), was shown to largely weaken the sensitivity of SKBR3 cells to paclitaxel increased by PYCARD-AS1 silencing (Fig 2I and S2I Fig), suggesting that PYCARD-AS1 regulates apoptosis in a *PYCARD*-dependent manner.

SKBR3 cells included in the above apoptosis assay were also subjected to global gene expression analysis. Microarray data revealed hundreds of genes that were induced or suppressed more than twofold as a consequence of PYCARD-AS1 knockdown, and showed that the expression of a significant proportion of these differentially expressed genes (91 of 175 PYCARD-AS1 knockdown-induced genes and 37 of 59 PYCARD-AS1 knockdown-suppressed genes) could be reversed at least 1.5-fold in PYCARD-AS1 and PYCARD double-knockdown SKBR3 cells compared with that in PYCARD-AS1 knockdown SKBR3 cells (Fig 2J and S1 Table; GEO accession number: GSE85032). Several target genes identified by microarray were randomly selected for qRT-PCR confirmation (Fig 2K and S2J Fig). The above data indicate that PYCARD-AS1 is involved in the *PYCARD*-regulated apoptosis, and that *PYCARD* is a critical downstream target of PYCARD-AS1.

### PYCARD-AS1 regulates *PYCARD* at the epigenetic level

**Fig 3:**
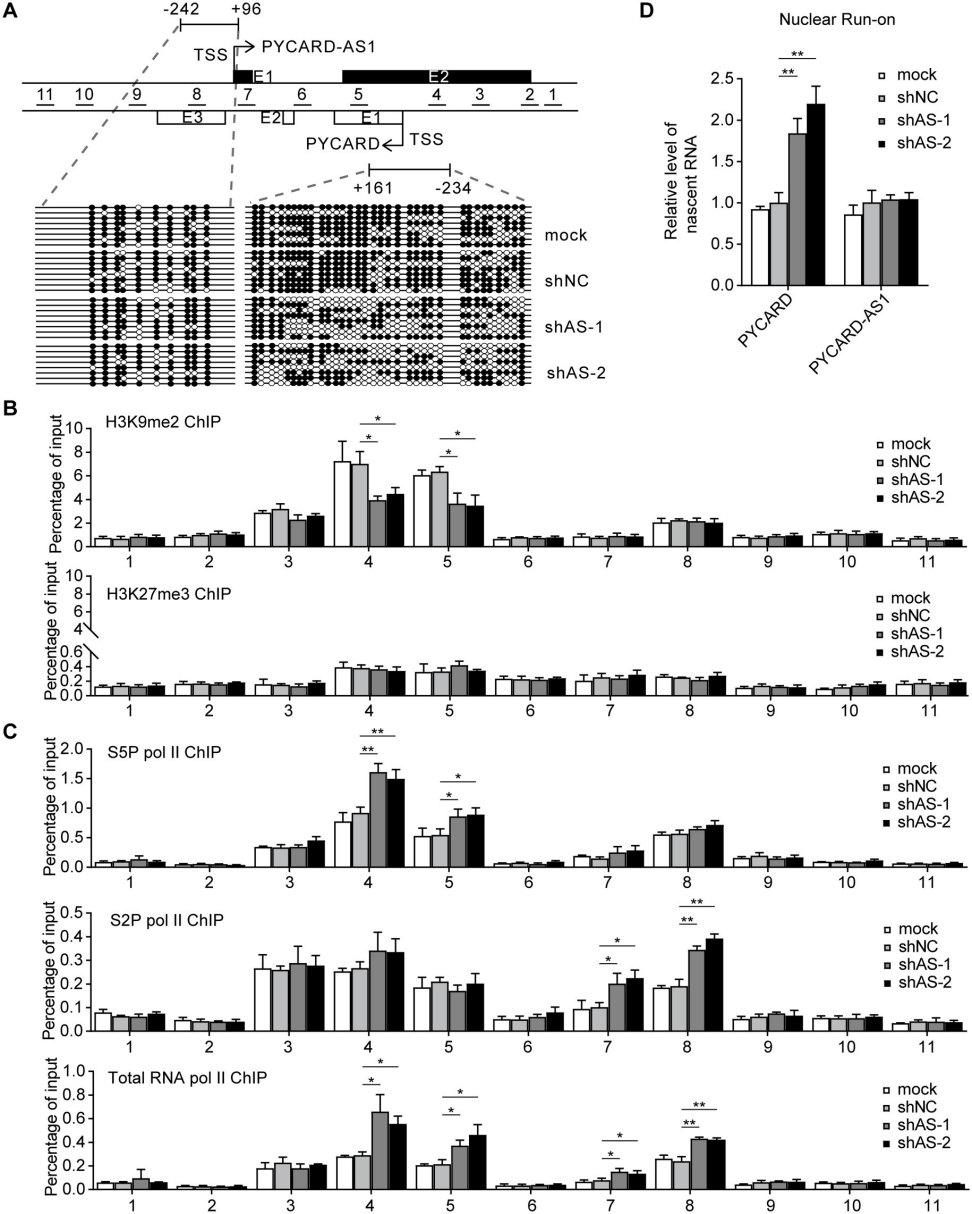
PYCARD-AS1 regulates *PYCARD* at the epigenetic level. **(A)** DNA methylation status of the *PYCARD-AS1* (left) and *PYCARD* (right) promoter regions in SKBR3 cells with or without PYCARD-AS1 knockdown. Genomic DNA modified with bisulfite was amplified with primer sets that span a *PYCARD* (nt −234/+161 with respect to the *PYCARD* TSS) or *PYCARD-AS1* (nt −242/+96 with respect to the *PYCARD-AS1* TSS) promoter region. The two regions harbor 34 and 10 CpG sites, respectively. Each row indicates the sequence of an independent amplicon where methylated (black circles) and unmethylated (white circles) CpG sites are indicated. The short lines represent the segments that were detected by the ChIP-based tiling assays in Fig 3 and Fig 4 and S3 Fig. Relative to the *PYCARD* or *PYCARD-AS1* TSS, their locations are nt -871/-763 (+1655/+1763), nt -716/-572 (+1464/+1608), nt -459/-338 (+1230/+1351), nt -280/-64 (+956/+1172), nt +130/+279 (+614/+763), nt +442/+574 (+319/+451), nt +778/+889 (+4/+115), nt +1027/+1130 (-238/-135), nt +1291/+1443 (-551/-399), nt +1537/+1722 (-830/-645) and nt +1837/+1998 (-1106/-945). **(B, C)** ChIP assays detecting the occupancy of H3K9me2 (B, upper), H3K27me3 (B, lower), serine 5-phosphorylated initiating Pol II (S5P Pol II) (C, upper), serine 2-phosphorylated elongating Pol II (S2P Pol II) (C, middle) and total RNA polymerase II (total RNA Pol II) (C, lower) on the segments shown in (A) in SKBR3 cells with or without PYCARD-AS1 knockdown. The data of retrieved DNA segments, represented as a percentage of the amount input, are presented as means ± SD from three independent experiments performed in triplicate; * *p* < 0.05; ** *p* < 0.01. **(D)** Nuclear run-on assay detecting the impact of PYCARD-AS1 knockdown on *PYCARD* and *PYCARD-AS1* transcription in SKBR3 cells. ACTB mRNA was used as an internal control to normalize the amount of total RNA in the samples. Data are presented as means ± SD from three independent experiments performed in triplicate; **p < 0.01.

Following on from the above studies, we attempted to determine how PYCARD-AS1 suppresses *PYCARD* by using SKBR3 cells. DNA methylation and histone H3K9 dimethylation are two types of epigenetic modifications contributing to the *PYCARD* silencing in tumor cells [13, 18]. Since shRNAs were shown to act on the PYCARD-AS1 in nucleus (Fig 2C and 2D), we first analyzed whether PYCARD-AS1 knockdown can impact the methylation status of the *PYCARD* promoter, with the *PYCARD-AS1* promoter being detected as a control. The results from bisulfite sequencing showed that the *PYCARD* promoter, but not the *PYCARD-AS1* promoter, was partially demethylated by PYCARD-AS1 knockdown (Fig 3A), leading to a relatively hypomethylated state. We also performed ChIP assay to examine the distribution of H3K9me2 across a genomic region of approximately 2.7 kb that covers the *PYCARD* and *PYCARD-AS1* loci. The decrease of H3K9me2 occupancy, which occurred upon PYCARD-AS1 knockdown, was only detected in the region near the *PYCARD* TSS (Fig 3B, upper). Histone modification H3K27me3 was included as a ChIP control, and the result showed that it was rarely enriched across this genomic region compared with H3K9me2 (Fig 3B, lower).

As detected by Pol II ChIP assay, PYCARD-AS1 knockdown also resulted in an elevation in the initiating Pol II occupancy at the 5′ *PYCARD* region, as well as an elevation in the elongating Pol II occupancy at the 3′ *PYCARD* region and the total Pol II occupancy at the 5′ and 3′ *PYCARD* regions (Fig 3C), indicating an enhanced *PYCARD* transcription initiation. The effect of PYCARD-AS1 on *PYCARD* transcription was further dissected via nuclear run-on assay, and the results revealed an increased production of nascent transcript for *PYCARD* when PYCARD-AS1 was knocked down (Fig 3D). Altogether, the findings described above suggest that PYCARD-AS1 knockdown in SKBR3 cells changes the chromatin status from an inactive state to an active one, thereby inducing *PYCARD* transcription.

### PYCARD-AS1 recruits DNMT1 and G9a to *PYCARD* promoter

**Fig 4:**
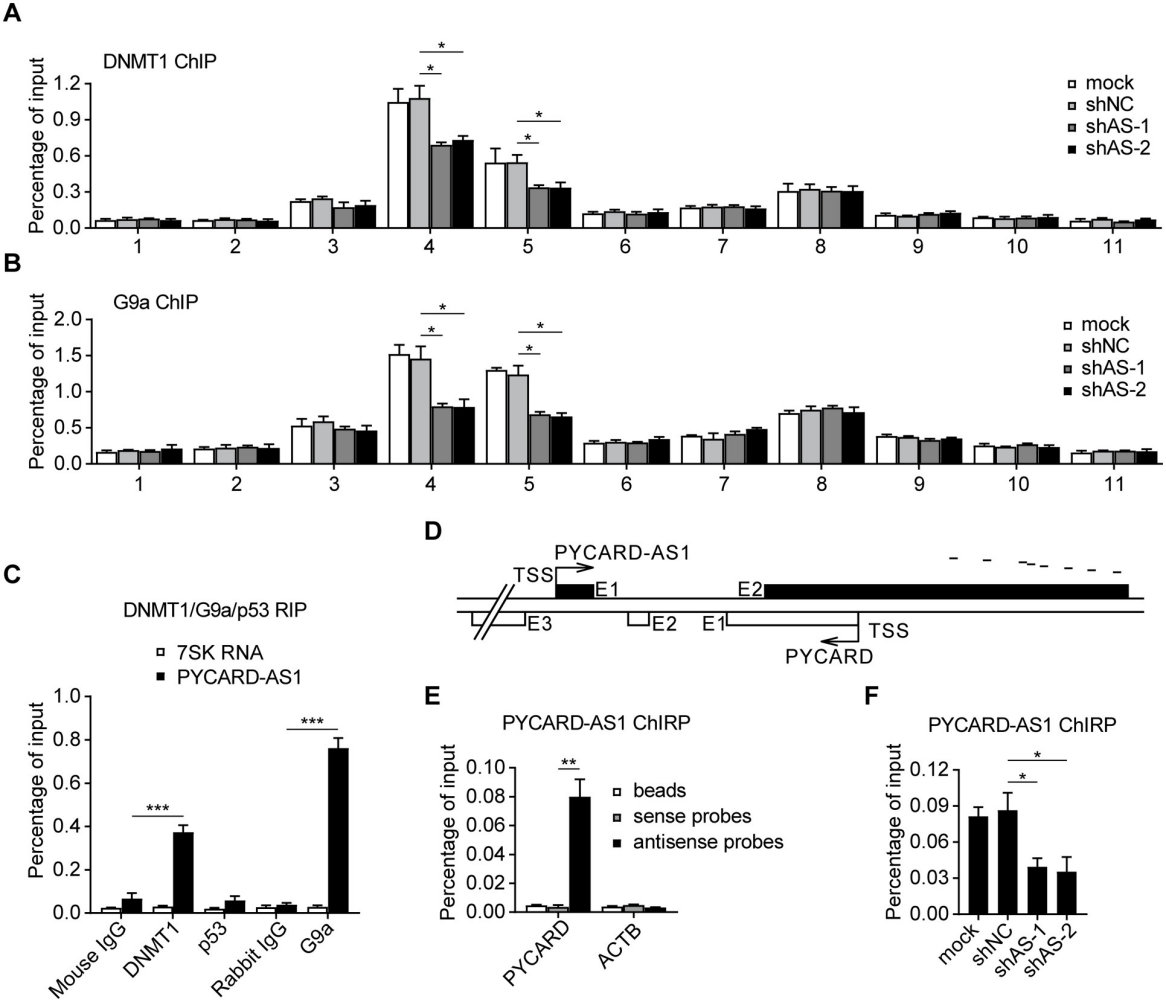
PYCARD-AS1 recruits DNMT1 and G9a to *PYCARD* promoter. **(A, B)** ChIP assays detecting the occupancy of DNMT1 (A) and G9a (B) on the segments shown in Fig 3A in SKBR3 cells with or without PYCARD-AS1 knockdown. **(C)** Native RIP detecting PYCARD-AS1 and 7SK RNA retrieved by DNMT1-, G9a-, or p53-specific antibody or by normal IgG. **(D)** Short lines schematically show location of the antisense probes used for ChIRP assays in (E, F). **(E)** ChIRP assay using oligos antisense to PYCARD-AS1 (antisense probes) detecting the *PYCARD* promoter DNA (segment 4 shown in Fig 3A), or the *ACTB* promoter DNA, associated with PYCARD-AS1 transcript in SKBR3 cells. Oligos reverse-complementary to the antisense probes (sense probes) and beads without probe addition (beads) were tested as controls. **(F)** ChIRP assay detecting the *PYCARD* promoter DNA associated with PYCARD-AS1 transcript in SKBR3 cells with or without PYCARD-AS1 knockdown. In (A, B, C, E and F), Data are presented as means ± SD from three independent experiments performed in triplicate; *p < 0.05; **p < 0.01; ***p < 0.001.

In addition to DNMT1, which had previously been identified [22], G9a, a histone methyltransferase responsible for H3K9 dimethylation, was also revealed to occupy the *PYCARD* promoter in SKBR3 cells (S3A Fig). *PYCARD* expression was induced by DNMT1 knockdown, G9a knockdown, or DNMT1 and G9a double-knockdown (S3B Fig). Moreover, PYCARD-AS1 knockdown was shown to cause simultaneous loss of the DNMT1 and G9a occupancy at the *PYCARD* promoter without influencing their expression levels (Fig 4A and 4B and S3C Fig). These findings indicate that *PYCARD* is under the control of DNMT1 and G9a, and that PYCARD-AS1 contributes to the recruitment of DNMT1 and G9a to the *PYCARD* promoter.

A native RIP assay using DNMT1- and G9a-specific antibodies retrieved a substantial amount of PYCARD-AS1 (Fig 4C), revealing a PYCARD-AS1 association with DNMT1 and G9a. Meanwhile, ChIRP assay using tiling oligos against PYCARD-AS1 (Fig 4D) specifically retrieved the *PYCARD* promoter DNA (Fig 4E), indicating that PYCARD-AS1 is also associated with the *PYCARD* locus. In accordance with the finding that shRNAs can efficiently knock down the nuclear-retained and chromatin-associated PYCARD-AS1 transcript (Fig 2C and 2D), PYCARD-AS1 knockdown was shown to decrease the *PYCARD* promoter DNA associated with PYCARD-AS1 transcript (Fig 4F). Together, the above results suggest that PYCARD-AS1 may recruit DNMT1 and G9a to *PYCARD* promoter via the intermolecular interactions.

### DNMT1 and G9a interact with the 3′ region of PYCARD-AS1

**Fig 5:**
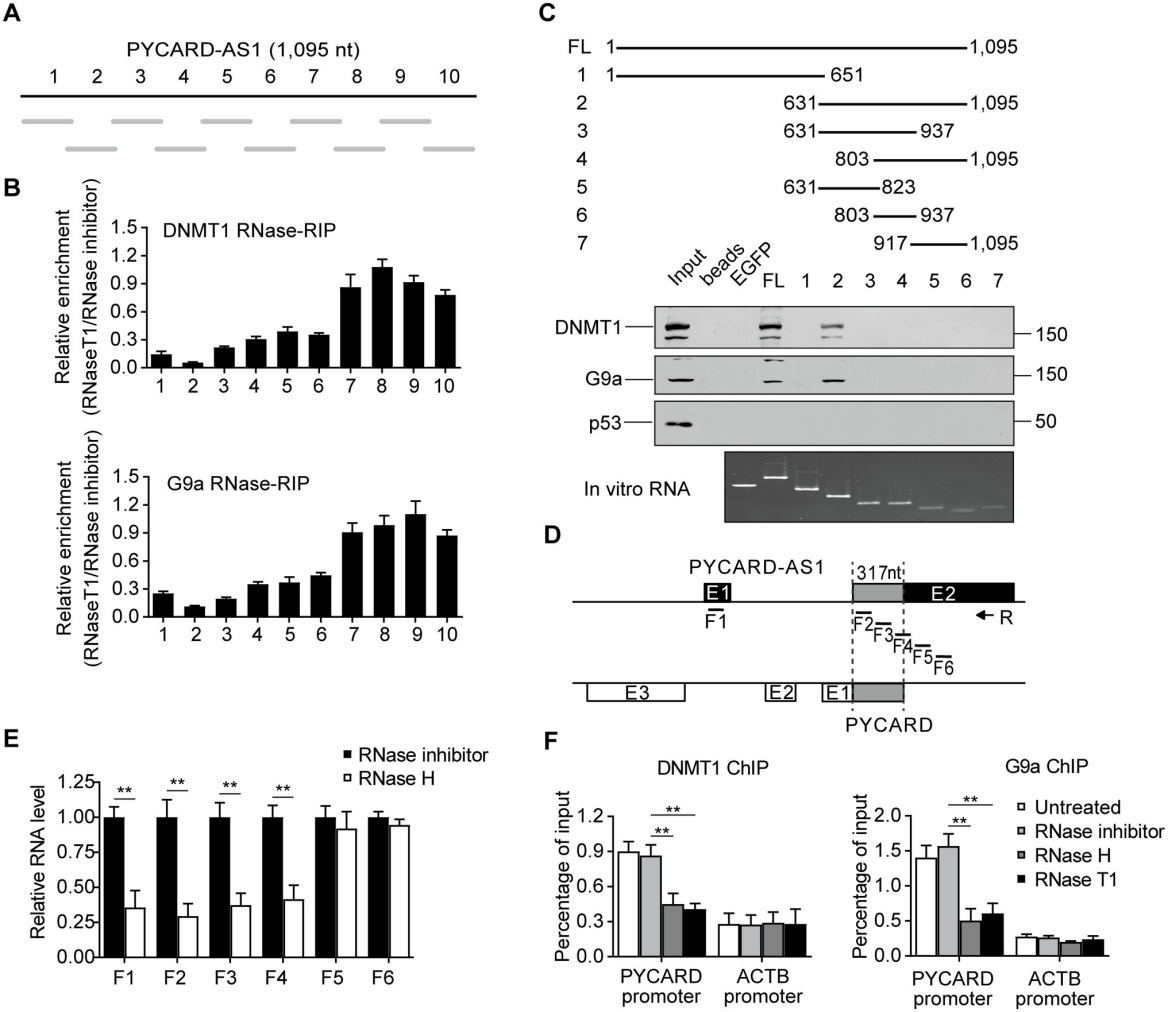
PYCARD-AS1 epigenetically silences *PYCARD* via two separated regions. **(A, B)** Schematic illustration of the PYCARD-AS1 segments (grey lines) (A), which were detected by an RIP-based mapping assay to identify the region associated with DNMT1 (B, upper) or G9a (B, lower). Results in (B) are presented relative to the RNase inhibitor control. **(C)** Biotin-labelled full-length PYCARD-AS1 and PYCARD-AS1 segments (shown schematically, upper) were used to pull down DNMT1, G9a, and p53 from SKBR3 cell lysates. EGFP mRNA and beads without RNA addition were tested as negative controls. The retrieved proteins were detected through immunoblotting (middle), and the used RNAs were visualized with ethidium bromide (lower). **(D)** Exon 2 of PYCARD-AS1 overlaps with the first exon of *PYCARD* by 317 nt. The short lines indicate the fragments that were detected by region-specific qRT-PCR in (E). **(E)** Region-specific qRT-PCR detecting PYCARD-AS1 in SKBR3 cells after the permeabilization treatment and the treatment with RNase inhibitor or RNase H. Reverse transcription reaction was initiated by PYCARD-AS1-specific reverse primer (R, shown schematically in (D)). **(F)** RNase-ChIP assays detecting the association of *PYCARD* promoter with DNMT1 (left) or G9a (right) in SKBR3 cells, which were permeabilized and treated with RNase inhibitor, RNase H or RNase T1. Untreated SKBR3 cells were also included. *ACTB* promoter DNA was tested as a control. In (B, E and F), data are presented as means ± SD from three independent experiments performed in triplicate; **p < 0.01.

We proceeded to elucidate the molecular mechanism whereby PYCARD-AS1 epigenetically regulates *PYCARD*. Although lncRNAs contain functionally redundant sequences, it is acknowledged that their core functionality generally depends on particular functional region [23]. To map the DNMT1- and G9a-interacting region within PYCARD-AS1, nuclear extract from SKBR3 cells was subjected to limited RNase T1 digestion, so that G residues protected by an RNA binding protein would remain preferentially uncleaved. After DNMT1 or G9a RIP, the enriched RNA fragments, which were associated with DNMT1 or G9a and protected from degradation, were identified by qRT-PCR analysis using primer sets that scanned the PYCARD-AS1 transcript in overlapping ~150 nt-long segments (Fig 5A). As shown in Fig 5B, the PYCARD-AS1 transcript from nt 631 to 1,095 was enriched by DNMT1- and G9a-specific antibodies after RNase T1 treatment, suggesting that the 3′ PYCARD-AS1 domain is the major region responsible for the DNMT1 and G9a interaction.

The above finding was confirmed by an RNA pull-down assay using an *in vitro*-generated biotinylated 3′ PYCARD-AS1 region, which was shown to bind DNMT1 and G9a with equal efficiency as full-length PYCARD-AS1 (Fig 5C). However, the effort to further narrow down and distinguish the DNMT1- and G9a-binding regions was failed because neither DNMT1 nor G9a could be retrieved by any of the biotinylated RNA segments derived from the 3′ PYCARD-AS1 region (Fig 5C). In addition to the consensus primary sequence, a proper secondary structure is also critical for the function of lncRNAs such as protein binding [24, 25]. Thus, further truncation of the 3′ PYCARD-AS1 region might have destroyed the necessary secondary structure and thus impair its property of association with DNMT1 and G9a. On the other hand, given that DNMT1 and G9a are reported to interact with each other directly [26], it could be speculated that DNMT1 and G9a form a unique complex prior to their binding to PYCARD-AS1. Consistently, our IP assay showed that RNase treatment didn′t destroy the DNMT1–G9a interaction (S4A Fig).

### PYCARD-AS1 localizes at the *PYCARD* locus via its 5′ region

Since there is a substantial degree of reverse complementarity between PYCARD-AS1 and *PYCARD* sequences, we tested whether the association of PYCARD-AS1 with the *PYCARD* locus, as demonstrated by ChIRP assay (Fig 4E and 4F), is mediated by direct base pairing, which results in formation of an RNA/DNA hybrid (i.e. R-loop). Total RNA extracted from RNase H- or RNase inhibitor-treated SKBR3 cells was subjected to region-specific qRT-PCR as well as primer walking assay. These analyses indicated that the region of potential RNA–DNA interaction is likely to be contained in a 317-nt fragment within exon 2 of PYCARD-AS1 (nt 576/892 with respect to the *PYCARD-AS1* TSS), which is complementary to a region of the first exon of *PYCARD* (nt 1/317 with respect to the *PYCARD* TSS), because this PYCARD-AS1 region is sensitive to the RNase H treatment, which degrades RNA in RNA/DNA hybrids (Fig 5D and 5E and S4B Fig).

We next established an RNase-ChIP assay to test whether R-loop formation contributes to the DNMT1/G9a occupancy at the *PYCARD* promoter, which was revealed to be PYCARD-AS1-dependent in SKBR3 cells (Fig 4A and 4B). RNase T1 treatment, which would degrade the single-stranded PYCARD-AS1 sequence lying outside the 3′ DNMT1/G9a-binding region, led to a separation of the 3′ PYCARD-AS1 region from the *PYCARD* DNA that abrogated the association between DNMT1/G9a complex and *PYCARD* promoter; if PYCARD-AS1 interacts with the *PYCARD* DNA sequence to recruit DNMT1 and G9a as expected, treatment with RNase H would result in the significant release of DNMT1 and G9a from the *PYCARD* promoter (Fig 5F). We next tested several other DNMT1- and G9a-targeted loci that include *KCNQ1* and *CDH1*, two genes characterized to also associate with the lncRNA KCNQ1OT1 or NEAT1 [27–30]. The results showed that the R-loop-dependent genomic recruitment of DNMT1 and G9a is not confined to the *PYCARD* locus (S4C and S4D Fig).

### PYCARD-AS1 interferes with *PYCARD* translation

**Fig 6:**
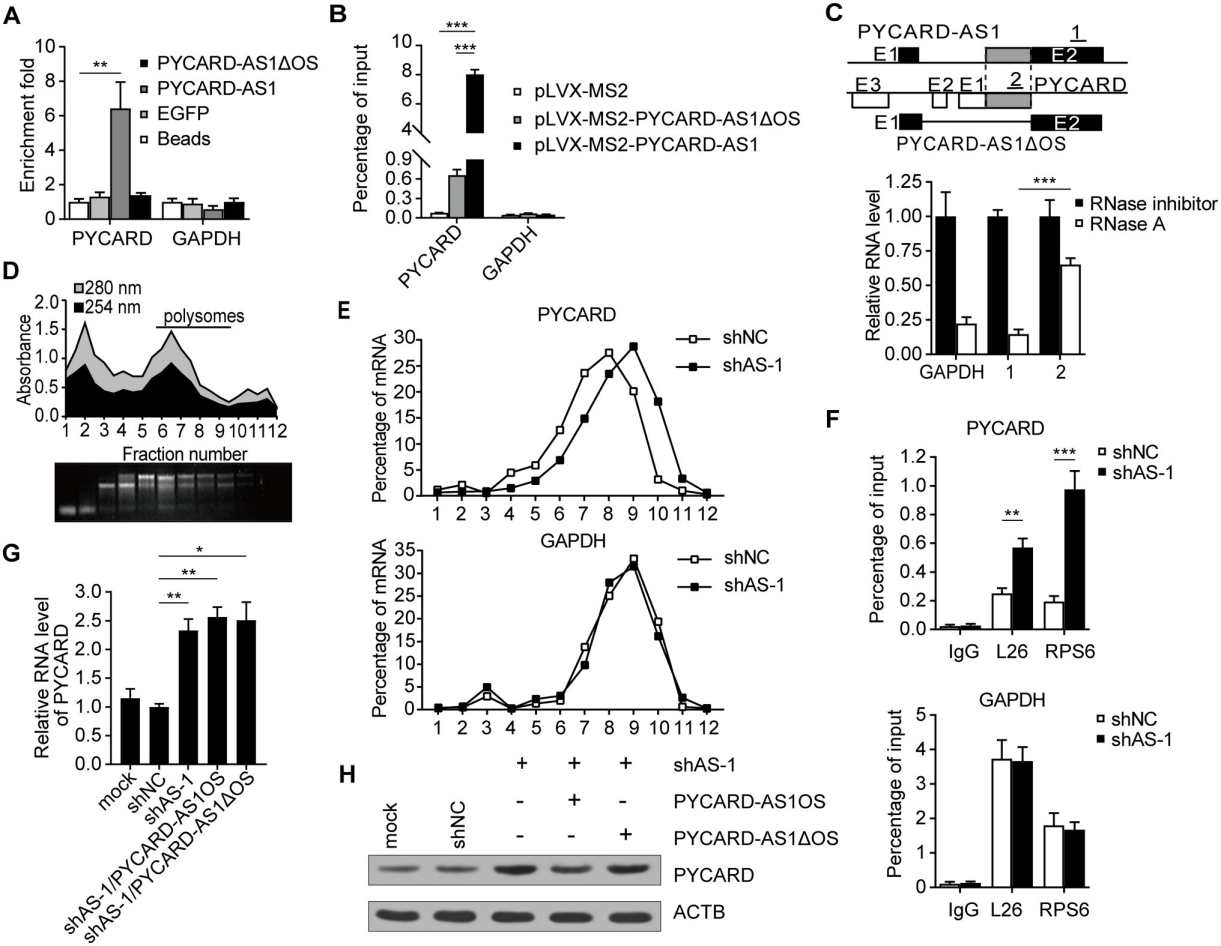
PYCARD-AS1 suppresses *PYCARD* translation. **(A)** The indicated biotin-labelled transcripts were used to pull down GAPDH or PYCARD mRNA from SKBR3 cell lysates. The retrieved mRNA was subjected to qRT-PCR analysis, and the data of retrieved mRNA are relative to the values for the control beads. The used PYCARD-AS1ΔOS transcript is shown schematically in (C, upper). **(B)** MS2-RIP assay detecting the PYCARD mRNA associated with PYCARD-AS1 or PYCARD-AS1ΔOS. GAPDH mRNA was detected as a negative RNA control. **(C)** RNase-A assay detecting the association between endogenous PYCARD-AS1 and PYCARD transcripts. RNA extracted from the RNase A-treated SKBR3 cell lysates was subjected to qRT-PCR analysis with primer sets that span the non-overlapping and overlapping regions (1 and 2, shown schematically), or with primers detecting GAPDH mRNA. **(D)** Schematic representation of the distribution of RNA, ribosomal subunits, ribosomes and polysomes along fractions of an increasing sucrose gradient (top to bottom fractions). RNA and protein abundances were determined by measuring the absorbance at 254 and 280 nm (upper); RNA samples extracted from gradient fractions were visualized with ethidium bromide (lower). **(E)** PYCARD mRNA (upper), but not GAPDH mRNA (lower), was increased in heavy polysomes by PYCARD-AS1 knockdown. The relative distribution of PYCARD or GAPDH mRNA was determined by qRT-PCR analysis of RNA in gradient fractions, and was presented as a percentage of the total RNA in the gradient. Data represent mean values from three independent experiments. **(F)** Native RIP detecting PYCARD (upper) or GAPDH (lower) mRNA retrieved by L26- or RPS6-specific antibody in SKBR3 cells with or without PYCARD-AS1 knockdown. **(G, H)** qRT-PCR (G) and immunoblotting (H) detecting the PYCARD expression level in response to PYCARD-AS1 knockdown with or without complementation with PYCARD-AS1OS or PYCARD-AS1ΔOS. In (A, B, C, F and G), data are presented as means ± SD from three independent experiments performed in triplicate; *p < 0.05; **p < 0.01; ***p < 0.001.

As PYCARD-AS1 and PYCARD mRNA constitute a pair of head-to-head overlapping transcripts, we next sought to determine whether they can interact with each other. Affinity pull-down assay showed that the *in vitro*-generated biotinylated full-length PYCARD-AS1 retrieved a substantial amount of PYCARD mRNA compared with the negative controls including beads alone or EGFP transcript (Fig 6A). In parallel, the deletion mutant of PYCARD-AS1 that lacks the overlapping region (PYCARD-AS1ΔOS, shown in Fig 6C, upper) was tested, and the result showed that deletion of the overlapping region led to an impaired association with PYCARD mRNA (Fig 6A). The interaction between PYCARD-AS1 and PYCARD transcripts was also confirmed within the cellular context by a MS2-RIP assay (Fig 6B); furthermore, an RNase-A assay indicated that the overlapping region of this pair of transcripts was resistant to RNase degradation (Fig 6C, lower). PYCARD-AS1 was revealed to exhibit a dual nuclear and cytoplasmic distribution (Fig 1E and 1F). Interestingly, by cellular component-specific RNase-A assays, the PYCARD-AS1–PYCARD interaction was detectable in both the nucleus and the cytoplasm (S5A Fig).

The interaction between PYCARD-AS1 and PYCARD transcripts implies a post-transcriptional regulation of *PYCARD* by PYCARD-AS1. lncRNAs have been involved in the stability control of their paired mRNAs [31–37]. However, it seems unlikely that PYCARD-AS1 operates through this strategy because its depletion didn′t change the half-life of PYCARD mRNA in SKBR3 cells, which were treated with α-amanitin in advance to block new RNA synthesis (S5B Fig). In addition, PYCARD-AS1 was demonstrated to have no effect on the subcellular distribution of PYCARD mRNA (S5C Fig).

We next tested whether PYCARD-AS1 gets connected to the *PYCARD* translation by monitoring the association of PYCARD mRNA with polysomes, the translational entity, in SKBR3 cells with or without PYCARD-AS1 knockdown. SKBR3 cell lysates fractionated through sucrose gradients were subjected to RNA isolation (Fig 6D), and the isolated RNA samples were used to measure the recruitment of PYCARD mRNA on polysomes by qRT-PCR. As shown in Fig 6E (upper), PYCARD-AS1 knockdown drove a shift of PYCARD mRNA toward heavier polysomes, in keeping with increased translation. As a negative control, the distribution of GAPDH mRNA in separation fractions did not change (Fig 6E, lower). Given that the overlapping region of this pair of sense–antisense transcripts is localized at their 5′ ends, we reasoned that PYCARD-AS1 can influence the ribosome assembly on PYCARD mRNA when they interact with each other. To test this, we established an RIP assay to determine whether PYCARD-AS1 knockdown can affect the ratio of ribosome-occupied PYCARD to total PYCARD mRNA, which was expected to eliminate the "contaminating" effect of PYCARD-AS1 knockdown on *PYCARD* transcription. The results clearly showed that occupancy of RPS6 and L26, the integral components of ribosomal subunits, on PYCARD mRNA was increased by PYCARD-AS1 knockdown (Fig 6F, upper). In parallel, PYCARD-AS1 knockdown was shown to have no effect on the RPS6 and L26 occupancy on GAPDH mRNA (Fig 6F, lower). To further address this issue, we performed a compensation experiment by infecting the PYCARD-AS1-knockdown SKBR3 cells, which had increased mRNA and protein levels of PYCARD (Fig 6G and 6H), with lentivirus expressing the 5′ overlapping region of PYCARD-AS1 (PYCARD-AS1OS) or the PYCARD-AS1ΔOS, which contains several point mutations that disrupt the shRNA target. Neither of the transcripts could compensate for the effect of PYCARD-AS1 knockdown on PYCARD mRNA level (Fig 6G). However, the PYCARD-AS1OS, but not the PYCARD-AS1ΔOS, has the ability to neutralize the *PYCARD* translation increased by PYCARD-AS1 knockdown (Fig 6H). Collectively, the results suggest that PYCARD-AS1 also suppresses the translation of *PYCARD* in addition to its transcription.

## Discussion

**Fig 7:**
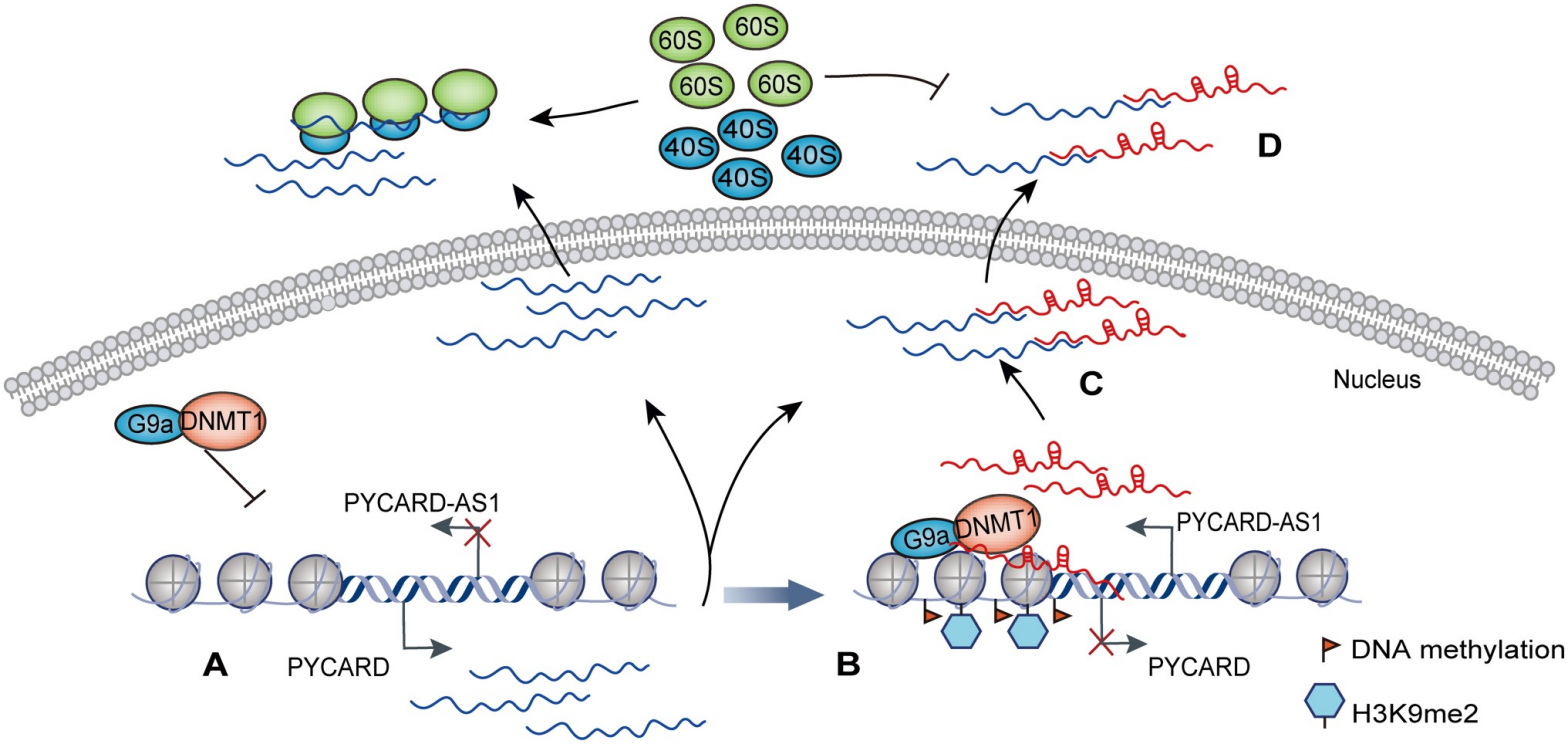
Proposed model of PYCARD-AS1-mediated *PYCARD* regulation. *PYCARD* transcription is activated in the case of PYCARD-AS1 silencing **(A)**. Upon transcribed from the opposite strand of the *PYCARD* gene, a fraction of PYCARD-AS1 transcripts localize at *PYCARD* promoter via the 5′ region, and interact with DNMT1 and G9a via the 3′ region, thereby recruiting the two chromatin-suppressor proteins to *PYCARD* locus **(B)**. On the other hand, some PYCARD-AS1 transcripts interact with PYCARD mRNAs via the 5′ sequence **(C)**, and then suppress *PYCARD* translation by interfering with the ribosome assembly **(D)**. This mechanism results in coordinated regulation of *PYCARD* at both epigenetic and translational levels by its antisense counterpart.

LncRNAs have been implicated in a large range of biological processes such as apoptosis, and their function is closely correlated with the cellular compartments where they are located. It is interesting that a sizable proportion of lncRNAs are discretely distributed in different cellular compartments [10]. PYCARD-AS1 reported here is such a "discrete" lncRNA, which exhibits a dual nuclear and cytoplasmic distribution, and our current study describes a model whereby the discretely distributed PYCARD-AS1 transcripts link different effector mechanisms to simultaneously operate in the different aspects of *PYCARD* regulation (Fig 7), contributing to the *PYCARD*-regulated apoptosis.

We first showed that PYCARD-AS1 can regulate *PYCARD* at the epigenetic level. Current evidence indicates that interaction with DNA methyltransferase or histone modifier is the major mechanism through which lncRNAs function in epigenetic regulation [2, 38]. Nevertheless, PYCARD-AS1 represents one of the few lncRNAs, which include KCNQ1OT1 and NEAT1 [27, 28, 30], identified to simultaneously modulate DNA methylation and histone modification at the loci of regulated genes. Biochemical interactions between DNA and histone methyltransferases were thought to provide a molecular explanation for the combinatorial pattern of DNA and histone modification in chromatin [26, 39, 40]. The current data further indicate that PYCARD-AS1 interacts with the DNMT1/G9a complex and aids in its recruitment to the *PYCARD* promoter. The core functionality of lncRNAs relies heavily on the cooperative action of their dispersed functional regions [23, 41]. In the case of PYCARD-AS1-mediated epigenetic regulation of *PYCARD*, PYCARD-AS1 interacts with DNMT1/G9a complex via its 3′ region, and localizes at the *PYCARD* promoter via its 5′ region, thereby facilitating the location-specific DNMT1/G9a recruitment. While the detailed mechanism underlying the lncRNA-mediated R-loop formation is not fully understood [42], our results showed that the R-loop structure formed by the 5′ PYCARD-AS1 region substantially contributes to the PYCARD-AS1-mediated *PYCARD* regulation. On the other hand, the interactions between lncRNA and DNMT1 are documented to have either positive or negative effect on the activity of DNMT1 [43–45]. The "guiding" lncRNAs, including PYCARD-AS1 reported here, can facilitate the DNMT1-mediated DNA methylation by recruiting DNMT1, whereas some other lncRNAs, such as ecCEBPA and Dali, were found to sequester the DNMT1 activity as they compete with the DNA substrate of DNMT1 for DNMT1 binding [46, 47].

PYCARD-AS1 also exerts inhibitory effect on *PYCARD* translation. Recent studies have shown that through binding mRNAs, lncRNAs, especially antisense lncRNAs, can repress or promote mRNA translation [35, 48–50]. The distinct effects may depend on specific binding sequences and embedded elements in lncRNAs. In the case of PYCARD-AS1-mediated repression of *PYCARD* translation, PYCARD-AS1 activity depends on the 5′ overlapping sequence, which interferes with the ribosome assembly on *PYCARD* mRNA. The feature of 5′ overlapping sequence is shared by many other natural antisense transcripts. Nevertheless, the antisense Uchli RNA was reported to exert an opposite effect on the translation of its sense counterpart due to the presence of an embedded inverted SINEB2 element, which facilitates the ribosome binding to mRNA [49]. The paired antisense lncRNA may also affect certain other steps in protein translation. For instance, a binding of the PXN mRNA by its antisense lncRNA PXN-AS1-S can reduce PXN protein synthesis by inhibiting translational elongation [35]. In addition, a recent study reported that through competitive RNA–RNA interaction, an lncRNA is able to attenuate the activity of its paired antisense lncRNA in repression of mRNA translation [50], thereby constituting a finely tuned lncRNA/antisense lncRNA/mRNA translational regulatory axis. The cluster of natural antisense transcripts comprises a surprisingly large fraction of lncRNAs [51]; moreover, the lncRNA–mRNA gene pairs are prevalent in mammalian genomes [52]. Thus, effect on mRNA translation might be a common effector mechanism employed by lncRNAs, especially antisense lncRNAs, to function in biological processes.

Taken together, this study provides an example of how an lncRNA works at different cellular compartments to regulate a specific target gene at multiple levels, contributing to the regulation of apoptosis. The feature of discrete distribution in different cellular compartments is not exclusively exhibited by PYCARD-AS1; instead, it is extensively shared by other lncRNAs [10]. We propose that, as with PYCARD-AS1, many other discretely distributed lncRNAs should also be multifunctional within the cell, and that elucidating their different functionalities at distinct distribution sites will greatly broaden our knowledge of lncRNA biology and provide new insights into their physiological and pathological roles in depth.

## Materials and methods

### Plasmid constructions

PYCARD-AS1 cDNA was synthesized from SKBR3 cells by RT-PCR. For the test of protein-coding potentiality of PYCARD-AS1, the EGFP-coding sequence was inserted into the 3′ end of the putative PYCARD-AS1 ORF, and the fusion gene PYCARD-AS1-EGFP was cloned into the restriction sites *Nhe* I and *Xho* I of plasmid pcDNA3.1 (Invitrogen). For lentivirus-mediated RNA interference, complementary sense and antisense oligonucleotides encoding short hairpin RNAs (shRNAs) targeting PYCARD-AS1, PYCARD, DNMT1 and G9a transcripts were synthesized, annealed and cloned into the *Age* I and *Eco*R I sites of plasmid pLKO.1 (Addgene). For compensation experiment, cDNA corresponding to the PYCARD-AS1OS or PYCARD-AS1ΔOS was PCR-amplified from the PYCARD-AS1 cDNA and cloned into the *Xho* I and *Eco*R I sites of plasmid pLVX (BD Clontech). For MS2-RIP experiment, the MS2-6× fragment was synthesized and fused to the 5′ end of PYCARD-AS1 or PYCARD-AS1ΔOS cDNA, and the resulting fusion constructs (MS2-PYCARD-AS1 and MS2-PYCARD-AS1ΔOS) were cloned into the *Xho* I and *Eco*R I sites of plasmid pcDNA3.1; the sequence encoding MS2 coat protein was PCR-amplified from plasmid pMS2-GFP (Addgene) and fused to the 3′ end of FLAG tag-encoding sequence, and the resulting FLAG-MS2 construct was cloned into the *Hin*d III and *Xho* I sites of plasmid pcDNA3.1. The primers and oligonucleotides used for plasmid construction are shown in S2 Table.

### Cell culture

HEK293T, SKBR3, T47D, MDA-MB-231 and MCF7 cells were obtained from the ATCC and cultured in DMEM, MEM or RPMI1640 supplemented with 10% FBS in a 5% CO_2_ incubator at 37°C. To block cellular transcription by Pol II, SKBR3 cells in culture media were treated with 50 μM α-amanitin (Sigma-Aldrich). For the apoptosis analysis, SKBR3 cells were treated with 5 nM paclitaxel (Sigma-Aldrich) for 72 h. Plasmid transfections were performed using Lipofectamine 2000 (Invitrogen). For lentivirus infection, shRNA-encoding pLKO.1, or pLVX encoding specific PYCARD-AS1OS or PYCARD-AS1ΔOS, was co-transfected with psPAX2 and pMD2.G plasmids (Addgene) into HEK293 cells; the infectious lentivirus was harvested 2 days post-transfection, filtered through 0.45-μm PVDF filters and transduced into SKBR3, T47D, MDA-MB-231 or MCF7 cells. After lentivirus infection, the resulting cell population, but not the isolated single clones, were used for subsequent assays to avoid clone-specific effects.

### 5′- and 3′-RACE

The full-length PYCARD-AS1 was obtained using 5′- and 3′-RACE System for Rapid Amplification of cDNA Ends (Invitrogen) in accordance with the manufacturer′s instructions. RACE PCR products were separated on a 1.5% agarose gel. Gel products were extracted with a Gel Extraction kit (Foregene), cloned into pMD18-T vector and sequenced bidirectionally using M13 forward and reverse primers. The primers used in the RACE experiments are shown in S2 Table.

### RNA FISH

RNA FISH was conducted using QuantiGene ViewRNA ISH Cell Assay Kit (Invitrogen) in accordance with the manufacturer′s instructions. In brief, SKBR3 cells cultured on cover slips were fixed, permeabilized and digested by protease to allow target accessibility. A probe set specific for PYCARD-AS1 (designed and supplied by Invitrogen) was added to the cells and hybridization was performed at 40°C for 3 h. After a series of signal amplification with Pre-Amplifier Mix, Amplifier Mix and Label Probe Mix supplied in the kit, cells were counterstained with DAPI and then detected using a fluorescent microscope (Leica).

### Cellular fractionation

A total of 1 × 10^7^ cells were washed twice in cold PBS and then incubated in hypotonic buffer (50 mM HEPES, pH 7.5, 10 mM KCl, 350 mM sucrose, 1 mM EDTA, 1 mM DTT and 0.1% Triton X-100) on ice for 10 min. After 5 min of centrifugation at 2,000 *g*, the supernatant was collected as the cytoplasmic fraction, and after additional washing, the remainder was considered as nuclear pellets, which could be resuspended in lysis buffer (10 mM HEPES, pH 7.0, 100 mM KCl, 5 mM MgCl_2_, 0.5% NP-40, 10 μM DTT and 1 mM PMSF) to prepare the nuclear lysate. To isolate the chromatin-enriched RNA, the chromatin pellets, as well as the soluble nucleoplasm, was prepared from the nuclear extract as described [53].

### Gene expression analysis

RNA samples were prepared from whole cell lysate or specific subcellular fractions using TRIzol reagent (Invitrogen). RNA levels for a specific gene were measured by qRT-PCR (starting with 50–100 ng RNA sample per reaction) using Real-Time PCR Easy (Foregene), in accordance with the manufacturer′s instructions. The qRT-PCR data were normalized to ACTB mRNA, 18S rRNA, mitochondrial-retained 12S rRNA, nuclear-localized U1 snRNA, or chromatin-associated XIST RNA, or presented as a percentage of the total amount of detected transcripts. The primers used in the qRT-PCR are shown in S2 Table.

For global gene expression analysis, 15 μg of biotinylated cDNA synthesized from total RNA was hybridized to the Affymetrix GeneChip^®^ PrimeView^™^ Human Gene Expression Array at 45°C for 16 h with Affymetrix GeneChip Hybridization Oven 640. After washing and staining with Affymetrix Fluidics Station 450, GeneChips were scanned by the Affymetrix GeneChip Command Console installed in the GeneChip Scanner 3000 7G. Hybridization data were analyzed with the Robust Multichip Analysis (RMA) algorithm using the default Affymetrix settings. Values are presented as log2 RMA signal intensity.

### Apoptosis assay

A total of 5 × 10^5^ cells treated with paclitaxel were harvested and stained with Alexa Fluor^®^ 488 Annexin V/Dead Cell Apoptosis kit (Invitrogen), in accordance with the manufacturer′s instructions. Flow cytometry analysis was carried out using an Accuri C6 flow cytometer (BD Biosciences).

### Bisulfite sequencing

Genomic DNA was extracted with the Genomic DNA Isolation kit (Foregene) and the bisulfite conversion reaction was performed using CpGenome Turbo Bisulfite Modification kit (Millipore), in accordance with the manufacturer′s instructions. PCR amplification of bisulfite-treated DNA was carried out with PCR Easy (Foregene). The amplified products were cloned and sequenced. The primers used in the PCR amplification are shown in S2 Table.

### Antibodies (Abs)

The Abs used for immunoblotting were rabbit anti-PYCARD Ab (13833, Cell Signaling), mouse anti-actin Ab (sc-130301, Santa Cruz Biotechnology), mouse anti-DNMT1 Ab (ab13537, Abcam) and goat anti-G9a Ab (sc-22879, Santa Cruz Biotechnology). The Abs used for IP analysis were mouse anti-DNMT1 Ab (ab13537, Abcam) and normal mouse IgG (sc-2025, Santa Cruz Biotechnology). The Abs used for ChIP analysis were rabbit anti-H3K9me2 Ab (4658, Cell Signaling), rabbit anti-H3K27me3 Ab(9733, Cell Signaling), rabbit anti-RNA polymerase II CTD repeat YSPTSPS (S5P Pol II) Ab (ab5131, Abcam), rabbit anti-RNA polymerase II CTD repeat YSPTSPS (S2P Pol II) Ab (ab5095, Abcam), mouse anti-RNA pol II Ab (39097, Active Motif), mouse anti-DNMT1 Ab (ab13537, Abcam), rabbit anti-G9a Ab (ab40542, Abcam), normal mouse IgG (sc-2025, Santa Cruz Biotechnology) and normal rabbit IgG (sc-2027, Santa Cruz Biotechnology). The Abs used for RIP analysis were mouse anti-DNMT1 Ab (ab13537, Abcam), rabbit anti-G9a Ab (ab40542, Abcam), mouse anti-p53 Ab (P6874, Sigma-Aldrich), rabbit anti-RPS6 Ab (ab70227, Abcam), rabbit anti-L26 Ab (ab59567, Abcam), normal mouse IgG (sc-2025, Santa Cruz Biotechnology) and normal rabbit IgG (sc-2027, Santa Cruz Biotechnology). The Ab used for nuclear run-on assay was anti-BrdU Ab (ab1893, Abcam).

### Immunoblotting and immunoprecipitation

A total of 5 × 10^6^ cells were washed twice in cold PBS and pelleted. The pellet was resuspended in lysis buffer (10 mM HEPES, pH 7.0, 100 mM KCl, 5 mM MgCl_2_, 0.5% NP-40, 10 μM DTT and 1 mM PMSF), incubated on ice with frequent vortexing for 15 min and then the lysate was obtained by centrifugation at 12,000 *g* for 10 min. Protein concentrations of the extracts were measured by the bicinchoninic acid assay (Pierce). Forty micrograms of the protein was used for immunoprecipitation, or was fractionated by SDS-PAGE, transferred onto PVDF membranes and then blotted.

For immunoprecipitation assays, protein samples were incubated with a specific antibody or control IgG overnight at 4°C. Subsequently, the samples were incubated with 50 μl of protein A agarose beads (Invitrogen) for 4 h at 4°C and then washed three times in washing buffer (50 mM Tris-HCl, pH 7.5, 150 mM NaCl, 1 mM MgCl_2_ and 0.5% NP-40). Finally, protein complexes were eluted by SDS buffer (120 mM Tris-HCl, pH 6.8, 20% glycerol and 4% SDS) and then detected by immunoblotting.

### Native RIP assay

For native RIP assays, RNase OUT (50 U/ml, Invitrogen) and a protease inhibitor cocktail (Roche) were added to the lysis buffer, and Ribonucleoside Vanadyl Complex (10 mM, NEB) was added to the washing buffer (the buffer mentioned here is the same as that used in immunoblotting and immunoprecipitation). Following the addition of antibody to the lysate, samples were incubated with 50 μl of protein A agarose bead (Invitrogen) for 4 h at 4°C and then washed three times in washing buffer. The beads were resuspended and treated with proteinase K at 45°C for 45 min. Coprecipitated RNAs were extracted using TRIzol reagent, ethanol-precipitated with Glycoblue (Invitrogen) as a carrier and then detected by qRT-PCR. The data of retrieved RNAs are presented as a percentage of the amount input.

For RIP-based mapping assays, lysates were first mixed with RNase T1 (1 U/ml, Thermo Fisher Scientific), after which standard native RIP assays were performed using an antibody against DNMT1 or G9a. Following extraction of the coprecipitated RNA, the PYCARD-AS1 segments bound by DNMT1 and G9a, and hence protected from RNase T1 digestion and immunoprecipitated, were identified by qRT-PCR analysis using primer sets that scanned the PYCARD-AS1 transcript at ~150-nt-long, overlapping intervals (S2 Table).

For MS2-RIP, pcDNA3.1-MS2-PYCARD-AS1, or pcDNA3.1-MS2-PYCARD-AS1ΔOS, was co-transfected with pcDNA3.1-FLAG-MS2 into SKBR3 cells. After 48 h, cells were subjected to RIP assay with Anti-FLAG M2 Magnetic beads (Sigma-Aldrich) in accordance with the manufacturer′s instructions.

### Nuclear run-on assay

A total of 1.5 × 10^7^ cells were washed with cold PBS and harvested in cold douncing buffer (5 mM MgCl_2_, 0.5% NP-40, 10% glycerol, and 50 mM Tris-HCl, pH 7.4). After 10 min of incubation on ice, cells were disrupted with 30 strokes of a Dounce homogenizer, centrifuged at 3,300 *g* for 5 min and washed four times with 1 ml cold douncing buffer. Microscopic analysis indicated that samples contained intact nuclei, cellular debris and some intact cells. The crude nuclei were then resuspended in 100 μl nuclear run-on buffer (5 mM MgCl_2_, 150 mM KCl, 0.1% sarbyl, 10 mM DTT and 50 mM Tris-HCl, pH 7.4), and were mixed with 1 μl each of 10 mM ATP, GTP, CTP, 1 μl 10 mM BrUTP (Sigma-Aldrich) and 1 μl RNase inhibitor (Thermo Fisher Scientific). Reaction mixtures were pre-incubated on ice for 30 min, then at 28°C for 5 min. The RNA was isolated by TRIzol reagent (Invitrogen), and DNA was removed by DNase I (Promega) treatment. Nascent transcripts were immunoprecipitated with anti-BrdU antibody (Abcam) and subjected to qRT-PCR assays with primers listed in S2 Table.

### ChIP and RNase-ChIP

ChIP analyses were performed as described [7]. For RNase-ChIP assays, 1 × 10^6^ cells were collected by centrifugation, permeabilized in 1 ml of PBST (PBS containing 0.05% Tween 20), and treated with 1,000 U/ml RNase T1 (Thermo Fisher Scientific), 1,000 U/ml RNase H (Thermo Fisher Scientific) or 1,000 U/ml RNase inhibitor (Thermo Fisher Scientific) for 4 h at 25°C. The following procedures were carried out in accordance with the standard ChIP protocol. The genomic DNA in the precipitate was detected by qPCR using the primers shown in S2 Table, and the DNA precipitated by each antibody, including IgG, is presented as a percentage of the amount input.

### RNase-H assay

The collected cells were subjected to permeabilization treatment and then treated with RNase H or RNase inhibitor as described in RNase-ChIP assay. RNA was extracted from the treated cells and subjected to region-specific qRT-PCR as well as primer walking assay using primers listed in S2 Table to test the abundance of specific PYCARD-AS1 regions.

### ChIRP assay

Cells were cross-linked by 1% glutaraldehyde at room temperature for 10 min, followed by three washes in cold PBS. After being snap-frozen by liquid nitrogen and stored at −80°C, cross-linked cells were resuspended in nuclear lysis buffer (10 mM EDTA, 1% SDS and 50 mM Tris-HCl, pH 7.5) supplemented with a protease inhibitor cocktail (Roche), and sonicated until DNA was in the size range of 100~500 bp. Cell lysates and a set of biotin-labelled antisense probes (20 nt in length) were then incubated at 37°C for 4h, with the corresponding sense probes being included as a control. Streptavidin-coupled Dynabeads (Invitrogen) were added to pull down the probes. After washing, the retrieved DNA was isolated using the ChIP DNA Clean & Concentrator kit (Zymo Research) and subjected to qPCR analysis. The probes used for ChIRP are shown in S2 Table.

### RNA pull-down

To synthesize biotin-labelled transcripts, PCR fragments were prepared using forward primers harboring the T7 RNA polymerase promoter. Following purification of the PCR products, biotinylated transcripts were synthesized using MaxiScript T7 kit (Ambion). Biotinylated RNA was heated to 85°C for 2 min, placed on ice for 2 min, supplied with RNA structure buffer (0.1 M KCl, 10 mM MgCl_2_ and 10 mM Tris-HCl, pH 7.0) and then incubated at room temperature for 20 min. Cell lysates were incubated with 10 pmol of biotinylated transcripts for 3 h at 25°C. Complexes were isolated with streptavidin-coupled Dynabeads (Invitrogen). The retrieved protein and RNA were detected by immunoblotting and qRT-PCR, respectively.

### RNase-A assay

RNase-A assay was performed as described [31]. Briefly, cell lysates were treated with 20 ng/ml RNase A (Thermo Fisher Scientific) at 37°C for 30 min. RNA was extracted from the resultant sample, and subjected to qRT-PCR using primers shown in S2 Table to detect the association between PYCARD-AS1 and PYCARD transcripts.

### Polysome analysis

Polysome analysis was performed as described [48]. A total of 5 × 10^6^ cells were preincubated with 100 mg/ml cycloheximide (Sigma-Aldrich) for 15 min. Cytoplasmic lysates were prepared and then fractionated by ultracentrifugation through 15%–50% linear sucrose gradients. Twelve fractions were collected, and RNA extracted from each fraction was subjected to qRT-PCR detection for specific transcript.

### Statistical analysis

Student′s *t*-test was performed to compare the differences between experimental groups relative to their paired controls. The data were presented as the mean ± SD and *p*-values of < 0.05 or below were considered statistically significant.

## Supporting information

S1 Fig PYCARD-AS1 is a Pol II-transcribed noncoding transcript. **(A)** PYCARD-AS1 is poly(A)-tailed. Total RNA extracted from SKBR3 cells was reverse-transcribed using Oligo-dT or random hexamers (RP6). GAPDH mRNA served as a control polyadenylated transcript, and pre-tRNA^tyr^ served as a control non-polyadenylated transcript. **(B)** PYCARD-AS1 transcription is sensitive to α-amanitin. SKBR3 cells were treated with α-amanitin at a concentration of either 0 or 50 μM for 8 h. Total RNA was prepared from these cells, and the levels of the indicated transcripts were determined by RT-PCR. Increasing amounts of cDNA template from the untreated cells (lanes 1–3) were used for PCR to test whether the PCR amplification occurs quantitatively. 45S pre-rRNA, pre-tRNA^tyr^ and ACTB were used as controls. **(C)** PYCARD-AS1 was predicted to be a noncoding transcript. The RNA sequences of PYCARD-AS1, PYCARD, GAPDH and HOTAIR were put into the CPC program, with HOTAIR serving as a control noncoding transcript, while PYCARD and GAPDH served as control coding transcripts. Scores above 0 suggest coding potential, whereas scores below 0 represent no coding potential. **(D, E)** The ORF analysis of PYCARD-AS1 sequence by UniProt (D) and the diagram of fusion gene PYCARD-AS1-EGFP inserted in pcDNA3.1 plasmid. **(F)** Phase contrast or fluorescence microscopy of SKBR3 cells that had been transfected with the indicated plasmid (scale bars, 100 μm). **(G)** qRT-PCR assays detecting the distribution of the indicated transcripts in chromatin and nucleoplasm extract from SKBR3 cells. XIST, a canonically chromatin-associated lncRNA, and the protein-coding GAPDH mRNA, were assessed as controls to confirm the findings of our chromatin fractionation. The qRT-PCR data, represented as a percentage of the detected transcripts in nuclear fraction, are presented as means ± SD from three independent experiments performed in triplicate.
(TIF)

S2 Fig PYCARD-AS1 is a negative regulator of *PYCARD*. **(A)** qRT-PCR measuring expression level of *PYCARD-AS1* in the indicated breast cancer lines relative to the level in SKBR3 cells. 18S rRNA was used as an internal control to normalize the amount of total RNA in the samples. **(B)** The replicate blots subjected to the densitometric analysis in Fig 2H. **(C)** qRT-PCR detecting the effect of PYCARD knockdown on PYCARD-AS1 level in SKBR3 cells. **(D)** qRT-PCR detecting the effect of PYCARD-AS1 knockdown on the mRNA levels of *FUS*, *TRIM72* and *PYDC1* in SKBR3 cells. **(E–G)** qRT-PCR (left) and immunoblotting (right) detecting the effect of PYCARD-AS1 knockdown on *PYCARD* expression in MCF7 (E), MDA-MB-231 (F) and T47D (G) cells. **(H)** qRT-PCR detecting the abundance of PYCARD in SKBR3 cells after PYCARD-AS1 knockdown and simultaneous PYCARD knockdown. **(I)** Representative plots of apoptosis of the indicated SKBR3 cells with or without paclitaxel treatment. **(J)** qRT-PCR of a representative panel of PYCARD-AS1- and PYCARD-regulated genes in the indicated SKBR3 cells. In this figure, the qRT-PCR data are presented as means ± SD from three independent experiments performed in triplicate; for immunoblotting, signals from three independent assays were subjected to densitometric analysis, and the data are presented as means ± SD; * *p* < 0.05; ** *p* < 0.01; *** *p* < 0.001.
(TIF)

S3 Fig DNMT1 and G9a regulate *PYCARD*. **(A)** ChIP assays detecting association of DNMT1 (upper) and G9a (lower) with the segments shown in Fig 3A in SKBR3 cells. **(B)** qRT-PCR detecting the levels of DNMT1, G9a, PYCARD and PYCARD-AS1 in the indicated SKBR3 cells. **(C)** qRT-PCR detecting the levels of DNMT1 and G9a in SKBR3 cells with or without PYCARD-AS1 knockdown. Data in this figure are presented as mean ± SD from three independent experiments performed in triplicate; **p < 0.01.
(TIF)

S4 Fig DNMT1 and G9a regulate *PYCARD* via PYCARD-AS1. **(A)** The interaction between DNMT1 and G9a confirmed by DNMT1 IP followed by immunoblotting. The interaction was not abolished by DNase I or RNase A treatment. **(B)** Semi-quantitative RT-PCR detecting the PYCARD-AS1 region associated with the *PYCARD* locus in SKBR3 cells after the permeabilization treatment and the treatment with an RNase H or RNase inhibitor. The reverse transcription reaction was initiated by a PYCARD-AS1-specific reverse primer (R, shown schematically), which was paired with each forward walking primers (F1–F6, shown schematically) in the subsequent PCR amplification. **(C, D)** RNase-ChIP assays detecting the association of DNMT1 (C) or G9a (D) with the indicated gene promoters. SKBR3 cells were permeabilized and treated with an RNase inhibitor, RNase H or RNase T1, in advance. Untreated SKBR3 cells were also included. In (C and D), data are presented as mean ± SD from three independent experiments performed in triplicate; * *p* < 0.05.
(TIF)

S5 Fig Interaction between the PYCARD-AS1 and PYCARD transcripts. **(A)** RNase-A assay detecting the interaction between PYCARD-AS1 and PYCARD transcripts in the nucleus (left) and cytoplasm (right). Nuclear and cytoplasmic lysates were prepared from SKBR3 cells, and the lysates were subjected to RNase-A treatment, RNA extraction and qRT-PCR analysis to detect the non-overlapping and overlapping regions (1 and 2) described in Fig 6B. **(B)** The stability of PYCARD (left) and GAPDH (right) mRNAs over time was measured by qRT-PCR relative to the start time point after blocking new RNA synthesis with α-amanitin in SKBR3 cells with or without PYCARD-AS1 knockdown and normalized to 18S rRNA. **(C)** qRT-PCR analysis following nuclear/cytoplasmic fractionation detecting the distribution of PYCARD (left) and GAPDH (right) mRNAs in SKBR3 cells with or without PYCARD-AS1 knockdown. Data in this figure are presented as means ± SD from three independent experiments performed in triplicate; ** *p* < 0.01; *** *p* < 0.001.
(TIF)

S1 Table List of genes that are regulated by PYCARD-AS1 knockdown and PYCARD-AS1/PYCARD double-knockdown. (DOCX)

S2 Table Sequences of primers and oligos used in this study. (DOCX)

S1 Data Excel file containing the underlying numerical data for Fig 1F. (XLSX)

S2 Data Excel file containing the underlying numerical data for Figs 2A, 2B, 2C, 2D, 2E, 2F, 2G, 2H, 1I and 2K. (XLSX)

S3 Data Excel file containing the underlying numerical data for Fig 3B, 3C and 3D. (XLSX)

S4 Data Excel file containing the underlying numerical data for Fig 4A, 4B, 4C, 4E and 4F. (XLSX)

S5 Data Excel file containing the underlying numerical data for Fig 5B, 5E and 5F. (XLSX)

S6 Data Excel file containing the underlying numerical data for Fig 6A, 6B, 6C, 6E, 6F and 6G. (XLSX)

S7 Data Excel file containing the underlying numerical data for S1G Fig. (XLSX)

S8 Data Excel file containing the underlying numerical data for S2A, S2C, S2D, S2E, S2F, S2G, S2H and S2J Fig. (XLSX)

S9 Data Excel file containing the underlying numerical data for S3A, S3B and S3C Fig. (XLSX)

S10 Data Excel file containing the underlying numerical data for S4C and S4D Fig. (XLSX)

S11 Data Excel file containing the underlying numerical data for S5A, S5B and S5C Fig. (XLSX)

## References

[1] Guttman M, Amit I, Garber M, French C, Lin MF, Feldser D, et al Chromatin signature reveals over a thousand highly conserved large non-coding RNAs in mammals. Nature. 2009; 458: 223–227. 10.1038/nature07672 19182780 PMC2754849

[2] Rinn JL, Chang HY. Genome regulation by long noncoding RNAs. Annu Rev Biochem. 2012; 81: 145–166. 10.1146/annurev-biochem-051410-092902 22663078 PMC3858397

[3] Batista PJ, Chang HY. Long noncoding RNAs: cellular address codes in development and disease. Cell. 2013; 152: 1298–1307. 10.1016/j.cell.2013.02.012 23498938 PMC3651923

[4] Ulitsky I, Bartel DP. lincRNAs: genomics, evolution, and mechanisms. Cell. 2013; 154: 26–46. 10.1016/j.cell.2013.06.020 23827673 PMC3924787

[5] Chen LL. Linking long noncoding RNA localization and function. Trends Biochem Sci. 2016; 41: 761–772. 10.1016/j.tibs.2016.07.003 27499234

[6] Wang KC, Chang HY. Molecular mechanisms of long noncoding RNAs. Mol Cell. 2011; 43: 904–914. 10.1016/j.molcel.2011.08.018 21925379 PMC3199020

[7] Li L, Feng T, Lian Y, Zhang G, Garen A, Song X. Role of human noncoding RNAs in the control of tumorigenesis. Proc Natl Acad Sci USA. 2009; 106: 12956–12961. 10.1073/pnas.0906005106 19625619 PMC2722347

[8] Wang G, Cui Y, Zhang G, Garen A, Song X. Regulation of proto-oncogene transcription, cell proliferation, and tumorigenesis in mice by PSF protein and a VL30 noncoding RNA. Proc Natl Acad Sci USA. 2009; 106: 16794–16798. 10.1073/pnas.0909022106 19805375 PMC2757814

[9] Lan Y, Xiao X, He Z, Luo Y, Wu C, Li L, et al Long noncoding RNA OCC-1 suppresses cell growth through destabilizing HuR protein in colorectal cancer. Nucleic Acids Res. 2018; 46: 5809–5821. 10.1093/nar/gky214 29931370 PMC6009600

[10] Cabili MN, Dunagin MC, McClanahan PD, Biaesch A, Padovan-Merhar O, Regev A, et al Localization and abundance analysis of human lncRNAs at single-cell and single-molecule resolution. Genome Biol. 2015; 16: 20 10.1186/s13059-015-0586-4 25630241 PMC4369099

[11] McConnell BB, Vertino PM. Activation of a caspases-9-mediated apoptotic pathway by subcellular redistribution of the novel caspase recruitment domain protein TMS1. Cancer Res. 2000; 60: 6243–6247. 11103777

[12] Ohtsuka T, Ryu H, Minamishima YA, Macip S, Sagara J, Nakayama KI, et al ASC is a BAX adaptor and regulates the p53-Bax mitochondrial apoptosis pathway. Nat Cell Biol. 2004; 6: 121–128. 10.1038/ncb1087 14730312

[13] Conway KE, McConnell BB, Bowring CE, Donald CD, Warren ST, Vertino PM. TMS1, a novel proapoptotic caspase recruitment domain protein, is a target of methylation-induced gene silencing in human breast cancers. Cancer Res. 2000; 60: 6236–6242. 11103776

[14] Moriai R, Tsuji N, Kobayashi D, Yagihashi A, Namiki Y, Takahashi H, et al A proapoptotic caspase recruitment domain protein gene, TMS1, is hypermethylated in human breast and gastric cancers. Anticancer Res. 2002; 22: 4163–4168. 12553049

[15] Stone AR, Bobo W, Brat DJ, Devi NS, Van Meir EG, Vertino PM. Aberrant methylation and down-regulation of TMS1/ASC in human glioblastoma. Am J Pathol. 2004; 165: 1151–1161. 10.1016/S0002-9440(10)63376-7 15466382 PMC1618625

[16] Virmani A, Rathi A, Sugio K, Sathyanarayana UG, Toyooka S, Kischel FC, et al Aberrant methylation of TMS1 in small cell, non small cell lung cancer and breast cancer. Int J Cancer. 2003; 106: 198–204. 10.1002/ijc.11206 12800194

[17] Yokoyama T, Sagara J, Guan X, Masumoto J, Takeoka M, Komiyama Y, et al Methylation of ASC/TMS1, a proapoptotic gene responsible for activating procaspase-1, in human colorectal cancer. Cancer Lett. 2003; 202: 101–108. 14643031 10.1016/j.canlet.2003.08.027

[18] Kapoor-Vazirani P, Kagey JD, Powell DR, Vertino PM. Role of hMOF-dependent histone H4 lysine 16 acetylation in the maintenance of TMS1/ASC gene activity. Cancer Res. 2008; 68: 6819–6821. 10.1158/0008-5472.CAN-08-0141 PMC2585755 18701507

[19] Kong L, Zhang Y, Ye ZQ, Liu XQ, Zhao SQ, Wei L, et al CPC: assess the protein-coding potential of transcripts using sequence features and support vector machine. Nucleic Acids Res. 2007; 35: W345–W349. 10.1093/nar/gkm391 17631615 PMC1933232

[20] McConnell BB, Vertino PM. TMS1/ASC: the cancer connection. Apoptosis. 2004; 9: 5–18. 10.1023/B:APPT.0000012117.32430.0c 14739594

[21] Gordian E, Ramachandran K, Singal R. Methylation mediated silencing of TMS1 in breast cancer and its potential contribution to docetaxel cytotoxicity. Anticancer Res. 2009; 29: 3207–3210. 19661336

[22] Hervouet E, Vallette FM, Cartron PF. Impact of the DNA methyltransferases expression on the methylation status of apoptosis-associated genes in glioblastoma multiforme. Cell Death Dis. 2010; 1: e8 10.1038/cddis.2009.7 21364627 PMC3032516

[23] Wutz A, Rasmussen TP, Jaenisch R. Chromosomal silencing and localization are mediated by different domains of Xist RNA. Nat Genet. 2002; 30: 167–174. 10.1038/ng820 11780141

[24] Liu B, Sun L, Liu Q, Gong C, Yao Y, Lv X, et al A cytoplasmic NF-κB interacting long noncoding RNA blocks IκB phosphorylation and suppresses breast cancer metastasis. Cancer Cell. 2015; 27: 370–381. 10.1016/j.ccell.2015.02.004 25759022

[25] Chillón I, Pyle AM. Inverted repeat Alu elements in the human lincRNA-p21 adopt a conserved secondary structure that regulates RNA function. Nucleic Acids Res. 2016; 44: 9462–9471. 10.1093/nar/gkw599 27378782 PMC5100600

[26] Estève PO, Chin HG, Smallwood A, Feehery GR, Gangisetty O, Karpf AR, et al Direct interaction between DNMT1 and G9a coordinates DNA and histone methylation during replication. Genes Dev. 2006; 20: 3089–3103. 10.1101/gad.1463706 17085482 PMC1635145

[27] Pandey RR, Mondal T, Mohammad F, Enroth S, Redrup L, Komorowski J, et al Kcnq1ot1 antisense noncoding RNA mediates lineage-specific transcriptional silencing through chromatin-level regulation. Mol Cell. 2008; 32: 232–246. 10.1016/j.molcel.2008.08.022 18951091

[28] Mohammad F, Mondal T, Guseva N, Pandey GK, Kanduri C. Kcnq1ot1 noncoding RNA mediates transcriptional gene silencing by interacting with Dnmt1. Development. 2010; 137: 2493–2499. 10.1242/dev.048181 20573698

[29] Park SE, Yi HJ, Suh N, Park YY, Koh JY, Jeong SY, et al Inhibition of EHMT2/G9a epigenetically increases the transcription of Beclin-1 via an increase in ROS and activation of NF-κB. Oncotarget. 2016; 7: 39796–39808. 10.18632/oncotarget.9290 27174920 PMC5129971

[30] Li Y, Cheng C. Long noncoding RNA NEAT1 promotes the metastasis of osteosarcoma via interaction with the G9a-DNMT1-Snail complex. Am J Cancer Res. 2018; 8: 81–90. 29416922 PMC5794723

[31] Johnsson P, Ackley A, Vidarsdottir L, Lui WO, Corcoran M, Grandér D, et al A pseudogene long-noncoding-RNA network regulates PTEN transcription and translation in human cells. Nat Struct Mol Biol. 2013; 20: 440–446. 10.1038/nsmb.2516 23435381 PMC3618526

[32] Faghihi MA, Modarresi F, Khalil AM, Wood DE, Sahagan BG, Morgan TE, et al Expression of a noncoding RNA is elevated in Alzheimer′s disease and drives rapid feed-forward regulation of beta-secretase. Nat Med. 2008; 14: 723–730. 10.1038/nm1784 18587408 PMC2826895

[33] Gong C, Maquat LE. LncRNAs transactivate STAU1-mediated mRNA decay by duplexing with 3′ UTRs via Alu elements. Nature. 2011; 470: 284–288. 10.1038/nature09701 21307942 PMC3073508

[34] Yuan JH, Yang F, Wang F, Ma JZ, Guo YJ, Tao QF, et al A long noncoding RNA activated by TGF-β promotes the invasion-metastasis cascade in hepatocellular carcinoma. Cancer Cell. 2014; 25:666–681. 10.1016/j.ccr.2014.03.010 24768205

[35] Yuan JH, Liu XN, Wang TT, Pan W, Tao QF, Zhou WP, et al The MBNL3 splicing factor promotes hepatocellular carcinoma by increasing PXN expression through the alternative splicing of lncRNA-PXN-AS1. Nat Cell Biol. 2017; 19: 820–832. 10.1038/ncb3538 28553938

[36] Zhang Y, Pitchiaya S, Cieślik M, Niknafs YS, Tien JC, Hosono Y, et al Analysis of the androgen receptor-regulated lncRNA landscape identifies a role for ARLNC1 in prostate cancer progression. Nat Genet. 2018; 50: 814–824. 10.1038/s41588-018-0120-1 29808028 PMC5980762

[37] Jadaliha M, Gholamalamdari O, Tang W, Zhang Y, Petracovici A, Hao Q, et al A natural antisense lncRNA controls breast cancer progression by promoting tumor suppressor gene mRNA stability. PLoS Genet. 2018; 14: e1007802 10.1371/journal.pgen.1007802 30496290 PMC6289468

[38] Lee JT. Epigenetic regulation by long noncoding RNAs. Science. 2012; 338: 1435–1439. 10.1126/science.1231776 23239728

[39] Hashimoto H, Vertino PM, Cheng X. Molecular coupling of DNA methylation and histone methylation. Epigenomics. 2010; 2: 657–669. 10.2217/epi.10.44 21339843 PMC3039846

[40] Chang Y, Sun L, Kokura K, Horton JR, Fukuda M, Espejo A, et al MPP8 mediates the interactions between DNA methyltransferase Dnmt3a and H3K9 methyltransferase GLP/G9a. Nat Commun. 2011; 2: 533 10.1038/ncomms1549 22086334 PMC3286832

[41] Li D, Zhang J, Wang M, Li X, Gong H, Tang H, et al Activity dependent LoNA regulates translation by coordinating rRNA transcription and methylation. Nat Commun. 2018; 9: 1726 10.1038/s41467-018-04072-4 29712923 PMC5928123

[42] Jandura A, Krause HM. The new RNA world: growing evidence for long noncoding RNA functionality. Trends Genet. 2017; 33: 665–676. 10.1016/j.tig.2017.08.002 28870653

[43] Merry CR, Forrest ME, Sabers JN, Beard L, Gao XH, Hatzoglou M, et al DNMT1-associated long non-coding RNAs regulate global gene expression and DNA methylation in colon cancer. Hum Mol Genet. 2015; 24: 6240–6253. 10.1093/hmg/ddv343 26307088 PMC4599679

[44] Hendrickson DG, Kelley DR, Tenen D, Bernstein B, Rinn JL. Widespread RNA binding by chromatin-associated proteins. Genome Biol. 2016; 17: 28 10.1186/s13059-016-0878-3 26883116 PMC4756407

[45] Long Y, Wang X, Youmans DT, Cech TR. How do lncRNAs regulate transcription? Sci Adv. 2017; 3: eaao2110 10.1126/sciadv.aao2110 28959731 PMC5617379

[46] Di Ruscio A, Ebralidze AK, Benoukraf T, Amabile G, Goff LA, Terragni J, et al DNMT1-interacting RNAs block gene-specific DNA methylation. Nature. 2013; 503: 371–376. 10.1038/nature12598 24107992 PMC3870304

[47] Chalei V, Sansom SN, Kong L, Lee S, Montiel JF, Vance KW, et al The long non-coding RNA Dali is an epigenetic regulator of neural differentiation. Elife. 2014; 3: e04530 10.7554/eLife.04530 25415054 PMC4383022

[48] Yoon JH, Abdelmohsen K, Srikantan S, Yang X, Martindale JL, De S, et al LincRNA-p21 suppresses target mRNA translation. Mol Cell. 2012; 47: 648–655. 10.1016/j.molcel.2012.06.027 22841487 PMC3509343

[49] Carrieri C, Cimatti L, Biagioli M, Beugnet A, Zucchelli S, Fedele S, et al Long non-coding antisense RNA controls Uchl1 translation through an embedded SINEB2 repeat. Nature. 2012; 491: 454–457. 10.1038/nature11508 23064229

[50] Zhou W, Liu Y, Li S, Guo D, Sun Q, Jin J, et al Long noncoding RNA GMAN, up-regulated in gastric cancer tissue, is associated with metastasis in patients and promotes translation of Ephrin A1 by competitively binding GMAN-AS. Gastroenterology. 2019; 156: 676–691.e11. 10.1053/j.gastro.2018.10.054 30445010

[51] Katayama S, Tomaru Y, Kasukawa T, Waki K, Nakanishi M, Nakamura M, et al Antisense transcription in the mammalian transcriptome. Science. 2005; 309: 1564–1566. 10.1126/science.1112009 16141073

[52] Sigova AA, Mullen AC, Molinie B, Gupta S, Orlando DA, Guenther MG, et al Divergent transcription of long noncoding RNA/mRNA gene pairs in embryonic stem cells. Proc Natl Acad Sci USA. 2013; 110: 2876–2881. 10.1073/pnas.1221904110 23382218 PMC3581948

[53] Werner MS, Ruthenburg AJ. Nuclear fractionation reveals thousands of chromatin-tethered noncoding RNAs adjacent to active genes. Cell Rep. 2015; 12:1089–1098. 10.1016/j.celrep.2015.07.033 26257179 PMC5697714

