# Supplementary figures and images for: A long noncoding RNA distributed in both nucleus and cytoplasm operates in the PYCARD-regulated apoptosis by coordinating the epigenetic and translational regulation
Source: PLoS Genet. 2019 May 14;15(5):e1008144. doi: 10.1371/journal.pgen.1008144 (PMC6534332; doi:10.1371/journal.pgen.1008144)

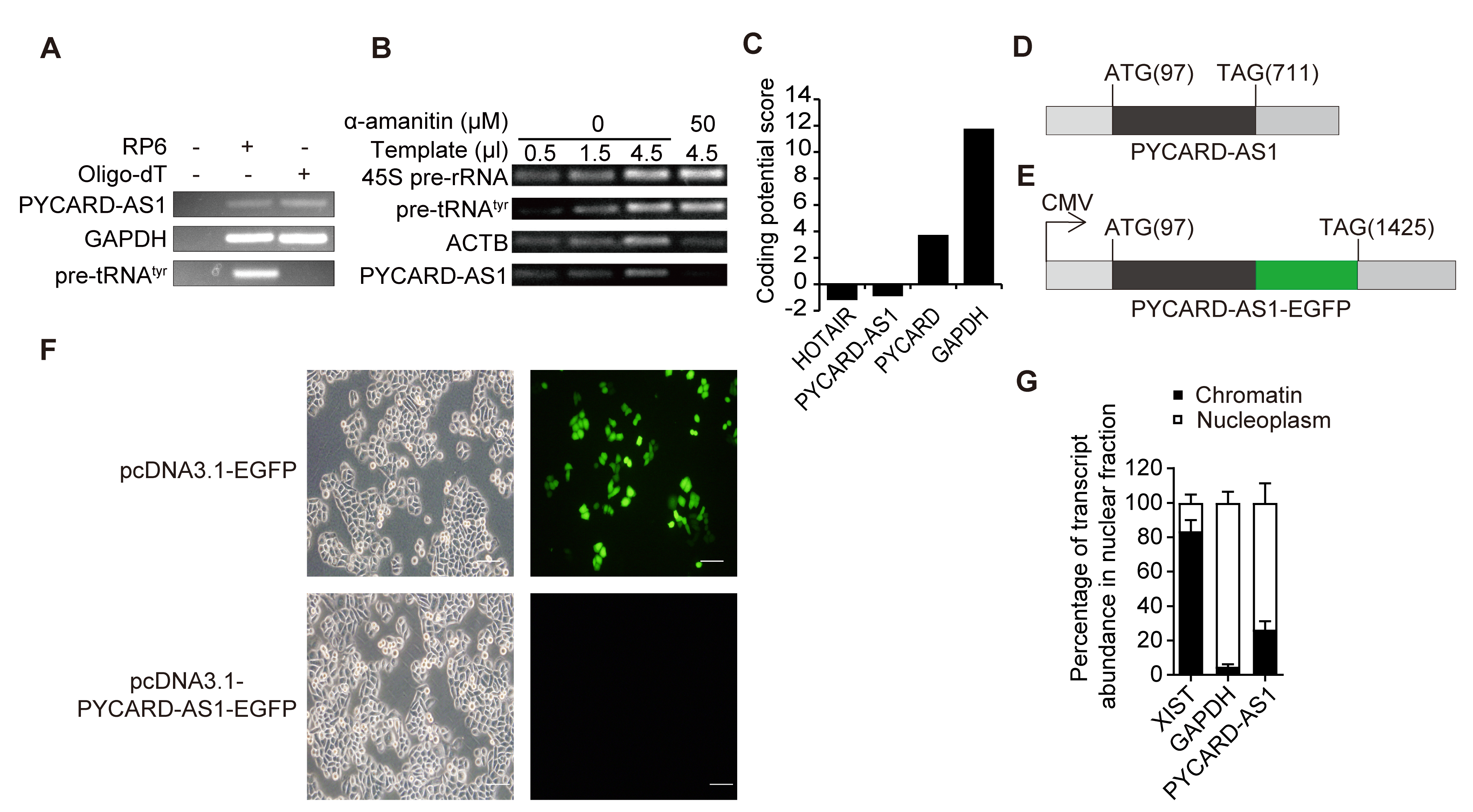

Supplement: S1 Fig — (A) PYCARD-AS1 is poly(A)-tailed. Total RNA extracted from SKBR3 cells was reverse-transcribed using Oligo-dT or random hexamers (RP6). GAPDH mRNA served as a control polyadenylated transcript, and pre-tRNAtyr served as a control non-polyadenylated transcript. (B) PYCARD-AS1 transcription is sensitive to α-amanitin. SKBR3 cells were treated with α-amanitin at a concentration of either 0 or 50 μM for 8 h. Total RNA was prepared from these cells, and the levels of the indicated transcripts were determined by RT-PCR. Increasing amounts of cDNA template from the untreated cells (lanes 1–3) were used for PCR to test whether the PCR amplification occurs quantitatively. 45S pre-rRNA, pre-tRNAtyr and ACTB were used as controls. (C) PYCARD-AS1 was predicted to be a noncoding transcript. The RNA sequences of PYCARD-AS1, PYCARD, GAPDH and HOTAIR were put into the CPC program, with HOTAIR serving as a control noncoding transcript, while PYCARD and GAPDH served as control coding transcripts. Scores above 0 suggest coding potential, whereas scores below 0 represent no coding potential. (D, E) The ORF analysis of PYCARD-AS1 sequence by UniProt (D) and the diagram of fusion gene PYCARD-AS1-EGFP inserted in pcDNA3.1 plasmid. (F) Phase contrast or fluorescence microscopy of SKBR3 cells that had been transfected with the indicated plasmid (scale bars, 100 μm). (G) qRT-PCR assays detecting the distribution of the indicated transcripts in chromatin and nucleoplasm extract from SKBR3 cells. XIST, a canonically chromatin-associated lncRNA, and the protein-coding GAPDH mRNA, were assessed as controls to confirm the findings of our chromatin fractionation. The qRT-PCR data, represented as a percentage of the detected transcripts in nuclear fraction, are presented as means ± SD from three independent experiments performed in triplicate. (TIF) [file pgen.1008144.s001.tif]

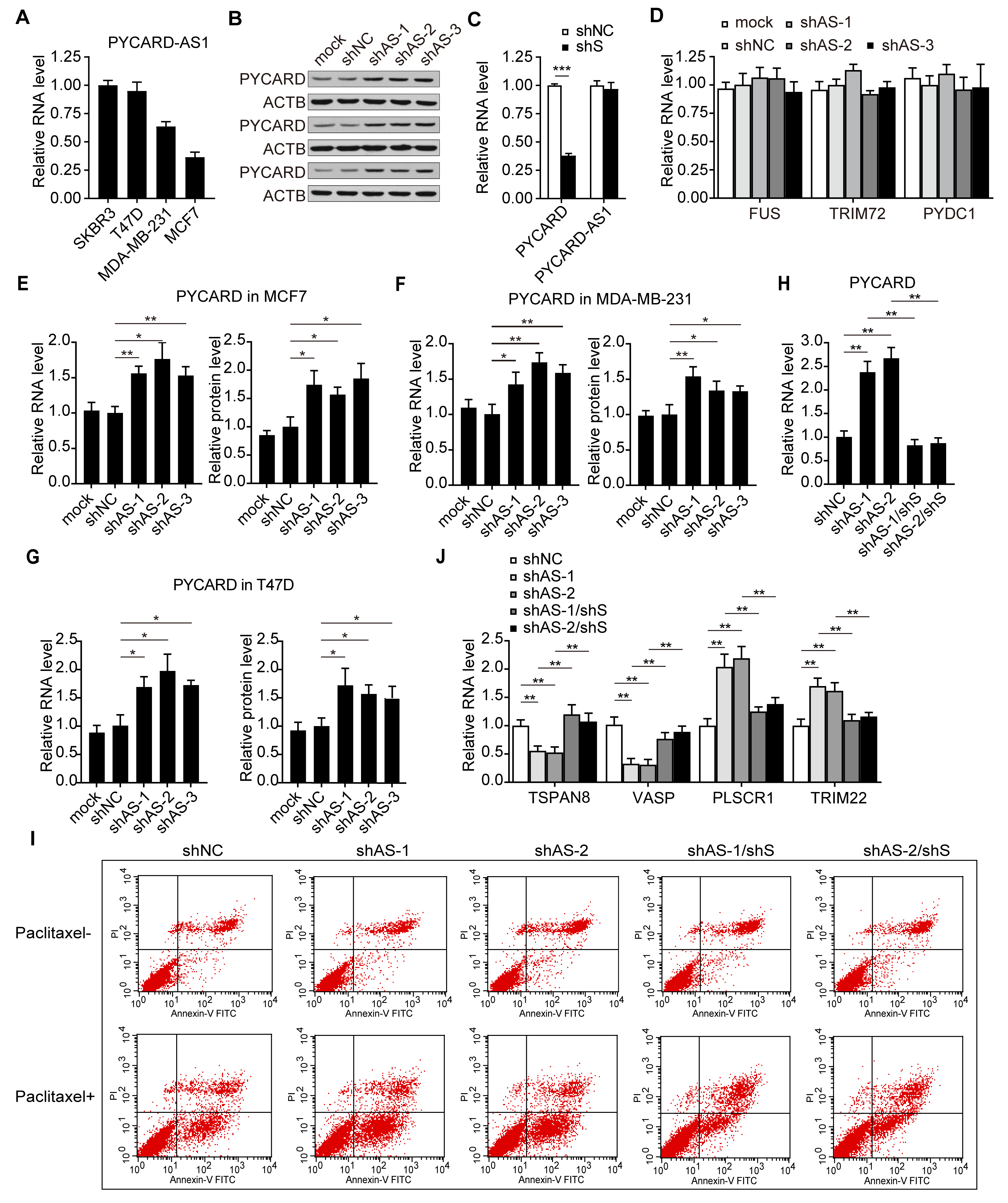

Supplement: S2 Fig — (A) qRT-PCR measuring expression level of PYCARD-AS1 in the indicated breast cancer lines relative to the level in SKBR3 cells. 18S rRNA was used as an internal control to normalize the amount of total RNA in the samples. (B) The replicate blots subjected to the densitometric analysis in Fig 2H. (C) qRT-PCR detecting the effect of PYCARD knockdown on PYCARD-AS1 level in SKBR3 cells. (D) qRT-PCR detecting the effect of PYCARD-AS1 knockdown on the mRNA levels of FUS, TRIM72 and PYDC1 in SKBR3 cells. (E–G) qRT-PCR (left) and immunoblotting (right) detecting the effect of PYCARD-AS1 knockdown on PYCARD expression in MCF7 (E), MDA-MB-231 (F) and T47D (G) cells. (H) qRT-PCR detecting the abundance of PYCARD in SKBR3 cells after PYCARD-AS1 knockdown and simultaneous PYCARD knockdown. (I) Representative plots of apoptosis of the indicated SKBR3 cells with or without paclitaxel treatment. (J) qRT-PCR of a representative panel of PYCARD-AS1- and PYCARD-regulated genes in the indicated SKBR3 cells. In this figure, the qRT-PCR data are presented as means ± SD from three independent experiments performed in triplicate; for immunoblotting, signals from three independent assays were subjected to densitometric analysis, and the data are presented as means ± SD; * p < 0.05; ** p < 0.01; *** p < 0.001. (TIF) [file pgen.1008144.s002.tif]

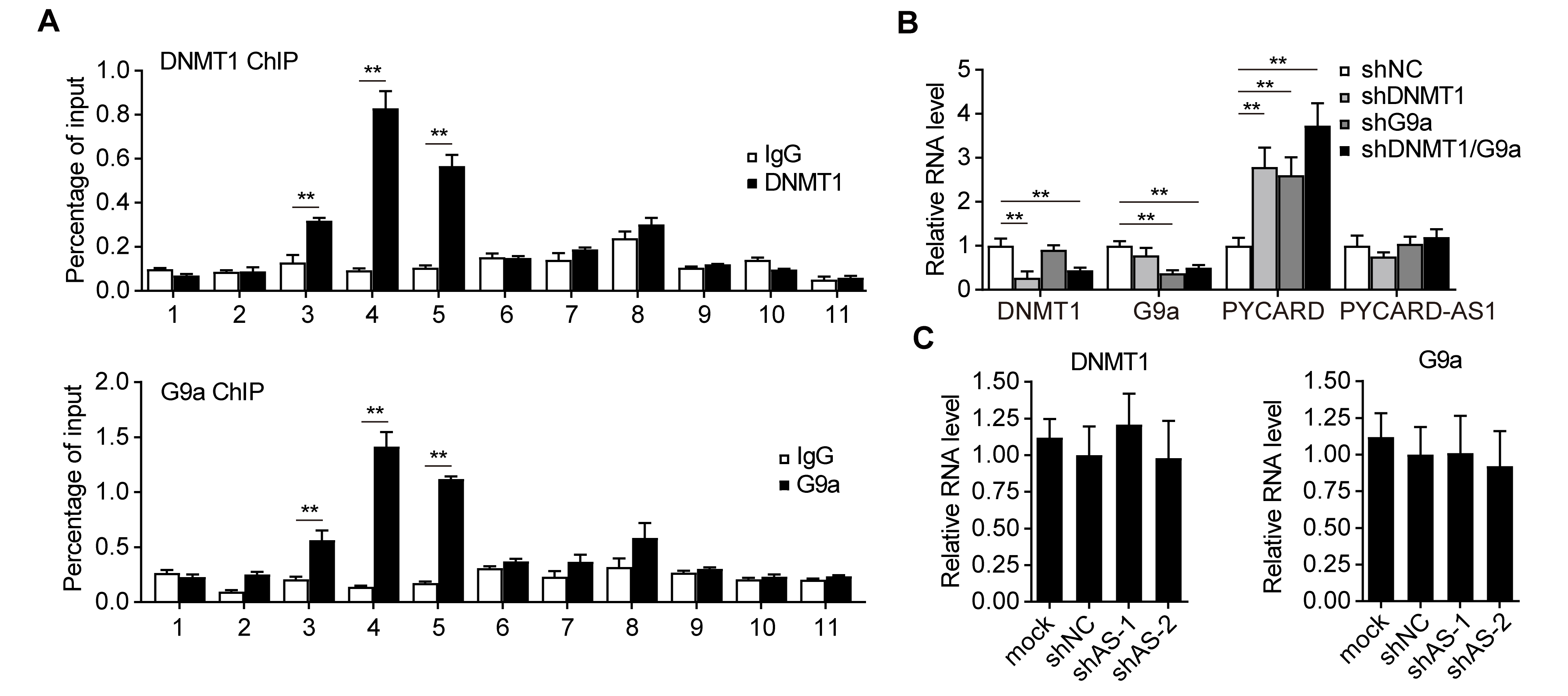

Supplement: S3 Fig — (A) ChIP assays detecting association of DNMT1 (upper) and G9a (lower) with the segments shown in Fig 3A in SKBR3 cells. (B) qRT-PCR detecting the levels of DNMT1, G9a, PYCARD and PYCARD-AS1 in the indicated SKBR3 cells. (C) qRT-PCR detecting the levels of DNMT1 and G9a in SKBR3 cells with or without PYCARD-AS1 knockdown. Data in this figure are presented as mean ± SD from three independent experiments performed in triplicate; **p < 0.01. (TIF) [file pgen.1008144.s003.tif]

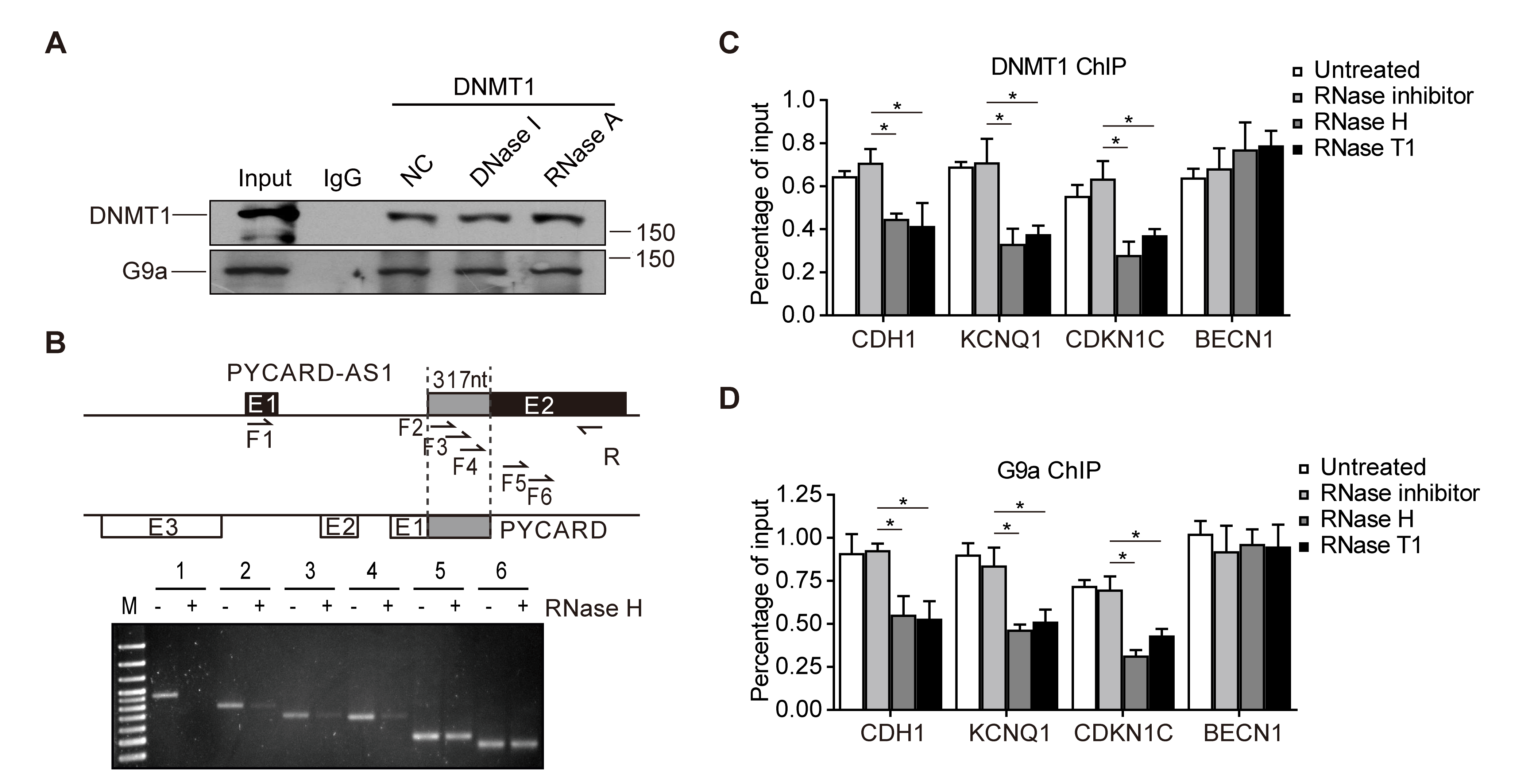

Supplement: S4 Fig — (A) The interaction between DNMT1 and G9a confirmed by DNMT1 IP followed by immunoblotting. The interaction was not abolished by DNase I or RNase A treatment. (B) Semi-quantitative RT-PCR detecting the PYCARD-AS1 region associated with the PYCARD locus in SKBR3 cells after the permeabilization treatment and the treatment with an RNase H or RNase inhibitor. The reverse transcription reaction was initiated by a PYCARD-AS1-specific reverse primer (R, shown schematically), which was paired with each forward walking primers (F1–F6, shown schematically) in the subsequent PCR amplification. (C, D) RNase-ChIP assays detecting the association of DNMT1 (C) or G9a (D) with the indicated gene promoters. SKBR3 cells were permeabilized and treated with an RNase inhibitor, RNase H or RNase T1, in advance. Untreated SKBR3 cells were also included. In (C and D), data are presented as mean ± SD from three independent experiments performed in triplicate; * p < 0.05. (TIF) [file pgen.1008144.s004.tif]

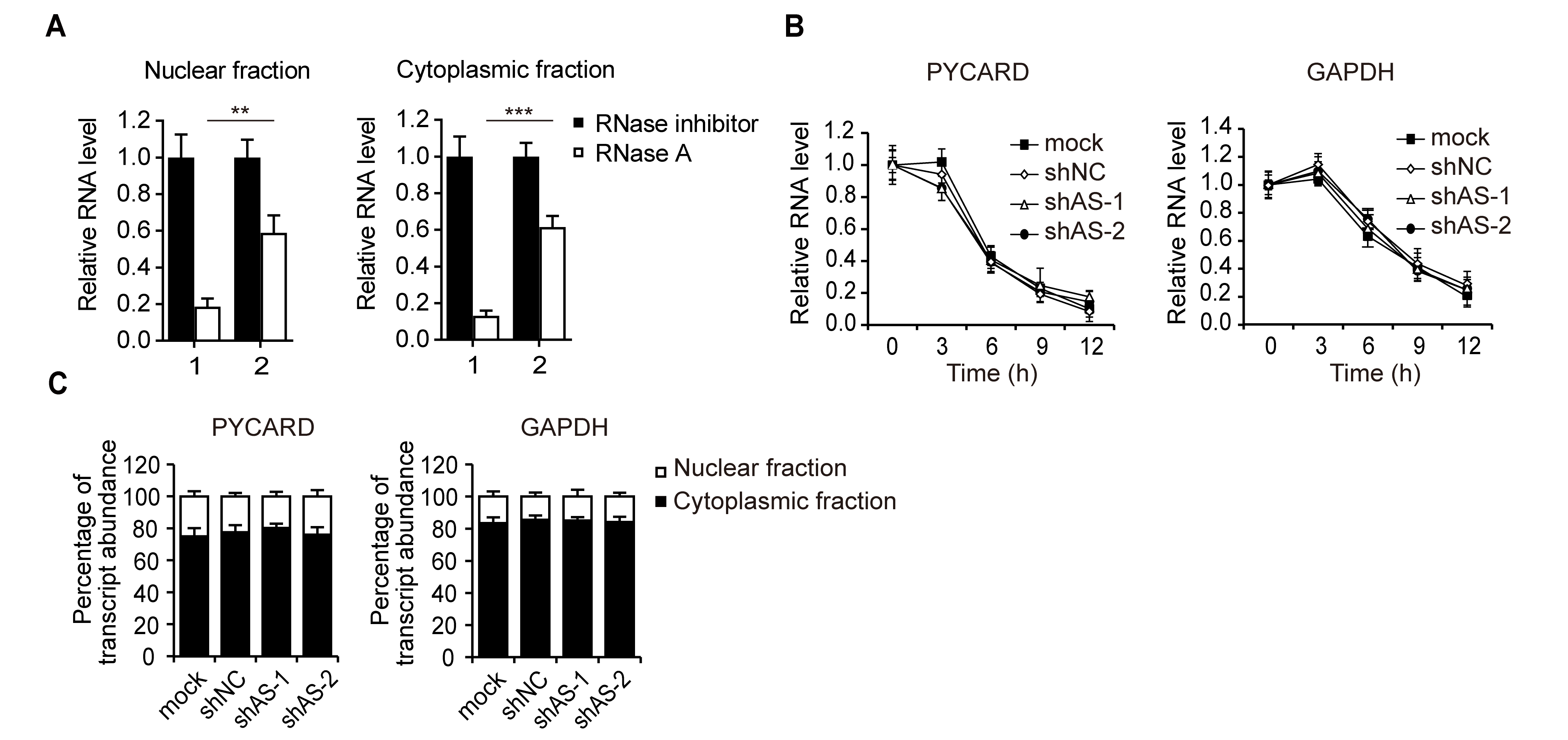

Supplement: S5 Fig — (A) RNase-A assay detecting the interaction between PYCARD-AS1 and PYCARD transcripts in the nucleus (left) and cytoplasm (right). Nuclear and cytoplasmic lysates were prepared from SKBR3 cells, and the lysates were subjected to RNase-A treatment, RNA extraction and qRT-PCR analysis to detect the non-overlapping and overlapping regions (1 and 2) described in Fig 6B. (B) The stability of PYCARD (left) and GAPDH (right) mRNAs over time was measured by qRT-PCR relative to the start time point after blocking new RNA synthesis with α-amanitin in SKBR3 cells with or without PYCARD-AS1 knockdown and normalized to 18S rRNA. (C) qRT-PCR analysis following nuclear/cytoplasmic fractionation detecting the distribution of PYCARD (left) and GAPDH (right) mRNAs in SKBR3 cells with or without PYCARD-AS1 knockdown. Data in this figure are presented as means ± SD from three independent experiments performed in triplicate; ** p < 0.01; *** p < 0.001. (TIF) [file pgen.1008144.s005.tif]
