# Supplementary material for: A long noncoding RNA distributed in both nucleus and cytoplasm operates in the PYCARD-regulated apoptosis by coordinating the epigenetic and translational regulation
Source: PLoS Genet. 2019 May 14;15(5):e1008144. doi: 10.1371/journal.pgen.1008144 (PMC6534332; doi:10.1371/journal.pgen.1008144)
Supplement: S1 Table — (DOCX) [file pgen.1008144.s006.docx]

| **S1 Table. List of genes that are regulated by PYCARD-AS1 knockdown and PYCARD-AS1/PYCARD double-knockdown** | | | | | | | | | | |
| --- | --- | --- | --- | --- | --- | --- | --- | --- | --- | --- |
| **S1_1 Table. List of genes that are regulated by PYCARD-AS1 knockdown (shAS-1 versus shNC)** | | | | | | | | | | |
| **ProbeSetID** | **Target transcript ID** | | **Gene symbol** | | | | **Fold change** | | **p value** | |
| 11716384_at | NM_002982.3 | | CCL2 | | | | 2.0061 | | 0.01074 | |
| 11740613_a_at | BC009006.1 | | TMEM30A | | | | 2.008 | | 0.02166 | |
| 11752384_s_at | AK299396.1 | | COL6A1 | | | | 2.0129 | | 0.04286 | |
| 11729020_at | NM_194250.1 | | ZNF804A | | | | 2.0142 | | 0.01908 | |
| 11749510_a_at | AK304187.1 | | SAMHD1 | | | | 2.0194 | | 0.03183 | |
| 11744829_s_at | AK296822.1 | | HLA-E | | | | 2.021 | | 0.01195 | |
| 11748489_s_at | AK301821.1 | | MAGED2 | | | | 2.0227 | | 0.00652 | |
| 11757396_s_at | BU675268 | | ANXA8 | | | | 2.0321 | | 0.01856 | |
| 11751698_a_at | AK301560.1 | | EAF2 | | | | 2.0354 | | 0.03004 | |
| 11728022_a_at | NM_018004.1 | | TMEM45A | | | | 2.037 | | 0.02207 | |
| 11728792_x_at | NM_013302.3 | | EEF2K | | | | 2.0396 | | 0.00245 | |
| 11725861_a_at | NM_001080416.2 | | MYBL1 | | | | 2.0446 | | 0.0161 | |
| 11757942_s_at | BM998048 | | UTRN | | | | 2.0484 | | 0.04597 | |
| 11723181_a_at | NM_002747.3 | | MAPK4 | | | | 2.0491 | | 0.00829 | |
| 11720028_x_at | BC014514.1 | | LDLR | | | | 2.0514 | | 0.01732 | |
| 11724917_at | AK023165.1 | | TMTC3 | | | | 2.5576 | | 0.02774 | |
| 11716876_a_at | NM_003313.3 | | TSTA3 | | | | 2.401 | | 0.01635 | |
| 11730614_a_at | NM_003038.3 | | SLC1A4 | | | | 2.0634 | | 0.0111 | |
| 11716413_x_at | NM_175617.3 | | MT1E | | | | 2.0738 | | 0.00559 | |
| 11728451_a_at | NM_013363.2 | | PCOLCE2 | | | | 2.0756 | | 0.01443 | |
| 11719628_a_at | NM_001135565.1 | | HDHD1 | | | | 2.0785 | | 0.00801 | |
| 11745300_a_at | BX647203.1 | | DNAJC6 | | | | 2.0787 | | 0.01828 | |
| 11717743_a_at | NM_007274.3 | | ACOT7 | | | | 2.0811 | | 0.01838 | |
| 11727440_at | BC007522.1 | | LOXL4 | | | | 2.0849 | | 0.00772 | |
| 11715536_a_at | NM_002168.2 | | IDH2 | | | | 2.0856 | | 0.01142 | |
| 11756141_s_at | DW454053 | | KDELR2 | | | | 2.0891 | | 0.00402 | |
| 11722355_s_at | NM_001343.2 | | DAB2 | | | | 2.1049 | | 0.05419 | |
| 11746440_s_at | AK293345.1 | | GLO1 | | | | 2.015 | | 0.02168 | |
| 11721864_s_at | NM_207113.1 | | SLC37A3 | | | | 2.1066 | | 0.00768 | |
| 11728300_at | NM_057749.1 | | CCNE2 | | | | 2.11 | | 0.02398 | |
| 11720087_a_at | NM_005013.2 | | NUCB2 | | | | 2.1107 | | 0.0469 | |
| 11735140_at | NM_003811.3 | | TNFSF9 | | | | 2.1113 | | 0.01755 | |
| 11726774_a_at | NM_005060.3 | | RORC | | | | 2.1039 | | 0.01568 | |
| 11736061_a_at | NM_002639.4 | | SERPINB5 | | | | 2.1169 | | 0.02493 | |
| 11743626_s_at | CA450422 | | LGR4 | | | | 2.1227 | | 0.01414 | |
| 11725804_a_at | NM_001100420.1 | | C21orf91 | | | | 2.1256 | | 0.01848 | |
| 11719075_s_at | NM_022912.2 | | REEP1 | | | | 2.1394 | | 0.01401 | |
| 11733085_a_at | NM_001976.3 | | ENO3 | | | | 2.1462 | | 0.02248 | |
| 11722093_a_at | NM_000943.4 | | PPIC | | | | 2.0433 | | 0.0532 | |
| 11757504_a_at | BQ188977 | | DDAH1 | | | | 2.1681 | | 0.0179 | |
| 11720780_a_at | NM_020775.2 | | KIAA1324 | | | | 2.1691 | | 0.02493 | |
| 11722152_s_at | NM_004836.4 | | EIF2AK3 | | | | 2.1759 | | 0.01398 | |
| 11725951_at | AF013591.1 | | RIOK3 | | | | 2.1768 | | 0.04012 | |
| 11755284_s_at | AK093487.1 | | TSPAN13 | | | | 2.1848 | | 0.01737 | |
| 11716167_a_at | NM_001144925.1 | | MX1 | | | | 2.199 | | 0.0043 | |
| 11743352_s_at | AB023194.1 | | COBLL1 | | | | 2.1992 | | 0.03427 | |
| 11715557_at | CR597598.1 | | FLJ23867 | | | | 2.2011 | | 0.02941 | |
| 11725198_at | NM_000575.3 | | IL1A | | | | 2.2046 | | 0.01481 | |
| 11755620_a_at | BC025774.1 | | HYAL1 | | | | 2.2096 | | 0.01148 | |
| 11726479_a_at | NM_002463.1 | | MX2 | | | | 2.2126 | | 0.00809 | |
| 11724117_x_at | AI038997 | | SAMD9L | | | | 2.2197 | | 0.01627 | |
| 11739302_a_at | AB012911.1 | | FZD6 | | | | 2.2199 | | 0.03301 | |
| 11726113_a_at | NM_052943.3 | | FAM46B | | | | 2.2212 | | 0.03578 | |
| 11754948_a_at | AK096462.1 | | HECTD3 | | | | 2.2225 | | 0.06363 | |
| 11730719_x_at | AF087876.1 | | AP1S2 | | | | 2.2274 | | 0.0492 | |
| 11724488_at | NM_020299.4 | | AKR1B10 | | | | 2.0304 | | 0.03082 | |
| 11744178_a_at | CR592313.1 | | C19orf66 | | | | 2.2432 | | 0.04651 | |
| 11729238_a_at | NM_001321.1 | | CSRP2 | | | | 2.2461 | | 0.01305 | |
| 11722879_a_at | NM_181674.2 | | PPP2R2B | | | | 2.251 | | 0.01701 | |
| 11720669_at | NM_003884.4 | | KAT2B | | | | 2.2518 | | 0.00667 | |
| 11732636_at | NM_024746.3 | | HHIPL2 | | | | 2.2644 | | 0.08528 | |
| 11723669_s_at | NM_006379.2 | | SEMA3C | | | | 2.2776 | | 0.00565 | |
| 11747808_a_at | AK301830.1 | | WIPI1 | | | | 2.2822 | | 0.03136 | |
| 11737299_at | NM_152304.1 | | RAB42 | | | | 2.2824 | | 0.01519 | |
| 11723151_s_at | NM_024728.1 | | SUGCT | | | | 2.288 | | 0.01602 | |
| 11719314_a_at | NM_017881.2 | | NMRK1 | | | | 2.2896 | | 0.00694 | |
| 11760254_at | DB350079 | | IFI44 | | | | 7.5974 | | 0.01741 | |
| 11757702_s_at | BF063817 | | DSC2 | | | | 2.2972 | | 0.07057 | |
| 11739232_x_at | AA827918 | | RASSF2 | | | | 2.3128 | | 0.02961 | |
| 11721010_a_at | NM_030792.6 | | GDPD5 | | | | 2.3205 | | 0.03726 | |
| 11718179_a_at | AI928241 | | FERMT2 | | | | 2.2389 | | 0.0112 | |
| 11723007_a_at | AB033080.2 | | CCPG1 | | | | 2.3354 | | 0.02865 | |
| 11750531_a_at | NM_013258.3 | | PYCARD | | | | 2.3374 | | 0.04221 | |
| 11758573_s_at | AI689215 | | MORC4 | | | | 2.3386 | | 0.00777 | |
| 11723051_at | NM_005130.3 | | FGFBP1 | | | | 2.3389 | | 0.01428 | |
| 11723234_at | AB116553.1 | | IFI44L | | | | 2.3484 | | 0.01829 | |
| 11718332_a_at | AK293677.1 | | TSPAN3 | | | | 2.3623 | | 0.02331 | |
| 11722909_a_at | NM_178831.5 | | GATS | | | | 2.3841 | | 0.04874 | |
| 11741555_s_at | NM_001127605.1 | | LIPA | | | | 2.3934 | | 0.0474 | |
| 11721897_a_at | NM_024119.2 | | DHX58 | | | | 2.3951 | | 0.00086 | |
| 11741704_x_at | NM_003526.2 | | HIST1H2BC | | | | 3.4198 | | 0.00652 | |
| 11738185_s_at | NM_001029851.1 | | PDE8B | | | | 2.4023 | | 0.05877 | |
| 11717644_a_at | NM_001136557.1 | | GPR107 | | | | 2.4081 | | 0.00983 | |
| 11716895_s_at | NM_005101.3 | | ISG15 | | | | 2.4481 | | 0.06151 | |
| 11749922_x_at | BC065829.1 | | TAGLN | | | | 1.7712 | | 0.02758 | |
| 11726770_x_at | BC073156.1 | | XAF1 | | | | 2.4621 | | 0.0466 | |
| 11731831_x_at | NM_138413.3 | | HOGA1 | | | | 2.4795 | | 0.12174 | |
| 11722503_at | NM_002214.2 | | ITGB8 | | | | 2.481 | | 0.00602 | |
| 11761536_a_at | AK297048.1 | | TM2D3 | | | | 2.4945 | | 0.06933 | |
| 11719833_at | NM_005797.2 | | MPZL2 | | | | 2.5106 | | 0.02879 | |
| 11727553_a_at | BC093027.1 | | PKIB | | | | 2.5214 | | 0.02249 | |
| 11763173_s_at | CA417761 | | PGM3 | | | | 2.5298 | | 0.04603 | |
| 11724399_a_at | NM_001012967.1 | | DDX60L | | | | 2.5469 | | 0.03985 | |
| 11715576_at | AF136373.1 | | RAC1 | | | | 2.5705 | | 0.00025 | |
| 11740602_s_at | NM_001145646.1 | | APH1B | | | | 2.5708 | | 0.00337 | |
| 11731137_a_at | AF328296.1 | | MYPN | | | | 2.5747 | | 0.0287 | |
| 11715670_a_at | NM_003641.3 | | IFITM1 | | | | 2.5932 | | 0.01648 | |
| 11755762_a_at | BC144510.1 | | TMEM2 | | | | 2.6122 | | 0.03024 | |
| 11719764_s_at | NM_212503.1 | | CDK18 | | | | 3.0159 | | 0.0483 | |
| 11725498_a_at | NM_032256.1 | | TMEM117 | | | | 2.6911 | | 0.0257 | |
| 11718231_x_at | NM_018950.2 | | HLA-F | | | | 2.6386 | | 0.00037 | |
| 11748907_a_at | AK293357.1 | | RARRES3 | | | | 2.7249 | | 0.00025 | |
| 11721874_at | BC032839.2 | | IFIT2 | | | | 2.7302 | | 0.00779 | |
| 11729243_s_at | U03272.1 | | FBN2 | | | | 2.7327 | | 0.02718 | |
| 11726364_x_at | NM_003733.2 | | OASL | | | | 2.7364 | | 0.00396 | |
| 11718916_a_at | NM_004031.2 | | IRF7 | | | | 2.7389 | | 0.03655 | |
| 11719491_a_at | NM_005533.3 | | IFI35 | | | | 2.7494 | | 0.03315 | |
| 11717575_at | NM_005398.4 | | PPP1R3C | | | | 2.7573 | | 0.0274 | |
| 11755147_s_at | BX640607.1 | | STAT2 | | | | 2.7603 | | 6.5E-06 | |
| 11737847_a_at | NM_001531.1 | | MR1 | | | | 2.7711 | | 0.0323 | |
| 11720704_s_at | NM_001003395.1 | | TPD52L1 | | | | 2.7723 | | 0.00863 | |
| 11718435_a_at | NM_001033047.1 | | NPNT | | | | 2.7777 | | 0.02567 | |
| 11745624_s_at | BC142661.1 | | INADL | | | | 2.7786 | | 0.01278 | |
| 11723128_a_at | NM_017912.3 | | HERC6 | | | | 2.7929 | | 0.0216 | |
| 11741315_a_at | NM_001128305.1 | | PLSCR4 | | | | 2.8842 | | 0.06259 | |
| 11755819_a_at | AK125989.1 | | DDX58 | | | | 2.8896 | | 0.03682 | |
| 11742232_s_at | NM_001040020.1 | | FAM3C | | | | 2.8998 | | 0.0228 | |
| 11735987_at | NM_032935.2 | | MT4 | | | | 2.9114 | | 0.03107 | |
| 11721994_s_at | NM_198183.1 | | UBE2L6 | | | | 2.9541 | | 0.00978 | |
| 11757430_s_at | BX373810 | | TMED5 | | | | 2.9554 | | 0.00706 | |
| 11721335_x_at | NM_182943.2 | | PLOD2 | | | | 2.9557 | | 0.00711 | |
| 11728954_a_at | NM_000899.3 | | KITLG | | | | 2.9662 | | 0.0222 | |
| 11744236_a_at | AW150758 | | DDX60 | | | | 2.9734 | | 0.00076 | |
| 11724346_a_at | NM_022168.2 | | IFIH1 | | | | 2.9947 | | 0.01188 | |
| 11725999_s_at | AK290978.1 | | FAS | | | | 3.0031 | | 0.04328 | |
| 11743353_s_at | BM975358 | | STAT1 | | | | 3.017 | | 0.00819 | |
| 11746798_x_at | AK298939.1 | | SDC2 | | | | 3.0339 | | 0.00991 | |
| 11757650_s_at | BM997052 | | FN1 | | | | 3.0419 | | 0.00533 | |
| 11719268_at | NM_003280.2 | | TNNC1 | | | | 3.071 | | 0.04478 | |
| 11717275_s_at | BC008760.2 | | COL5A1 | | | | 3.0053 | | 0.02272 | |
| 11723329_a_at | NM_001127662.1 | | GSN | | | | 3.1797 | | 0.00315 | |
| 11728764_a_at | NM_030916.2 | | PVRL4 | | | | 3.2265 | | 0.02431 | |
| 11724463_a_at | NM_001005340.1 | | GPNMB | | | | 3.2536 | | 0.00671 | |
| 11749773_x_at | AK303893.1 | | GBP1 | | | | 3.267 | | 0.04855 | |
| 11723854_at | NM_017654.2 | | SAMD9 | | | | 3.268 | | 0.00458 | |
| 11724885_at | NM_004669.2 | | CLIC3 | | | | 3.2879 | | 0.03541 | |
| 11755400_s_at | BX647113.1 | | STK39 | | | | 3.2985 | | 0.0055 | |
| 11724378_s_at | BC090931.1 | | PAG1 | | | | 3.3661 | | 0.00558 | |
| 11721349_s_at | NM_017414.3 | | LOC102725067 | | | | 3.3662 | | 0.04929 | |
| 11731682_at | NM_001252.3 | | CD70 | | | | 3.42 | | 0.00085 | |
| 11715388_s_at | NM_078467.1 | | CDKN1A | | | | 3.4892 | | 0.01115 | |
| 11731657_s_at | NM_001079874.1 | | VAV3 | | | | 3.5063 | | 0.00875 | |
| 11731819_a_at | NM_017752.2 | | TBC1D8B | | | | 3.5215 | | 0.00276 | |
| 11733213_x_at | NM_144975.3 | | SLFN5 | | | | 3.5585 | | 0.01276 | |
| 11741581_s_at | NM_130798.1 | | SNAP23 | | | | 3.6496 | | 0.00532 | |
| 11722244_at | NM_020665.3 | | TMEM27 | | | | 3.682 | | 0.03349 | |
| 11744434_a_at | AF307339.1 | | PARP9 | | | | 3.6873 | | 0.03481 | |
| 11715988_x_at | NM_001002027.1 | | ATP5G1 | | | | 3.7105 | | 0.01295 | |
| 11751651_s_at | AK300181.1 | | PLSCR1 | | | | 3.7277 | | 0.02774 | |
| 11724538_a_at | BC021281.2 | | ABCG2 | | | | 3.7764 | | 0.00932 | |
| 11755446_s_at | AK222631.1 | | ANKH | | | | 3.8003 | | 0.00077 | |
| 11716308_s_at | NM_024843.3 | | CYBRD1 | | | | 3.8408 | | 0.01523 | |
| 11755587_a_at | X82200.1 | | TRIM22 | | | | 3.9437 | | 0.02435 | |
| 11725188_a_at | BX640971.1 | | GPR126 | | | | 3.9712 | | 0.0059 | |
| 11715620_a_at | NM_001885.1 | | CRYAB | | | | 3.9921 | | 0.00307 | |
| 11724325_a_at | NM_014857.3 | | RABGAP1L | | | | 4.0148 | | 0.00489 | |
| 11720209_at | NM_006084.4 | | IRF9 | | | | 4.1036 | | 0.04872 | |
| 11753554_a_at | BC128163.1 | | SPINK13 | | | | 4.2625 | | 0.0089 | |
| 11722963_a_at | NM_016429.2 | | COPZ2 | | | | 4.3691 | | 0.0117 | |
| 11724256_s_at | NM_016816.2 | | OAS1 | | | | 4.4922 | | 0.00269 | |
| 11764077_s_at | AW150861 | | EDIL3 | | | | 4.6284 | | 0.03488 | |
| 11730370_a_at | BM992839 | | EIF4E3 | | | | 4.9338 | | 0.00623 | |
| 11719447_s_at | BC073163.1 | | GBP2 | | | | 4.9725 | | 0.02729 | |
| 11721902_a_at | NM_005025.4 | | SERPINI1 | | | | 5.117 | | 0.0081 | |
| 11756820_a_at | AK308688.1 | | IFIT1 | | | | 5.9732 | | 0.00148 | |
| 11726201_a_at | NM_016817.2 | | OAS2 | | | | 6.0631 | | 0.02673 | |
| 11731606_at | NM_001112706.2 | | SCIN | | | | 6.6591 | | 0.00304 | |
| 11733025_at | AB026833.1 | | CLCA2 | | | | 6.9222 | | 0.0011 | |
| 11725964_a_at | BC005256.1 | | CAV2 | | | | 7.1105 | | 0.00821 | |
| 11753810_a_at | AF043341.1 | | CCL5 | | | | 5.5359 | | 0.01046 | |
| 11720007_a_at | NM_024636.2 | | STEAP4 | | | | 7.9087 | | 0.00262 | |
| 11715793_a_at | NM_001615.3 | | ACTG2 | | | | 8.3749 | | 0.02267 | |
| 11746088_a_at | DB350079 | | IFI44 | | | | 8.5172 | | 0.00037 | |
| 11718986_a_at | NM_022873.2 | | IFI6 | | | | 11.2017 | | 0.0467 | |
| 11757480_x_at | AW960588 | | IFI27 | | | | 37.8755 | | 0.02231 | |
| 11762785_x_at | AK301175.1 | | ACTG1 | | | | 0.436 | | 0.01509 | |
| 11724716_a_at | NM_001123385.1 | | BCOR | | | | 0.5 | | 0.0213 | |
| 11732318_a_at | BC032606.2 | | KHDRBS3 | | | | 0.4974 | | 0.00226 | |
| 11753500_x_at | AY189939.1 | | HOMER1 | | | | 0.4676 | | 0.00758 | |
| 11727532_a_at | BC064484.1 | | FEZ2 | | | | 0.4945 | | 0.03798 | |
| 11715745_a_at | NM_020444.3 | | KIAA1191 | | | | 0.4919 | | 0.01203 | |
| 11758750_x_at | BC083508.1 | | YWHAZ | | | | 0.4889 | | 0.05734 | |
| 11739094_a_at | L01639.1 | | CXCR4 | | | | 0.4873 | | 0.00254 | |
| 11717935_a_at | NM_024896.2 | | ERMP1 | | | | 0.4867 | | 0.04502 | |
| 11723390_a_at | NM_003389.3 | | CORO2A | | | | 0.4849 | | 0.00261 | |
| 11734548_a_at | NM_000358.2 | | TGFBI | | | | 0.4843 | | 0.02662 | |
| 11755665_x_at | BC063427.1 | | VARS2 | | | | 0.4826 | | 0.0105 | |
| 11758629_s_at | AA740387 | | SPTBN1 | | | | 0.4817 | | 0.01177 | |
| 11722160_a_at | NM_001001555.2 | | GRB10 | | | | 0.4802 | | 0.04956 | |
| 11736442_a_at | BC110067.1 | | SPINK7 | | | | 0.4712 | | 0.0434 | |
| 11740781_a_at | NM_153464.2 | | ILF3 | | | | 0.4707 | | 0.02327 | |
| 11763168_s_at | BC117379.1 | | ALG13 | | | | 0.4676 | | 0.00336 | |
| 11760221_at | AL050391.1 | | CASP4 | | | | 0.4676 | | 0.01797 | |
| 11740157_at | BC022407.1 | | SH2D1B | | | | 0.4657 | | 0.01394 | |
| 11743295_a_at | AK307690.1 | | CENPF | | | | 0.4647 | | 0.02316 | |
| 11719864_a_at | NM_001031628.1 | | SMAGP | | | | 0.4609 | | 0.04405 | |
| 11755252_a_at | AK091761.1 | | AMIGO2 | | | | 0.4603 | | 0.01741 | |
| 11721562_a_at | NM_006589.2 | | FAM189B | | | | 0.4578 | | 0.00545 | |
| 11744915_a_at | BQ009101 | | NEGR1 | | | | 0.4521 | | 0.0639 | |
| 11739787_a_at | NM_001007139.4 | | IGF2 | | | | 0.4496 | | 0.02602 | |
| 11729258_at | BC034991.2 | | MNS1 | | | | 0.4488 | | 0.03829 | |
| 11725842_a_at | NM_025049.2 | | PIF1 | | | | 0.447 | | 0.04497 | |
| 11717516_s_at | NM_025202.2 | | EFHD1 | | | | 0.442 | | 0.04177 | |
| 11758028_s_at | BQ184392 | | FOXQ1 | | | | 0.4409 | | 0.01467 | |
| 11753234_a_at | AK303598.1 | | TAMM41 | | | | 0.438 | | 0.04838 | |
| 11758200_x_at | BM009647 | | CKS1B | | | | 0.4306 | | 0.03399 | |
| 11718067_at | AK291688.1 | | MYO1E | | | | 0.4285 | | 0.01409 | |
| 11756714_a_at | CR600533.1 | | PPP2R2A | | | | 0.4243 | | 0.01048 | |
| 11721650_at | NM_002638.3 | | PI3 | | | | 0.4192 | | 0.03206 | |
| 11716433_s_at | NM_001037582.2 | | SCD5 | | | | 0.4157 | | 0.05694 | |
| 11727537_at | NM_001870.2 | | CPA3 | | | | 0.4103 | | 0.016 | |
| 11748808_a_at | AK298716.1 | | ARID3B | | | | 0.4086 | | 0.06477 | |
| 11745652_s_at | M26481.1 | | EPCAM | | | | 0.4196 | | 0.00785 | |
| 11715394_s_at | NM_004356.3 | | CD81 | | | | 0.395 | | 0.00179 | |
| 11759022_s_at | BX436694 | | ELK3 | | | | 0.3857 | | 0.05232 | |
| 11754544_s_at | AK096220.1 | | ZDHHC11 | | | | 0.3849 | | 0.0022 | |
| 11726838_s_at | NM_173690.3 | | SCAI | | | | 0.3767 | | 0.01443 | |
| 11755110_a_at | AK126745.1 | | LRIG1 | | | | 0.3673 | | 0.06512 | |
| 11760294_a_at | AB240569.1 | | CD55 | | | | 0.3665 | | 0.00996 | |
| 11744034_a_at | AL571788 | | VASP | | | | 0.3602 | | 0.01081 | |
| 11727645_a_at | NM_001105078.2 | | MECOM | | | | 0.3359 | | 0.05636 | |
| 11744030_a_at | AI565078 | | MMP7 | | | | 0.3194 | | 0.03051 | |
| 11717863_a_at | NM_004419.3 | | DUSP5 | | | | 0.3174 | | 0.00941 | |
| 11722215_at | NM_019896.2 | | POLE4 | | | | 0.3168 | | 0.00795 | |
| 11717212_a_at | NM_002499.2 | | NEO1 | | | | 0.3149 | | 0.00054 | |
| 11743629_at | BG200487 | | IRS1 | | | | 0.2811 | | 0.00372 | |
| 11744162_a_at | DB361302 | | DUSP4 | | | | 0.2231 | | 0.0158 | |
| 11717473_s_at | NM_000596.2 | | IGFBP1 | | | | 0.2119 | | 0.00059 | |
| 11733091_a_at | NM_004616.2 | | TSPAN8 | | | | 0.2045 | | 0.0058 | |
| 11733042_at | DA830350 | | SLC7A2 | | | | 0.2043 | | 0.01511 | |
| 11719222_at | NM_003122.3 | | SPINK1 | | | | 0.1739 | | 0.00325 | |
| 11725280_a_at | NM_020226.3 | | PRDM8 | | | | 0.1676 | | 0.00951 | |
| 11732111_a_at | NM_014452.3 | | TNFRSF21 | | | | 0.1259 | | 0.01843 | |
| 11757033_a_at | DA534357 | | IL13RA2 | | | | 0.1001 | | 0.0341 | |
| **S1_2 Table. List of genes that are regulated by PYCARD-AS1 knockdown (shAS-2 versus shNC)** | | | | | | | | | | |
| **ProbeSetID** | **Target transcript ID** | | **Gene symbol** | | | | **Fold change** | | **p value** | |
| 11716384_at | NM_002982.3 | | CCL2 | | | | 2.0808 | | 0.010654794 | |
| 11740613_a_at | BC009006.1 | | TMEM30A | | | | 2.34 | | 0.046166258 | |
| 11752384_s_at | AK299396.1 | | COL6A1 | | | | 1.9249 | | 0.025523357 | |
| 11729020_at | NM_194250.1 | | ZNF804A | | | | 2.101 | | 0.004704168 | |
| 11749510_a_at | AK304187.1 | | SAMHD1 | | | | 2.0411 | | 0.046369206 | |
| 11744829_s_at | AK296822.1 | | HLA-E | | | | 2.0098 | | 0.023648638 | |
| 11748489_s_at | AK301821.1 | | MAGED2 | | | | 2.1537 | | 0.011065667 | |
| 11757396_s_at | BU675268 | | ANXA8 | | | | 2 | | 0.019525561 | |
| 11751698_a_at | AK301560.1 | | EAF2 | | | | 2.174 | | 0.008930547 | |
| 11728022_a_at | NM_018004.1 | | TMEM45A | | | | 2.0007 | | 0.038489679 | |
| 11728792_x_at | NM_013302.3 | | EEF2K | | | | 2.0987 | | 0.034662363 | |
| 11725861_a_at | NM_001080416.2 | | MYBL1 | | | | 2.0707 | | 0.026016935 | |
| 11757942_s_at | BM998048 | | UTRN | | | | 2.4857 | | 0.001436742 | |
| 11723181_a_at | NM_002747.3 | | MAPK4 | | | | 2.1481 | | 0.002695648 | |
| 11720028_x_at | BC014514.1 | | LDLR | | | | 2.0295 | | 0.018462604 | |
| 11724917_at | AK023165.1 | | TMTC3 | | | | 2.633 | | 0.044020096 | |
| 11716876_a_at | NM_003313.3 | | TSTA3 | | | | 2.2911 | | 0.029838522 | |
| 11730614_a_at | NM_003038.3 | | SLC1A4 | | | | 2.0603 | | 0.025532482 | |
| 11716413_x_at | NM_175617.3 | | MT1E | | | | 2.1031 | | 0.011547105 | |
| 11728451_a_at | NM_013363.2 | | PCOLCE2 | | | | 1.855 | | 0.016227414 | |
| 11719628_a_at | NM_001135565.1 | | HDHD1 | | | | 2.1767 | | 0.026199139 | |
| 11745300_a_at | BX647203.1 | | DNAJC6 | | | | 2.0597 | | 0.021450325 | |
| 11717743_a_at | NM_007274.3 | | ACOT7 | | | | 2.148 | | 0.033076946 | |
| 11727440_at | BC007522.1 | | LOXL4 | | | | 2.2816 | | 0.012124079 | |
| 11715536_a_at | NM_002168.2 | | IDH2 | | | | 2.3786 | | 0.003931601 | |
| 11756141_s_at | DW454053 | | KDELR2 | | | | 2.1048 | | 0.001525932 | |
| 11722355_s_at | NM_001343.2 | | DAB2 | | | | 2.1537 | | 0.026453876 | |
| 11746440_s_at | AK293345.1 | | GLO1 | | | | 2.0037 | | 0.038659424 | |
| 11721864_s_at | NM_207113.1 | | SLC37A3 | | | | 2.0128 | | 0.001310858 | |
| 11728300_at | NM_057749.1 | | CCNE2 | | | | 2.0338 | | 0.014034144 | |
| 11720087_a_at | NM_005013.2 | | NUCB2 | | | | 2.3405 | | 0.013818281 | |
| 11735140_at | NM_003811.3 | | TNFSF9 | | | | 2.0187 | | 0.016710483 | |
| 11726774_a_at | NM_005060.3 | | RORC | | | | 2.0361 | | 0.005361737 | |
| 11736061_a_at | NM_002639.4 | | SERPINB5 | | | | 2.2841 | | 0.025235639 | |
| 11743626_s_at | CA450422 | | LGR4 | | | | 2.0572 | | 0.010139902 | |
| 11725804_a_at | NM_001100420.1 | | C21orf91 | | | | 2.1242 | | 0.002473717 | |
| 11719075_s_at | NM_022912.2 | | REEP1 | | | | 1.9839 | | 0.013299627 | |
| 11733085_a_at | NM_001976.3 | | ENO3 | | | | 2.211 | | 0.010816749 | |
| 11722093_a_at | NM_000943.4 | | PPIC | | | | 2.0986 | | 0.031534237 | |
| 11757504_a_at | BQ188977 | | DDAH1 | | | | 2.1838 | | 0.035453876 | |
| 11720780_a_at | NM_020775.2 | | KIAA1324 | | | | 2.1258 | | 0.03135866 | |
| 11722152_s_at | NM_004836.4 | | EIF2AK3 | | | | 2.0184 | | 0.046825701 | |
| 11725951_at | AF013591.1 | | RIOK3 | | | | 2.1004 | | 0.045451608 | |
| 11755284_s_at | AK093487.1 | | TSPAN13 | | | | 2.1961 | | 0.034824923 | |
| 11716167_a_at | NM_001144925.1 | | MX1 | | | | 2.176 | | 0.007942008 | |
| 11743352_s_at | AB023194.1 | | COBLL1 | | | | 2.118 | | 0.046575654 | |
| 11715557_at | CR597598.1 | | FLJ23867 | | | | 2 | | 0.036871072 | |
| 11725198_at | NM_000575.3 | | IL1A | | | | 2.137 | | 0.02687443 | |
| 11755620_a_at | BC025774.1 | | HYAL1 | | | | 2.0461 | | 0.008660399 | |
| 11726479_a_at | NM_002463.1 | | MX2 | | | | 2.1053 | | 0.022256798 | |
| 11724117_x_at | AI038997 | | SAMD9L | | | | 2.4954 | | 0.006145406 | |
| 11739302_a_at | AB012911.1 | | FZD6 | | | | 1.9705 | | 0.008375189 | |
| 11726113_a_at | NM_052943.3 | | FAM46B | | | | 2.3172 | | 0.026321922 | |
| 11754948_a_at | AK096462.1 | | HECTD3 | | | | 2.0413 | | 0.019502405 | |
| 11730719_x_at | AF087876.1 | | AP1S2 | | | | 2.2316 | | 0.029191829 | |
| 11724488_at | NM_020299.4 | | AKR1B10 | | | | 2.114 | | 0.010406316 | |
| 11744178_a_at | CR592313.1 | | C19orf66 | | | | 2.1145 | | 0.080465944 | |
| 11729238_a_at | NM_001321.1 | | CSRP2 | | | | 2.4054 | | 0.010448815 | |
| 11722879_a_at | NM_181674.2 | | PPP2R2B | | | | 2.3053 | | 0.043324133 | |
| 11720669_at | NM_003884.4 | | KAT2B | | | | 2.1592 | | 0.029962108 | |
| 11732636_at | NM_024746.3 | | HHIPL2 | | | | 2.4574 | | 0.015130136 | |
| 11723669_s_at | NM_006379.2 | | SEMA3C | | | | 2.1532 | | 0.03898276 | |
| 11747808_a_at | AK301830.1 | | WIPI1 | | | | 2.353 | | 0.029983431 | |
| 11737299_at | NM_152304.1 | | RAB42 | | | | 2.299 | | 0.030437507 | |
| 11723151_s_at | NM_024728.1 | | SUGCT | | | | 2.4966 | | 0.091016148 | |
| 11719314_a_at | NM_017881.2 | | NMRK1 | | | | 2.3293 | | 0.007145912 | |
| 11760254_at | DB350079 | | IFI44 | | | | 2.3717 | | 0.002112274 | |
| 11757702_s_at | BF063817 | | DSC2 | | | | 2.1199 | | 0.036096742 | |
| 11739232_x_at | AA827918 | | RASSF2 | | | | 2.3462 | | 0.00066145 | |
| 11721010_a_at | NM_030792.6 | | GDPD5 | | | | 2.3717 | | 0.019063026 | |
| 11718179_a_at | AI928241 | | FERMT2 | | | | 2.1776 | | 0.034976838 | |
| 11723007_a_at | AB033080.2 | | CCPG1 | | | | 2.2006 | | 0.030038678 | |
| 11750531_a_at | NM_013258.3 | | PYCARD | | | | 2.5543 | | 0.032887362 | |
| 11758573_s_at | AI689215 | | MORC4 | | | | 2.3933 | | 0.007549187 | |
| 11723051_at | NM_005130.3 | | FGFBP1 | | | | 2.3725 | | 0.051810555 | |
| 11723234_at | AB116553.1 | | IFI44L | | | | 2.2393 | | 0.036322679 | |
| 11718332_a_at | AK293677.1 | | TSPAN3 | | | | 2.4308 | | 0.052286523 | |
| 11722909_a_at | NM_178831.5 | | GATS | | | | 2.4772 | | 0.031044153 | |
| 11741555_s_at | NM_001127605.1 | | LIPA | | | | 2.2387 | | 0.065600464 | |
| 11721897_a_at | NM_024119.2 | | DHX58 | | | | 2.2981 | | 0.007011154 | |
| 11741704_x_at | NM_003526.2 | | HIST1H2BC | | | | 2.4774 | | 0.0206706 | |
| 11738185_s_at | NM_001029851.1 | | PDE8B | | | | 2.441 | | 0.020137402 | |
| 11717644_a_at | NM_001136557.1 | | GPR107 | | | | 2.4412 | | 0.007544479 | |
| 11716895_s_at | NM_005101.3 | | ISG15 | | | | 2.4767 | | 0.014705429 | |
| 11749922_x_at | BC065829.1 | | TAGLN | | | | 1.8292 | | 0.033499468 | |
| 11726770_x_at | BC073156.1 | | XAF1 | | | | 2.8278 | | 0.003279456 | |
| 11731831_x_at | NM_138413.3 | | HOGA1 | | | | 2.5541 | | 0.038863574 | |
| 11722503_at | NM_002214.2 | | ITGB8 | | | | 2.3573 | | 0.048030889 | |
| 11761536_a_at | AK297048.1 | | TM2D3 | | | | 2.4836 | | 0.000727742 | |
| 11719833_at | NM_005797.2 | | MPZL2 | | | | 2.2555 | | 0.038726289 | |
| 11727553_a_at | BC093027.1 | | PKIB | | | | 2.5552 | | 0.047415978 | |
| 11763173_s_at | CA417761 | | PGM3 | | | | 2.5535 | | 0.011812391 | |
| 11724399_a_at | NM_001012967.1 | | DDX60L | | | | 2.6909 | | 0.002170577 | |
| 11715576_at | AF136373.1 | | RAC1 | | | | 2.5244 | | 0.000220547 | |
| 11740602_s_at | NM_001145646.1 | | APH1B | | | | 2.4669 | | 0.002224376 | |
| 11731137_a_at | AF328296.1 | | MYPN | | | | 2.4353 | | 0.048902234 | |
| 11715670_a_at | NM_003641.3 | | IFITM1 | | | | 2.5137 | | 0.000455718 | |
| 11755762_a_at | BC144510.1 | | TMEM2 | | | | 2.8231 | | 0.014033124 | |
| 11719764_s_at | NM_212503.1 | | CDK18 | | | | 2.6118 | | 0.002135382 | |
| 11725498_a_at | NM_032256.1 | | TMEM117 | | | | 2.643 | | 0.006516023 | |
| 11718231_x_at | NM_018950.2 | | HLA-F | | | | 2.6489 | | 0.000366732 | |
| 11748907_a_at | AK293357.1 | | RARRES3 | | | | 2.6873 | | 0.023881005 | |
| 11721874_at | BC032839.2 | | IFIT2 | | | | 2.7974 | | 0.004612212 | |
| 11729243_s_at | U03272.1 | | FBN2 | | | | 2.5986 | | 0.046087749 | |
| 11726364_x_at | NM_003733.2 | | OASL | | | | 2.841 | | 0.044565887 | |
| 11718916_a_at | NM_004031.2 | | IRF7 | | | | 2.4541 | | 0.045297358 | |
| 11719491_a_at | NM_005533.3 | | IFI35 | | | | 3.0353 | | 0.036322679 | |
| 11717575_at | NM_005398.4 | | PPP1R3C | | | | 2.8729 | | 0.008247379 | |
| 11755147_s_at | BX640607.1 | | STAT2 | | | | 2.7511 | | 0.038081865 | |
| 11737847_a_at | NM_001531.1 | | MR1 | | | | 2.5913 | | 0.036237737 | |
| 11720704_s_at | NM_001003395.1 | | TPD52L1 | | | | 2.7416 | | 0.002891448 | |
| 11718435_a_at | NM_001033047.1 | | NPNT | | | | 2.6497 | | 0.048486728 | |
| 11745624_s_at | BC142661.1 | | INADL | | | | 2.8459 | | 0.046662415 | |
| 11723128_a_at | NM_017912.3 | | HERC6 | | | | 2.4873 | | 0.025939538 | |
| 11741315_a_at | NM_001128305.1 | | PLSCR4 | | | | 2.9023 | | 0.022106953 | |
| 11755819_a_at | AK125989.1 | | DDX58 | | | | 2.8142 | | 0.000355171 | |
| 11742232_s_at | NM_001040020.1 | | FAM3C | | | | 2.6812 | | 0.044003354 | |
| 11735987_at | NM_032935.2 | | MT4 | | | | 3.131 | | 0.031187174 | |
| 11721994_s_at | NM_198183.1 | | UBE2L6 | | | | 3.2194 | | 0.003682071 | |
| 11757430_s_at | BX373810 | | TMED5 | | | | 2.9261 | | 0.023568503 | |
| 11721335_x_at | NM_182943.2 | | PLOD2 | | | | 3.0019 | | 0.006793983 | |
| 11728954_a_at | NM_000899.3 | | KITLG | | | | 3.122 | | 0.013811191 | |
| 11744236_a_at | AW150758 | | DDX60 | | | | 2.9383 | | 0.012559962 | |
| 11724346_a_at | NM_022168.2 | | IFIH1 | | | | 2.9754 | | 0.013337828 | |
| 11725999_s_at | AK290978.1 | | FAS | | | | 2.6725 | | 0.025589747 | |
| 11743353_s_at | BM975358 | | STAT1 | | | | 2.8004 | | 0.027527654 | |
| 11746798_x_at | AK298939.1 | | SDC2 | | | | 2.7991 | | 0.026179956 | |
| 11757650_s_at | BM997052 | | FN1 | | | | 2.3217 | | 0.01619783 | |
| 11719268_at | NM_003280.2 | | TNNC1 | | | | 3.2678 | | 0.085246084 | |
| 11717275_s_at | BC008760.2 | | COL5A1 | | | | 2.9353 | | 0.033450108 | |
| 11723329_a_at | NM_001127662.1 | | GSN | | | | 3.2282 | | 0.003913829 | |
| 11728764_a_at | NM_030916.2 | | PVRL4 | | | | 3.4701 | | 0.042651694 | |
| 11724463_a_at | NM_001005340.1 | | GPNMB | | | | 3.2914 | | 0.012938508 | |
| 11749773_x_at | AK303893.1 | | GBP1 | | | | 3.381 | | 0.024142131 | |
| 11723854_at | NM_017654.2 | | SAMD9 | | | | 3.7176 | | 0.000859872 | |
| 11724885_at | NM_004669.2 | | CLIC3 | | | | 3.5004 | | 0.064524028 | |
| 11755400_s_at | BX647113.1 | | STK39 | | | | 3.1309 | | 0.024572565 | |
| 11724378_s_at | BC090931.1 | | PAG1 | | | | 3.5321 | | 0.008157162 | |
| 11721349_s_at | NM_017414.3 | | LOC102725067 | | | | 3.639 | | 0.011267723 | |
| 11731682_at | NM_001252.3 | | CD70 | | | | 3.5389 | | 0.067644183 | |
| 11715388_s_at | NM_078467.1 | | CDKN1A | | | | 3.4414 | | 0.007542122 | |
| 11731657_s_at | NM_001079874.1 | | VAV3 | | | | 3.27 | | 0.007053643 | |
| 11731819_a_at | NM_017752.2 | | TBC1D8B | | | | 3.0633 | | 0.002195248 | |
| 11733213_x_at | NM_144975.3 | | SLFN5 | | | | 3.4679 | | 0.020513689 | |
| 11741581_s_at | NM_130798.1 | | SNAP23 | | | | 3.6165 | | 0.019204289 | |
| 11722244_at | NM_020665.3 | | TMEM27 | | | | 3.6395 | | 0.006978417 | |
| 11744434_a_at | AF307339.1 | | PARP9 | | | | 3.9528 | | 0.032525734 | |
| 11715988_x_at | NM_001002027.1 | | ATP5G1 | | | | 3.9389 | | 0.014057231 | |
| 11751651_s_at | AK300181.1 | | PLSCR1 | | | | 3.8101 | | 0.003685387 | |
| 11724538_a_at | BC021281.2 | | ABCG2 | | | | 3.8164 | | 0.008986523 | |
| 11755446_s_at | AK222631.1 | | ANKH | | | | 3.494 | | 0.026733095 | |
| 11716308_s_at | NM_024843.3 | | CYBRD1 | | | | 3.5875 | | 0.036102079 | |
| 11755587_a_at | X82200.1 | | TRIM22 | | | | 4.4967 | | 0.01430816 | |
| 11725188_a_at | BX640971.1 | | GPR126 | | | | 4.4549 | | 0.007488849 | |
| 11715620_a_at | NM_001885.1 | | CRYAB | | | | 4.3152 | | 0.024832019 | |
| 11724325_a_at | NM_014857.3 | | RABGAP1L | | | | 4.3067 | | 0.004070764 | |
| 11720209_at | NM_006084.4 | | IRF9 | | | | 4.1597 | | 2.21E-05 | |
| 11753554_a_at | BC128163.1 | | SPINK13 | | | | 4.2858 | | 0.054292575 | |
| 11722963_a_at | NM_016429.2 | | COPZ2 | | | | 4.3289 | | 0.02422128 | |
| 11724256_s_at | NM_016816.2 | | OAS1 | | | | 4.6749 | | 0.000600599 | |
| 11764077_s_at | AW150861 | | EDIL3 | | | | 4.4276 | | 0.003988196 | |
| 11730370_a_at | BM992839 | | EIF4E3 | | | | 4.9055 | | 0.001116899 | |
| 11719447_s_at | BC073163.1 | | GBP2 | | | | 4.1169 | | 0.036370007 | |
| 11721902_a_at | NM_005025.4 | | SERPINI1 | | | | 5.1551 | | 0.001923594 | |
| 11756820_a_at | AK308688.1 | | IFIT1 | | | | 6.307 | | 0.028999464 | |
| 11726201_a_at | NM_016817.2 | | OAS2 | | | | 5.807 | | 0.026514721 | |
| 11731606_at | NM_001112706.2 | | SCIN | | | | 6.5056 | | 0.002847154 | |
| 11733025_at | AB026833.1 | | CLCA2 | | | | 6.7994 | | 0.042246595 | |
| 11725964_a_at | BC005256.1 | | CAV2 | | | | 7.1395 | | 0.006692549 | |
| 11753810_a_at | AF043341.1 | | CCL5 | | | | 7.9068 | | 0.028650031 | |
| 11720007_a_at | NM_024636.2 | | STEAP4 | | | | 7.9518 | | 0.001586453 | |
| 11715793_a_at | NM_001615.3 | | ACTG2 | | | | 8.3952 | | 0.00770481 | |
| 11746088_a_at | DB350079 | | IFI44 | | | | 9.1336 | | 0.003994219 | |
| 11718986_a_at | NM_022873.2 | | IFI6 | | | | 10.4914 | | 0.005697855 | |
| 11757480_x_at | AW960588 | | IFI27 | | | | 41.4292 | | 0.005398834 | |
| 11762785_x_at | AK301175.1 | | ACTG1 | | | | 0.4172 | | 0.01385176 | |
| 11724716_a_at | NM_001123385.1 | | BCOR | | | | 0.457 | | 0.005292102 | |
| 11732318_a_at | BC032606.2 | | KHDRBS3 | | | | 0.4327 | | 0.024873009 | |
| 11753500_x_at | AY189939.1 | | HOMER1 | | | | 0.4421 | | 0.00708422 | |
| 11727532_a_at | BC064484.1 | | FEZ2 | | | | 0.4976 | | 0.055329771 | |
| 11715745_a_at | NM_020444.3 | | KIAA1191 | | | | 0.486 | | 0.000887038 | |
| 11758750_x_at | BC083508.1 | | YWHAZ | | | | 0.4774 | | 0.026707701 | |
| 11739094_a_at | L01639.1 | | CXCR4 | | | | 0.3372 | | 0.003492159 | |
| 11717935_a_at | NM_024896.2 | | ERMP1 | | | | 0.4678 | | 0.024472985 | |
| 11723390_a_at | NM_003389.3 | | CORO2A | | | | 0.495 | | 0.021441456 | |
| 11734548_a_at | NM_000358.2 | | TGFBI | | | | 0.5 | | 0.014187151 | |
| 11755665_x_at | BC063427.1 | | VARS2 | | | | 0.4755 | | 0.0075513 | |
| 11758629_s_at | AA740387 | | SPTBN1 | | | | 0.4606 | | 0.02878538 | |
| 11722160_a_at | NM_001001555.2 | | GRB10 | | | | 0.465 | | 0.03379144 | |
| 11736442_a_at | BC110067.1 | | SPINK7 | | | | 0.3889 | | 0.021655221 | |
| 11740781_a_at | NM_153464.2 | | ILF3 | | | | 0.3643 | | 0.028883416 | |
| 11763168_s_at | BC117379.1 | | ALG13 | | | | 0.4584 | | 0.041976075 | |
| 11760221_at | AL050391.1 | | CASP4 | | | | 0.4951 | | 0.046567968 | |
| 11740157_at | BC022407.1 | | SH2D1B | | | | 0.3648 | | 0.047947185 | |
| 11743295_a_at | AK307690.1 | | CENPF | | | | 0.5346 | | 0.013077949 | |
| 11719864_a_at | NM_001031628.1 | | SMAGP | | | | 0.4326 | | 0.002971174 | |
| 11755252_a_at | AK091761.1 | | AMIGO2 | | | | 0.4547 | | 0.003620015 | |
| 11721562_a_at | NM_006589.2 | | FAM189B | | | | 0.4628 | | 0.005332003 | |
| 11744915_a_at | BQ009101 | | NEGR1 | | | | 0.3642 | | 0.012472519 | |
| 11739787_a_at | NM_001007139.4 | | IGF2 | | | | 0.3807 | | 0.042731254 | |
| 11729258_at | BC034991.2 | | MNS1 | | | | 0.4682 | | 0.041330688 | |
| 11725842_a_at | NM_025049.2 | | PIF1 | | | | 0.4564 | | 0.029968032 | |
| 11717516_s_at | NM_025202.2 | | EFHD1 | | | | 0.4847 | | 0.00716488 | |
| 11758028_s_at | BQ184392 | | FOXQ1 | | | | 0.3692 | | 0.010474847 | |
| 11753234_a_at | AK303598.1 | | TAMM41 | | | | 0.4833 | | 0.035856411 | |
| 11758200_x_at | BM009647 | | CKS1B | | | | 0.4384 | | 0.00419307 | |
| 11718067_at | AK291688.1 | | MYO1E | | | | 0.4952 | | 0.019572949 | |
| 11756714_a_at | CR600533.1 | | PPP2R2A | | | | 0.4038 | | 0.003600784 | |
| 11721650_at | NM_002638.3 | | PI3 | | | | 0.4319 | | 0.029140181 | |
| 11716433_s_at | NM_001037582.2 | | SCD5 | | | | 0.3437 | | 0.022464943 | |
| 11727537_at | NM_001870.2 | | CPA3 | | | | 0.4317 | | 0.002659209 | |
| 11748808_a_at | AK298716.1 | | ARID3B | | | | 0.3605 | | 0.042067585 | |
| 11745652_s_at | M26481.1 | | EPCAM | | | | 0.3986 | | 0.006520788 | |
| 11715394_s_at | NM_004356.3 | | CD81 | | | | 0.3875 | | 0.004097599 | |
| 11759022_s_at | BX436694 | | ELK3 | | | | 0.4219 | | 0.017270632 | |
| 11754544_s_at | AK096220.1 | | ZDHHC11 | | | | 0.4363 | | 0.077149648 | |
| 11726838_s_at | NM_173690.3 | | SCAI | | | | 0.3874 | | 0.046980837 | |
| 11755110_a_at | AK126745.1 | | LRIG1 | | | | 0.3441 | | 0.04740166 | |
| 11760294_a_at | AB240569.1 | | CD55 | | | | 0.3749 | | 0.010806848 | |
| 11744034_a_at | AL571788 | | VASP | | | | 0.3389 | | 0.032436112 | |
| 11727645_a_at | NM_001105078.2 | | MECOM | | | | 0.3614 | | 0.048958926 | |
| 11744030_a_at | AI565078 | | MMP7 | | | | 0.3482 | | 0.016012955 | |
| 11717863_a_at | NM_004419.3 | | DUSP5 | | | | 0.2951 | | 0.002733029 | |
| 11722215_at | NM_019896.2 | | POLE4 | | | | 0.346 | | 0.018761271 | |
| 11717212_a_at | NM_002499.2 | | NEO1 | | | | 0.3264 | | 0.007563195 | |
| 11743629_at | BG200487 | | IRS1 | | | | 0.2831 | | 0.019481964 | |
| 11744162_a_at | DB361302 | | DUSP4 | | | | 0.211 | | 0.025377545 | |
| 11717473_s_at | NM_000596.2 | | IGFBP1 | | | | 0.1862 | | 0.011004743 | |
| 11733091_a_at | NM_004616.2 | | TSPAN8 | | | | 0.2138 | | 0.04924771 | |
| 11733042_at | DA830350 | | SLC7A2 | | | | 0.3311 | | 0.0018462 | |
| 11719222_at | NM_003122.3 | | SPINK1 | | | | 0.1851 | | 0.001009238 | |
| 11725280_a_at | NM_020226.3 | | PRDM8 | | | | 0.1565 | | 0.023195836 | |
| 11732111_a_at | NM_014452.3 | | TNFRSF21 | | | | 0.1251 | | 0.011896027 | |
| 11757033_a_at | DA534357 | | IL13RA2 | | | | 0.1173 | | 0.024947346 | |
| **S1_3 Table. List of genes that are regulated by PYCARD-AS1 knockdown and reversed upon PYCARD knockdown (shAS-1/shS versus shAS-1)** | | | | | | | | | |  |
| **ProbeSetID** | **Target transcript ID** | | | **Gene symbol** | | **Fold change** | | **p value** | |  |
| 11715388_s_at | NM_078467.1 | | | CDKN1A | | 0.6115 | | 0.009219907 | |  |
| 11715394_s_at | NM_004356.3 | | | CD81 | | 2.2547 | | 0.003305537 | |  |
| 11715536_a_at | NM_002168.2 | | | IDH2 | | 0.6128 | | 0.026964147 | |  |
| 11715557_at | CR597598.1 | | | QSOX1 | | 0.5759 | | 0.022437406 | |  |
| 11715576_at | AF136373.1 | | | RAC1 | | 0.6839 | | 0.003119301 | |  |
| 11715620_a_at | NM_001885.1 | | | CRYAB | | 0.4724 | | 0.01109769 | |  |
| 11715670_a_at | NM_003641.3 | | | IFITM1 | | 0.5075 | | 0.00340636 | |  |
| 11715745_a_at | NM_020444.3 | | | KIAA1191 | | 1.7943 | | 0.003522865 | |  |
| 11715793_a_at | NM_001615.3 | | | ACTG2 | | 0.276 | | 0.002604239 | |  |
| 11716167_a_at | NM_001144925.1 | | | MX1 | | 0.5595 | | 0.006658175 | |  |
| 11716433_s_at | NM_001037582.2 | | | SCD5 | | 1.9826 | | 0.002091447 | |  |
| 11716876_a_at | NM_003313.3 | | | TSTA3 | | 0.6182 | | 0.039098656 | |  |
| 11716895_s_at | NM_005101.3 | | | ISG15 | | 0.4718 | | 0.04312022 | |  |
| 11717212_a_at | NM_002499.2 | | | NEO1 | | 1.6475 | | 0.004736273 | |  |
| 11717473_s_at | NM_000596.2 | | | IGFBP1 | | 3.9761 | | 0.001377401 | |  |
| 11717935_a_at | NM_024896.2 | | | ERMP1 | | 1.7887 | | 0.073723244 | |  |
| 11718067_at | AK291688.1 | | | MYO1E | | 1.8985 | | 0.028450092 | |  |
| 11718231_x_at | NM_018950.2 | | | HLA-F | | 0.6047 | | 0.015929853 | |  |
| 11718916_a_at | NM_004031.2 | | | IRF7 | | 0.4672 | | 0.037355413 | |  |
| 11718986_a_at | NM_022873.2 | | | IFI6 | | 0.3131 | | 0.012141065 | |  |
| 11719222_at | NM_003122.3 | | | SPINK1 | | 2.5217 | | 0.013684662 | |  |
| 11719268_at | NM_003280.2 | | | TNNC1 | | 0.605 | | 0.028375056 | |  |
| 11719447_s_at | BC073163.1 | | | GBP2 | | 0.4406 | | 0.011570523 | |  |
| 11719491_a_at | NM_005533.3 | | | IFI35 | | 0.5633 | | 0.043895195 | |  |
| 11719764_s_at | NM_212503.1 | | | CDK18 | | 0.6619 | | 0.045208137 | |  |
| 11719833_at | NM_005797.2 | | | MPZL2 | | 0.5699 | | 0.021304638 | |  |
| 11720007_a_at | NM_024636.2 | | | STEAP4 | | 0.66 | | 0.01366026 | |  |
| 11720028_x_at | BC014514.1 | | | LDLR | | 0.5672 | | 0.045241556 | |  |
| 11720209_at | NM_006084.4 | | | IRF9 | | 0.5276 | | 0.037355413 | |  |
| 11720669_at | NM_003884.4 | | | KAT2B | | 0.6651 | | 0.017535748 | |  |
| 11721562_a_at | NM_006589.2 | | | FAM189B | | 1.945 | | 0.008382433 | |  |
| 11721864_s_at | NM_207113.1 | | | SLC37A3 | | 0.6555 | | 0.043037515 | |  |
| 11721874_at | BC032839.2 | | | IFIT2 | | 0.6249 | | 0.029389068 | |  |
| 11721897_a_at | NM_024119.2 | | | DHX58 | | 0.5838 | | 0.00127431 | |  |
| 11721994_s_at | NM_198183.1 | | | UBE2L6 | | 0.5611 | | 0.01932439 | |  |
| 11722093_a_at | NM_000943.4 | | | PPIC | | 0.6732 | | 0.010910279 | |  |
| 11722215_at | NM_019896.2 | | | POLE4 | | 2.6153 | | 0.000184986 | |  |
| 11722244_at | NM_020665.3 | | | TMEM27 | | 0.6782 | | 0.020447635 | |  |
| 11722503_at | NM_002214.2 | | | ITGB8 | | 0.6563 | | 0.012237914 | |  |
| 11722909_a_at | NM_178831.5 | | | GATS | | 0.67 | | 0.005762578 | |  |
| 11722963_a_at | NM_016429.2 | | | COPZ2 | | 0.5269 | | 0.010880574 | |  |
| 11723051_at | NM_005130.3 | | | FGFBP1 | | 0.5428 | | 0.035588311 | |  |
| 11723128_a_at | NM_017912.3 | | | HERC6 | | 0.5505 | | 0.009379267 | |  |
| 11723234_at | AB116553.1 | | | IFI44L | | 0.5017 | | 0.012141065 | |  |
| 11723390_a_at | NM_003389.3 | | | CORO2A | | 2.1348 | | 0.042202641 | |  |
| 11723854_at | NM_017654.2 | | | SAMD9 | | 0.6525 | | 0.009019306 | |  |
| 11724117_x_at | AI038997 | | | SAMD9L | | 0.6525 | | 0.009019306 | |  |
| 11724256_s_at | NM_016816.2 | | | OAS1 | | 0.3916 | | 0.007205739 | |  |
| 11724325_a_at | NM_014857.3 | | | RABGAP1L | | 0.5718 | | 0.018851796 | |  |
| 11724346_a_at | NM_022168.2 | | | IFIH1 | | 0.3554 | | 0.002881868 | |  |
| 11724378_s_at | BC090931.1 | | | PAG1 | | 0.4705 | | 0.054832359 | |  |
| 11724399_a_at | NM_001012967.1 | | | DDX60L | | 0.667 | | 0.010377886 | |  |
| 11724463_a_at | NM_001005340.1 | | | GPNMB | | 0.5779 | | 0.008308198 | |  |
| 11724538_a_at | BC021281.2 | | | ABCG2 | | 0.5138 | | 0.014302895 | |  |
| 11724885_at | NM_004669.2 | | | CLIC3 | | 0.4761 | | 0.026692969 | |  |
| 11725188_a_at | BX640971.1 | | | GPR126 | | 0.6697 | | 0.009798261 | |  |
| 11725280_a_at | NM_020226.3 | | | PRDM8 | | 1.7609 | | 0.016651891 | |  |
| 11725951_at | AF013591.1 | | | RIOK3 | | 0.6707 | | 0.00868653 | |  |
| 11725964_a_at | BC005256.1 | | | CAV2 | | 0.6208 | | 0.005247891 | |  |
| 11725999_s_at | AK290978.1 | | | FAS | | 0.6903 | | 0.065208872 | |  |
| 11726113_a_at | NM_052943.3 | | | FAM46B | | 0.658 | | 0.020918701 | |  |
| 11726201_a_at | NM_016817.2 | | | OAS2 | | 0.2911 | | 0.040046803 | |  |
| 11726364_x_at | NM_003733.2 | | | OASL | | 0.4635 | | 0.010668332 | |  |
| 11726479_a_at | NM_002463.1 | | | MX2 | | 0.5321 | | 0.01215223 | |  |
| 11726770_x_at | BC073156.1 | | | XAF1 | | 0.7392 | | 0.004494722 | |  |
| 11726838_s_at | NM_173690.3 | | | SCAI | | 2.1679 | | 0.01935873 | |  |
| 11727440_at | BC007522.1 | | | LOXL4 | | 0.6944 | | 0.008996878 | |  |
| 11727537_at | NM_001870.2 | | | CPA3 | | 2.89 | | 0.051163402 | |  |
| 11727645_a_at | NM_001105078.2 | | | MECOM | | 2.2775 | | 0.042355894 | |  |
| 11728300_at | NM_057749.1 | | | CCNE2 | | 0.6395 | | 0.035311914 | |  |
| 11729258_at | BC034991.2 | | | MNS1 | | 1.8344 | | 0.010253409 | |  |
| 11730370_a_at | BM992839 | | | EIF4E3 | | 0.3685 | | 0.044818818 | |  |
| 11731137_a_at | AF328296.1 | | | MYPN | | 0.6989 | | 0.009680008 | |  |
| 11731606_at | NM_001112706.2 | | | SCIN | | 0.4209 | | 0.002770128 | |  |
| 11731657_s_at | NM_001079874.1 | | | VAV3 | | 0.5081 | | 0.014448167 | |  |
| 11731819_a_at | NM_017752.2 | | | TBC1D8B | | 0.6827 | | 0.019243861 | |  |
| 11731831_x_at | NM_138413.3 | | | HOGA1 | | 0.5773 | | 0.020299709 | |  |
| 11732111_a_at | NM_014452.3 | | | TNFRSF21 | | 3.777 | | 0.005556848 | |  |
| 11732318_a_at | BC032606.2 | | | KHDRBS3 | | 2.0398 | | 0.027202466 | |  |
| 11733025_at | AB026833.1 | | | CLCA2 | | 0.4783 | | 0.002340321 | |  |
| 11733091_a_at | NM_004616.2 | | | TSPAN8 | | 2.5854 | | 0.002862157 | |  |
| 11735140_at | NM_003811.3 | | | TNFSF9 | | 0.5617 | | 0.044378153 | |  |
| 11735987_at | NM_032935.2 | | | MT4 | | 0.6321 | | 0.047753856 | |  |
| 11737847_a_at | NM_001531.1 | | | MR1 | | 0.5784 | | 0.007443018 | |  |
| 11739094_a_at | L01639.1 | | | CXCR4 | | 2.3822 | | 0.022975705 | |  |
| 11739232_x_at | AA827918 | | | RASSF2 | | 0.8254 | | 0.041702338 | |  |
| 11740613_a_at | BC009006.1 | | | TMEM30A | | 0.5738 | | 0.03372852 | |  |
| 11740781_a_at | NM_153464.2 | | | ILF3 | | 1.7505 | | 0.038832919 | |  |
| 11741315_a_at | NM_001128305.1 | | | PLSCR4 | | 0.6335 | | 0.034071687 | |  |
| 11741555_s_at | NM_001127605.1 | | | LIPA | | 0.5861 | | 0.027844422 | |  |
| 11741581_s_at | NM_130798.1 | | | SNAP23 | | 0.6905 | | 0.029832396 | |  |
| 11741704_x_at | NM_003526.2 | | | HIST1H2BC /// HIST1H2BE /// HIST1H2BF /// HIST1H2BG /// HIST1H2BI | | 0.437 | | 0.00730483 | |  |
| 11743352_s_at | AB023194.1 | | | COBLL1 | | 0.542 | | 0.008683595 | |  |
| 11743353_s_at | BM975358 | | | STAT1 | | 0.5973 | | 0.019835313 | |  |
| 11744030_a_at | AI565078 | | | MMP7 | | 5.72 | | 0.03051889 | |  |
| 11744034_a_at | AL571788 | | | VASP | | 2.4126 | | 0.056241203 | |  |
| 11744162_a_at | DB361302 | | | DUSP4 | | 2.044 | | 0.022853971 | |  |
| 11744236_a_at | AW150758 | | | DDX60 | | 0.5386 | | 0.084090751 | |  |
| 11744434_a_at | AF307339.1 | | | PARP9 | | 0.5536 | | 0.005334423 | |  |
| 11744915_a_at | BQ009101 | | | NEGR1 | | 2.9132 | | 0.014156016 | |  |
| 11745652_s_at | M26481.1 | | | EPCAM | | 1.5627 | | 0.034327109 | |  |
| 11746088_a_at | DB350079 | | | IFI44 | | 0.3067 | | 0.019643111 | |  |
| 11748907_a_at | AK293357.1 | | | RARRES3 | | 0.6458 | | 0.005688589 | |  |
| 11749773_x_at | AK303893.1 | | | GBP1 | | 0.5578 | | 0.041891567 | |  |
| 11749922_x_at | BC065829.1 | | | TAGLN | | 0.64 | | 0.01822962 | |  |
| 11750531_a_at | NM_013258.3 | | | PYCARD | | 0.4457 | | 0.043407432 | |  |
| 11751651_s_at | AK300181.1 | | | PLSCR1 | | 0.4827 | | 0.008946378 | |  |
| 11752384_s_at | AK299396.1 | | | COL6A1 | | 0.6185 | | 0.024830538 | |  |
| 11753234_a_at | AK303598.1 | | | TAMM41 | | 1.5092 | | 0.002845593 | |  |
| 11753554_a_at | BC128163.1 | | | SPINK13 | | 0.4676 | | 0.004695121 | |  |
| 11753810_a_at | AF043341.1 | | | CCL5 | | 0.3804 | | 0.025494917 | |  |
| 11755110_a_at | AK126745.1 | | | LRIG1 | | 2.4721 | | 0.03637693 | |  |
| 11755147_s_at | BX640607.1 | | | STAT2 | | 0.6184 | | 0.023873146 | |  |
| 11755252_a_at | AK091761.1 | | | AMIGO2 | | 1.6183 | | 0.028686246 | |  |
| 11755587_a_at | X82200.1 | | | TRIM22 | | 0.58 | | 0.03398674 | |  |
| 11755819_a_at | AK125989.1 | | | DDX58 | | 0.5842 | | 0.018986327 | |  |
| 11756714_a_at | CR600533.1 | | | PPP2R2A | | 2.0744 | | 0.021177706 | |  |
| 11756820_a_at | AK308688.1 | | | IFIT1 | | 0.3554 | | 0.002881868 | |  |
| 11757033_a_at | DA534357 | | | IL13RA2 | | 3.1991 | | 0.005204142 | |  |
| 11757480_x_at | AW960588 | | | IFI27 | | 0.2965 | | 0.005286798 | |  |
| 11757702_s_at | BF063817 | | | DSC2 | | 0.6472 | | 0.113943123 | |  |
| 11758028_s_at | BQ184392 | | | FOXQ1 | | 1.5547 | | 0.052090294 | |  |
| 11758200_x_at | BM009647 | | | CKS1B | | 1.8096 | | 0.000427862 | |  |
| 11758750_x_at | BC083508.1 | | | YWHAZ | | 1.5905 | | 0.016531317 | |  |
| 11759022_s_at | BX436694 | | | ELK3 | | 2.4595 | | 0.022000032 | |  |
| 11760254_at | DB350079 | | | IFI44 | | 0.4997 | | 0.048410954 | |  |
| 11762785_x_at | AK301175.1 | | | ACTG1 | | 2.0552 | | 0.031054854 | |  |
| 11763168_s_at | BC117379.1 | | | ALG13 | | 1.5519 | | 0.017967558 | |  |
| **S1_4 Table. List of genes that are regulated by PYCARD-AS1 knockdown and reversed upon PYCARD knockdown (shAS-2/shS versus shAS-2)** | | | | | | | | | |  |
| **ProbeSetID** | | **Target transcript ID** | | | **Gene symbol** | **Fold change** | | **p value** | |  |
| 11715388_s_at | | NM_078467.1 | | | CDKN1A | 0.5918 | | 0.034096174 | |  |
| 11715394_s_at | | NM_004356.3 | | | CD81 | 2.0375 | | 0.002195484 | |  |
| 11715536_a_at | | NM_002168.2 | | | IDH2 | 0.5371 | | 0.015095967 | |  |
| 11715557_at | | CR597598.1 | | | QSOX1 | 0.6209 | | 0.009707114 | |  |
| 11715576_at | | AF136373.1 | | | RAC1 | 0.6248 | | 0.041322483 | |  |
| 11715620_a_at | | NM_001885.1 | | | CRYAB | 0.3971 | | 0.033710808 | |  |
| 11715670_a_at | | NM_003641.3 | | | IFITM1 | 0.4873 | | 0.007193642 | |  |
| 11715745_a_at | | NM_020444.3 | | | KIAA1191 | 1.817 | | 0.009573362 | |  |
| 11715793_a_at | | NM_001615.3 | | | ACTG2 | 0.2965 | | 0.000842383 | |  |
| 11716167_a_at | | NM_001144925.1 | | | MX1 | 0.5235 | | 0.005312705 | |  |
| 11716433_s_at | | NM_001037582.2 | | | SCD5 | 2.2911 | | 0.039205224 | |  |
| 11716876_a_at | | NM_003313.3 | | | TSTA3 | 0.503 | | 0.044000824 | |  |
| 11716895_s_at | | NM_005101.3 | | | ISG15 | 0.4251 | | 0.0185337 | |  |
| 11717212_a_at | | NM_002499.2 | | | NEO1 | 1.5201 | | 0.007179822 | |  |
| 11717473_s_at | | NM_000596.2 | | | IGFBP1 | 5.0682 | | 0.021188707 | |  |
| 11717935_a_at | | NM_024896.2 | | | ERMP1 | 1.6412 | | 0.020376521 | |  |
| 11718067_at | | AK291688.1 | | | MYO1E | 1.7891 | | 0.01059958 | |  |
| 11718231_x_at | | NM_018950.2 | | | HLA-F | 0.5294 | | 0.018935047 | |  |
| 11718916_a_at | | NM_004031.2 | | | IRF7 | 0.5097 | | 0.00600956 | |  |
| 11718986_a_at | | NM_022873.2 | | | IFI6 | 0.3191 | | 0.002635307 | |  |
| 11719222_at | | NM_003122.3 | | | SPINK1 | 1.9375 | | 0.021985953 | |  |
| 11719268_at | | NM_003280.2 | | | TNNC1 | 0.4838 | | 0.011870288 | |  |
| 11719447_s_at | | BC073163.1 | | | GBP2 | 0.4103 | | 0.039362932 | |  |
| 11719491_a_at | | NM_005533.3 | | | IFI35 | 0.313 | | 0.031412031 | |  |
| 11719764_s_at | | NM_212503.1 | | | CDK18 | 0.5664 | | 0.018605617 | |  |
| 11719833_at | | NM_005797.2 | | | MPZL2 | 0.6307 | | 0.035384394 | |  |
| 11720007_a_at | | NM_024636.2 | | | STEAP4 | 0.6014 | | 0.000800497 | |  |
| 11720028_x_at | | BC014514.1 | | | LDLR | 0.5266 | | 0.045815457 | |  |
| 11720209_at | | NM_006084.4 | | | IRF9 | 0.3211 | | 0.00600956 | |  |
| 11720669_at | | NM_003884.4 | | | KAT2B | 0.6385 | | 0.054362019 | |  |
| 11721562_a_at | | NM_006589.2 | | | FAM189B | 1.6277 | | 0.022089161 | |  |
| 11721864_s_at | | NM_207113.1 | | | SLC37A3 | 0.5939 | | 0.078464608 | |  |
| 11721874_at | | BC032839.2 | | | IFIT2 | 0.4999 | | 0.040347734 | |  |
| 11721897_a_at | | NM_024119.2 | | | DHX58 | 0.497 | | 0.002854208 | |  |
| 11721994_s_at | | NM_198183.1 | | | UBE2L6 | 0.4349 | | 0.022878553 | |  |
| 11722093_a_at | | NM_000943.4 | | | PPIC | 0.6183 | | 0.035231167 | |  |
| 11722215_at | | NM_019896.2 | | | POLE4 | 2.1875 | | 0.019203799 | |  |
| 11722244_at | | NM_020665.3 | | | TMEM27 | 0.6688 | | 0.029996346 | |  |
| 11722503_at | | NM_002214.2 | | | ITGB8 | 0.7102 | | 0.013452569 | |  |
| 11722909_a_at | | NM_178831.5 | | | GATS | 0.5865 | | 0.027202915 | |  |
| 11722963_a_at | | NM_016429.2 | | | COPZ2 | 0.4693 | | 0.040356398 | |  |
| 11723051_at | | NM_005130.3 | | | FGFBP1 | 0.4614 | | 0.007882442 | |  |
| 11723128_a_at | | NM_017912.3 | | | HERC6 | 0.5957 | | 0.009682267 | |  |
| 11723234_at | | AB116553.1 | | | IFI44L | 0.5981 | | 0.148700136 | |  |
| 11723390_a_at | | NM_003389.3 | | | CORO2A | 1.8607 | | 0.007359574 | |  |
| 11723854_at | | NM_017654.2 | | | SAMD9 | 0.4862 | | 0.009512947 | |  |
| 11724117_x_at | | AI038997 | | | SAMD9L | 0.5358 | | 0.020822955 | |  |
| 11724256_s_at | | NM_016816.2 | | | OAS1 | 0.3268 | | 0.000879312 | |  |
| 11724325_a_at | | NM_014857.3 | | | RABGAP1L | 0.4304 | | 0.033290606 | |  |
| 11724346_a_at | | NM_022168.2 | | | IFIH1 | 0.4192 | | 0.026321587 | |  |
| 11724378_s_at | | BC090931.1 | | | PAG1 | 0.4373 | | 0.014062957 | |  |
| 11724399_a_at | | NM_001012967.1 | | | DDX60L | 0.5404 | | 0.014098159 | |  |
| 11724463_a_at | | NM_001005340.1 | | | GPNMB | 0.4213 | | 0.01067366 | |  |
| 11724538_a_at | | BC021281.2 | | | ABCG2 | 0.4855 | | 0.009254806 | |  |
| 11724885_at | | NM_004669.2 | | | CLIC3 | 0.3611 | | 0.007209616 | |  |
| 11725188_a_at | | BX640971.1 | | | GPR126 | 0.5801 | | 0.026853023 | |  |
| 11725280_a_at | | NM_020226.3 | | | PRDM8 | 1.8854 | | 0.001660973 | |  |
| 11725951_at | | AF013591.1 | | | RIOK3 | 0.6286 | | 0.011400317 | |  |
| 11725964_a_at | | BC005256.1 | | | CAV2 | 0.7747 | | 0.030919263 | |  |
| 11725999_s_at | | AK290978.1 | | | FAS | 0.656 | | 0.051914031 | |  |
| 11726113_a_at | | NM_052943.3 | | | FAM46B | 0.551 | | 0.008047807 | |  |
| 11726201_a_at | | NM_016817.2 | | | OAS2 | 0.3318 | | 0.04995217 | |  |
| 11726364_x_at | | NM_003733.2 | | | OASL | 0.3763 | | 0.032022643 | |  |
| 11726479_a_at | | NM_002463.1 | | | MX2 | 0.5588 | | 0.001722648 | |  |
| 11726770_x_at | | BC073156.1 | | | XAF1 | 0.4094 | | 0.013383895 | |  |
| 11726838_s_at | | NM_173690.3 | | | SCAI | 1.7133 | | 0.007102538 | |  |
| 11727440_at | | BC007522.1 | | | LOXL4 | 0.5524 | | 0.04672951 | |  |
| 11727537_at | | NM_001870.2 | | | CPA3 | 2.0695 | | 0.003342197 | |  |
| 11727645_a_at | | NM_001105078.2 | | | MECOM | 1.7938 | | 0.012812438 | |  |
| 11728300_at | | NM_057749.1 | | | CCNE2 | 0.6058 | | 0.067366361 | |  |
| 11729258_at | | BC034991.2 | | | MNS1 | 1.7007 | | 0.024876878 | |  |
| 11730370_a_at | | BM992839 | | | EIF4E3 | 0.3852 | | 0.02991204 | |  |
| 11731137_a_at | | AF328296.1 | | | MYPN | 0.6549 | | 0.008065308 | |  |
| 11731606_at | | NM_001112706.2 | | | SCIN | 0.4636 | | 0.017078167 | |  |
| 11731657_s_at | | NM_001079874.1 | | | VAV3 | 0.5244 | | 0.007408109 | |  |
| 11731819_a_at | | NM_017752.2 | | | TBC1D8B | 0.7721 | | 0.034939548 | |  |
| 11731831_x_at | | NM_138413.3 | | | HOGA1 | 0.5437 | | 0.00068167 | |  |
| 11732111_a_at | | NM_014452.3 | | | TNFRSF21 | 3.2914 | | 0.020673265 | |  |
| 11732318_a_at | | BC032606.2 | | | KHDRBS3 | 1.8709 | | 0.017877567 | |  |
| 11733025_at | | AB026833.1 | | | CLCA2 | 0.4701 | | 0.039503136 | |  |
| 11733091_a_at | | NM_004616.2 | | | TSPAN8 | 2.2102 | | 0.015902766 | |  |
| 11735140_at | | NM_003811.3 | | | TNFSF9 | 0.5429 | | 0.01590933 | |  |
| 11735987_at | | NM_032935.2 | | | MT4 | 0.4591 | | 0.012588388 | |  |
| 11737847_a_at | | NM_001531.1 | | | MR1 | 0.6388 | | 0.038763666 | |  |
| 11739094_a_at | | L01639.1 | | | CXCR4 | 1.9851 | | 0.010296339 | |  |
| 11739232_x_at | | AA827918 | | | RASSF2 | 0.7406 | | 0.006730824 | |  |
| 11740613_a_at | | BC009006.1 | | | TMEM30A | 0.5748 | | 0.010917644 | |  |
| 11740781_a_at | | NM_153464.2 | | | ILF3 | 2.2213 | | 0.031759604 | |  |
| 11741315_a_at | | NM_001128305.1 | | | PLSCR4 | 0.593 | | 0.030072883 | |  |
| 11741555_s_at | | NM_001127605.1 | | | LIPA | 0.6457 | | 0.014096583 | |  |
| 11741581_s_at | | NM_130798.1 | | | SNAP23 | 0.6178 | | 0.002994197 | |  |
| 11741704_x_at | | NM_003526.2 | | | HIST1H2BC /// HIST1H2BE /// HIST1H2BF /// HIST1H2BG /// HIST1H2BI | 0.4668 | | 0.02263904 | |  |
| 11743352_s_at | | AB023194.1 | | | COBLL1 | 0.6276 | | 0.000380856 | |  |
| 11743353_s_at | | BM975358 | | | STAT1 | 0.5763 | | 0.039655668 | |  |
| 11744030_a_at | | AI565078 | | | MMP7 | 2.1846 | | 0.02654416 | |  |
| 11744034_a_at | | AL571788 | | | VASP | 2.162 | | 0.024626991 | |  |
| 11744162_a_at | | DB361302 | | | DUSP4 | 1.8359 | | 0.012306254 | |  |
| 11744236_a_at | | AW150758 | | | DDX60 | 0.4609 | | 0.008162565 | |  |
| 11744434_a_at | | AF307339.1 | | | PARP9 | 0.4281 | | 0.008611306 | |  |
| 11744915_a_at | | BQ009101 | | | NEGR1 | 3.1639 | | 0.003618638 | |  |
| 11745652_s_at | | M26481.1 | | | EPCAM | 1.5809 | | 0.012006022 | |  |
| 11746088_a_at | | DB350079 | | | IFI44 | 0.313 | | 0.031412031 | |  |
| 11748907_a_at | | AK293357.1 | | | RARRES3 | 0.5198 | | 0.035574696 | |  |
| 11749773_x_at | | AK303893.1 | | | GBP1 | 0.4522 | | 0.015038075 | |  |
| 11749922_x_at | | BC065829.1 | | | TAGLN | 0.5911 | | 0.049047676 | |  |
| 11750531_a_at | | NM_013258.3 | | | PYCARD | 0.6955 | | 0.032061638 | |  |
| 11751651_s_at | | AK300181.1 | | | PLSCR1 | 0.3755 | | PLSCR1 | |  |
| 11752384_s_at | | AK299396.1 | | | COL6A1 | 0.5523 | | 0.043976629 | |  |
| 11753234_a_at | | AK303598.1 | | | TAMM41 | 1.519 | | 0.031394698 | |  |
| 11753554_a_at | | BC128163.1 | | | SPINK13 | 0.426 | | 0.021985953 | |  |
| 11753810_a_at | | AF043341.1 | | | CCL5 | 0.286 | | 0.029914343 | |  |
| 11755110_a_at | | AK126745.1 | | | LRIG1 | 2.3453 | | 0.042687262 | |  |
| 11755147_s_at | | BX640607.1 | | | STAT2 | 0.5511 | | 0.020886784 | |  |
| 11755252_a_at | | AK091761.1 | | | AMIGO2 | 1.587 | | 0.014532442 | |  |
| 11755587_a_at | | X82200.1 | | | TRIM22 | 0.4713 | | 0.028432543 | |  |
| 11755819_a_at | | AK125989.1 | | | DDX58 | 0.4218 | | 0.020472606 | |  |
| 11756714_a_at | | CR600533.1 | | | PPP2R2A | 2.0284 | | 0.010247284 | |  |
| 11756820_a_at | | AK308688.1 | | | IFIT1 | 0.3128 | | 0.040347734 | |  |
| 11757033_a_at | | DA534357 | | | IL13RA2 | 2.307 | | 0.031630255 | |  |
| 11757480_x_at | | AW960588 | | | IFI27 | 0.2637 | | 0.003831215 | |  |
| 11757702_s_at | | BF063817 | | | DSC2 | 0.6583 | | 0.055722756 | |  |
| 11758028_s_at | | BQ184392 | | | FOXQ1 | 1.6784 | | 0.040804241 | |  |
| 11758200_x_at | | BM009647 | | | CKS1B | 1.8448 | | 0.009113474 | |  |
| 11758750_x_at | | BC083508.1 | | | YWHAZ | 1.5823 | | 0.028184615 | |  |
| 11759022_s_at | | BX436694 | | | ELK3 | 2.1537 | | 0.012316265 | |  |
| 11760254_at | | DB350079 | | | IFI44 | 0.313 | | 0.031412031 | |  |
| 11762785_x_at | | AK301175.1 | | | ACTG1 | 2.2208 | | 0.006351161 | |  |
| 11763168_s_at | | BC117379.1 | | | ALG13 | 2.2295 | | 0.058499908 | |  |
