# Supplementary material for: A long noncoding RNA distributed in both nucleus and cytoplasm operates in the PYCARD-regulated apoptosis by coordinating the epigenetic and translational regulation
Source: PLoS Genet. 2019 May 14;15(5):e1008144. doi: 10.1371/journal.pgen.1008144 (PMC6534332; doi:10.1371/journal.pgen.1008144)
Supplement: S2 Table — (DOCX) [file pgen.1008144.s007.docx]

| **S2 Table. Sequences of primers and oligos used in this study** | | |
| --- | --- | --- |
|  |  |  |
| **Name** | | **Sequences (5′ to 3′)** |
| **(F: forward, R: reverse)** | |  |
| **RACE and cloning of full-length PYCARD-AS1** | | |
| 3’-GSP-F1 | | GGTCCGTTGCCCTCCAGCAA |
| 3’-GSP-F2 | | GGCGCTTCCTTACTACACCCTTG |
| 5’-GSP-R1 | | CACCCATGAGCCCCTCTGCG |
| 5’-GSP-R2 | | ACCTCGCAGGCCAGCTGGAG |
| PYCARD-AS1-FL-F(F3) | | GCCTGTCCCTGCTGAACTTG |
| PYCARD-AS1-FL-R(R3) | | AATCTCCTGGAGGCTGTCTCTC |
| **RT-PCR and qRT-PCR** | | |
| 12S rRNA-F | ACTGCTCGCCAGAACACTACGA | |
| 12S rRNA-R | GTCTTTACGTGGGTACTTGCGCT | |
| U1 snRNA-F | TCCCAGGGCGAGGCTTATCCATTG | |
| U1 snRNA-R | GCGAACGCAGTCCCCCACTACCAC | |
| PYCARD-AS1-F | CCTTAGGATCCTCTCCCACATGTCG | |
| PYCARD-AS1-R | CTAGGAAGCATCTGGCAAGGTTTCG | |
| GAPDH-F | GTCAAGGCTGAGAACGGGAAGCT | |
| GAPDH-R | GCCTTCTCCATGGTGGTGAAGA | |
| pre-tRNAtyr-F | CCTTCGATAGCTCAGCTGGTAGAG | |
| pre-tRNAtyr-R | AAAAAACCGCACTTGTCTCCTTCG | |
| 45S pre-rRNA-F | CCGTCCGTCCGTCGTCCTCCTCGC | |
| 45S pre-rRNA-R | TGTACCGGCCGTGCGTACTTAGAC | |
| ACTB-F | GCACTCTTCCAGCCTTCCTTC | |
| ACTB-R | TTGGCGTACAGGTCTTTGCGGA | |
| XIST-F | TGATCCCATTGAAGATACCACGCTG | |
| XIST-R | TGGCAACCCATCCAAGTAGATTAGC | |
| 18S rRNA-F | ACACGGACAGGATTGACAGA | |
| 18S rRNA-R | GGACATCTAAGGGCATCACA | |
| PYCARD-F | GCCTGCACTTTATAGACCAGCAC | |
| PYCARD-R | GAAGAGCTTCCGCATCTTGCTTG | |
| FUS-F | CAGCAGAACCAGTACAACAGCAG | |
| FUS-R | TGCTGTCCATAGCCACCGCTG | |
| TRIM72-F | TGTGCTGGAGCATCAGCTGGT | |
| TRIM72-R | CCGTACACGCTCTGCCTCGCG | |
| PYDC1-F | ACTACGCAGCCGAGCTCGTCGT | |
| PYDC1-R | GGCTGGCTTCACAGGCGTTGCAT | |
| FOXQ1-F | CGGGCATGAAGTTGGAGGTGTT | |
| FOXQ1-R | ATCTGAGCCCAGGGAGTCGTCT | |
| RASSF2-F | CTTTGGACAGAGGGTTTACTT | |
| RASSF2-R | GCCAAGTCTGAATAACATAACC | |
| TSPAN8-F | GGACAAGCCTGTAACGAATAG | |
| TSPAN8-R | AGATACCACATAGCCAGAACA | |
| VASP-F | GCTATTGCTGGAGCCAAACTCA | |
| VASP-R | GAAGTGGTCACCGAAGAAGACG | |
| PLSCR1-F | GCGCAAAGGTTACTCCCAGAC | |
| PLSCR1-R | GAGGTCCTTGGAATGCTGTCG | |
| TRIM22-F | CACCAAACATTCCGCATAAAC | |
| TRIM22-R | ATCCAGCACATTCACCTCACCTT | |
| DNMT1-F | TGGGCTGATGCAGGAGAAGATC | |
| DNMT1-R | TGAAGCGGTTCAAGTTGAGGCCA | |
| G9a-F | ATGGTCAGCCTGCTGCTGAGCA | |
| G9a-R | GCCCAGTGCAGGCAGATGTTCT | |
| 7SK-F | ATCGCCAGGGTTGATTCGGCTGAT | |
| 7SK-R | GGATGTGTCTGGAGTCTTGGAAGC | |
| **Bisulfite sequencing** | | |
| Me-PYCARD-F | GTGTAAGTTTAGAGATAAGTAG | |
| Me-PYCARD-R | CAACTTAAACTTCTTAAACTCCT | |
| Me-PYCARD-AS1-F | TTTATTGTATGTGGGGTTTTGGTGG | |
| Me-PYCARD-AS1-R | AACAAATCCTTACAAATCCAATTCC | |
| **ChIP assay** | | |
| PYCARD-1F | CAGTTAAGGTCTCCGATGCTCATAGGC | |
| PYCARD-1R | CACTGTTCCTTGTAGTCATACGACCCTG | |
| PYCARD-2F | CCCCCTTGGGAAGTAGAGTCAGGAT | |
| PYCARD-2R | TGCTTCCTAGGGGAGCCAGAATTTG | |
| PYCARD-3F | GCACTCTCTGCCCTTTTGTACAAC | |
| PYCARD-3R | CCGAACCTTAGGATCCTCTCC | |
| PYCARD-4F | ATAAACCCAGTGAGAGCCAGCCCAG | |
| PYCARD-4R | AGGCGCTTCCTTACTACACCCTTGG | |
| PYCARD-5F | TGACCGCCGAGGAGCTCAAGAAGTT | |
| PYCARD-5R | CGTAGGTCTCCAGGTAGAAGCTGAC | |
| PYCARD-6F | GTTCCTCCTACCCCTAAACAAAGC | |
| PYCARD-6R | CCTGGAAGGATATGGGCCAAGTGA | |
| PYCARD-7F | GAGACAATATTACCCTCATCCCACT | |
| PYCARD-7R | TGTCCCTGCTGAACTTGAGTTCTTC | |
| PYCARD-8F | GACGGATGAGCAGTACCAGGCAGT | |
| PYCARD-8R | AGTCCTTGCAGGTCCAGTTCCAGG | |
| PYCARD-9F | ACTTGTGTGTTTTCCTGCTTCTAGC | |
| PYCARD-9R | AGGAGGGATATTCCATGGCGATACC | |
| PYCARD-10F | AGGCATGTACCAAAGGGCGCAA | |
| PYCARD-10R | TGGTGACTTGTGCTCTTCTGTCCT | |
| PYCARD-11F | CCAACCACCAAAGCAGATTTTCAGG | |
| PYCARD-11R | ACTCCAGTCTGGGTGACACAGCGAG | |
| **ChIRP qPCR** | | |
| PYCARD promoter-F | ATAAACCCAGTGAGAGCCAGCCCAG | |
| PYCARD promoter-R | AGGCGCTTCCTTACTACACCCTTGG | |
| ACTB promoter-F | ACACCACACTCTACCTCTCAAGCCC | |
| ACTB promoter-R | CTTCAGAGCAACTGCCCTGAAAGC | |
| **Oligos for ChIRP assay** | | |
| Antisense probes | TCCAATAAACCCAGTGAGAG | |
|  | GAAATCCGAGGTTCTAAGCC | |
|  | TTCACGCTTCTAGCTGTCAT | |
|  | CAGTATGTGGAATTGAGGGA | |
|  | TATAAGCACCGGAGGGTTAT | |
|  | TAGGAAGCATCTGGCAAGGT | |
|  | CTGGAGGCTGTCTCTCTTTC | |
|  | ATGGCTTCAAGTTCATGGTC | |
| Sense probes | CTCTCACTGGGTTTATTGGA | |
|  | GGCTTAGAACCTCGGATTTC | |
|  | ATGACAGCTAGAAGCGTGAA | |
|  | TCCCTCAATTCCACATACTG | |
|  | ATAACCCTCCGGTGCTTATA | |
|  | ACCTTGCCAGATGCTTCCTA | |
|  | GAAAGAGAGACAGCCTCCAG | |
|  | GACCATGAACTTGAAGCCAT | |
| **Primer walk** | | |
| PYCARD-AS1-F1 | GCCTGTCCCTGCTGAACTTG | |
| PYCARD-AS1-F2 | CGTAGGTCTCCAGGTAGAAGCTGAC | |
| PYCARD-AS1-F3 | CACCGACAGCAGCTTCAGCT | |
| PYCARD-AS1-F4 | TTCTTGAGCTCCTCGGCGGT | |
| PYCARD-AS1-F5 | CCACTCTGGTCTCCCGACTC | |
| PYCARD-AS1-F6 | ACTACACCCTTGGTCCCCTC | |
| PYCARD-AS1-R | GAGGGTTATCCCCATGTCGTAGA | |
| **RIP-based RNA mapping assay** | | |
| PYCARD-AS1-1F | GCCTGTCCCTGCTGAACTTG | |
| PYCARD-AS1-1R | ACTGCATGTGGGGTCCTGGT | |
| PYCARD-AS1-2F | CGTAGGTCTCCAGGTAGAAGCTGAC | |
| PYCARD-AS1-2R | CGCCGAGGAGCTCAAGAAGTT | |
| PYCARD-AS1-3F | CACCGACAGCAGCTTCAGCT | |
| PYCARD-AS1-3R | AGCCGACTTCCTCCTGGTC | |
| PYCARD-AS1-4F | TTCTTGAGCTCCTCGGCGGT | |
| PYCARD-AS1-4R | GGAGGGGACCAAGGGTGTAGT | |
| PYCARD-AS1-5F | CCACTCTGGTCTCCCGACTC | |
| PYCARD-AS1-5R | GGAGGGGACCAAGGGTGTAGT | |
| PYCARD-AS1-6F | ACTACACCCTTGGTCCCCTC | |
| PYCARD-AS1-6R | GCAAGCCCAGAGACAAGCAG | |
| PYCARD-AS1-7F | CCTGCTTGTCTCTGGGCTTG | |
| PYCARD-AS1-7R | TTCGGGGTTCTAGAAATCCG | |
| PYCARD-AS1-8F | TTCTAGAACCCCGAAACCTCC | |
| PYCARD-AS1-8R | CAGTATGTGGAATTGAGGGAGC | |
| PYCARD-AS1-9F | GCTAGAAGCGTGAAGCTCCC | |
| PYCARD-AS1-9R | TAAGCACCGGAGGGTTATCC | |
| PYCARD-AS1-10F | TCTACGACATGGGGATAACCC | |
| PYCARD-AS1-10R | AATCTCCTGGAGGCTGTCTCTC | |
| **RNase-ChIP assays** | | |
| PYCARD promoter-F | ATAAACCCAGTGAGAGCCAGCCCAG | |
| PYCARD promoter-R | AGGCGCTTCCTTACTACACCCTTGG | |
| ACTB promoter-F | ACACCACACTCTACCTCTCAAGCCC | |
| ACTB promoter-R | CTTCAGAGCAACTGCCCTGAAAGC | |
| KCNQ1 promoter-F | ACTCCTCAAGGTCGGCTGAGACTCG | |
| KCNQ1 promoter-R | ACCGCCACCGTCACCTTCCTATCCT | |
| BECN1 promoter-F | CCTCCCCGTATCATACCATTCCT | |
| BECN1 promoter-R | CCTTCCACATTCTTGACCACCCT | |
| CDKN1C promoter-F | CCACGATGGAGCGTCTTGTC | |
| CDKN1C promoter-R | TCGTAATCCCAGCGGTTCTG | |
| CDH1 promoter-F | CCCATCTCCAAAACGAACAAACA | |
| CDH1 promoter-R | ATAGACGCGGTGACCCTCTAGCC | |
| **RNase-A assay** | | |
| PYCARD-AS1-1F | GAGGGTTATCCCCATGTCGTAGA | |
| PYCARD-AS1-1R | ATGACAGCTAGAAGCGTGAAGCT | |
| PYCARD-AS1-2F | CACCGACAGCAGCTTCAGCT | |
| PYCARD-AS1-2R | AGCCGACTTCCTCCTGGTC | |
| **Oligos encoding shRNAs** | | |
| shPYCARD-AS1-1 sense | CCGGAATCTTGGAATCATGACAGCTCTCGAGAGCTGTCATGATTCCAAGATTTTTTTG | |
| shPYCARD-AS1-1 antisense | AATTCAAAAAAATCTTGGAATCATGACAGCTCTCGAGAGCTGTCATGATTCCAAGATT | |
| shPYCARD-AS1-2 sense | CCGGAAGGCGCTTCCTTACTACACCCTCGAGGGTGTAGTAAGGAAGCGCCTTTTTTTG | |
| shPYCARD-AS1-2 antisense | AATTCAAAAAAAGGCGCTTCCTTACTACACCCTCGAGGGTGTAGTAAGGAAGCGCCTT | |
| shPYCARD-AS1-3 sense | CCGGAAGGGCAGAGAGTGCAAATCTCTCGAGAGATTTGCACTCTCTGCCCTTTTTTTG | |
| shPYCARD-AS1-3 antisense | AATTCAAAAAAAGGGCAGAGAGTGCAAATCTCTCGAGAGATTTGCACTCTCTGCCCTT | |
| shPYCARD sense | CCGGGCCCACCAACCCAAGCAAGATCTCGAGATCTTGCTTGGGTTGGTGGGCTTTTTG | |
| shPYCARD antisense | AATTCAAAAAGCCCACCAACCCAAGCAAGATCTCGAGATCTTGCTTGGGTTGGTGGGC | |
| shDNMT1 sense | CCGGGCCCAATGAGACTGACATCAACTCGAGTTGATGTCAGTCTCATTGGGCTTTTTG | |
| shDNMT1 antisense | AATTCAAAAAGCCCAATGAGACTGACATCAACTCGAGTTGATGTCAGTCTCATTGGGC | |
| shG9a sense | CCGGCGAGAGAGTTCATGGCTCTTTCTCGAGAAAGAGCCATGAACTCTCTCGTTTTTG | |
| shG9a antisense | AATTCAAAAACGAGAGAGTTCATGGCTCTTTCTCGAGAAAGAGCCATCAACTCTCTCG | |
| shNC sense | CCGGGCGCGATAGCGCTAATAATTTCTCGAGAAATTATTAGCGCTATCGCGCTTTTTG | |
| shNC antisense | AATTCAAAAAGCGCGATAGCGCTAATAATTTCTCGAGAAATTATTAGCGCTATCGCGC | |
